# The Genome of *Rhyzopertha dominica* (Fab.) (Coleoptera: Bostrichidae): Adaptation for Success

**DOI:** 10.3390/genes13030446

**Published:** 2022-02-28

**Authors:** Brenda Oppert, Anna Muszewska, Kamil Steczkiewicz, Eva Šatović-Vukšić, Miroslav Plohl, Jeffrey A. Fabrick, Konstantin S. Vinokurov, Igor Koloniuk, J. Spencer Johnston, Timothy P. L. Smith, Raul Narciso C. Guedes, Walter R. Terra, Clélia Ferreira, Renata O. Dias, Konstantin A. Chaply, Elena N. Elpidina, Valeriia F. Tereshchenkova, Robert F. Mitchell, Audra J. Jenson, Rachel McKay, Tisheng Shan, Xiaolong Cao, Zelong Miao, Chao Xiong, Haobo Jiang, William R. Morrison, Sergey Koren, David Schlipalius, Marcé D. Lorenzen, Raman Bansal, Yu-Hui Wang, Lindsey Perkin, Monica Poelchau, Kenlee Friesen, Morgan L. Olmstead, Erin Scully, James F. Campbell

**Affiliations:** 1 USDA ARS Center for Grain and Animal Health Research, 1515 College Ave., Manhattan, KS 66502, USA; william.morrison@usda.gov (W.R.M.III); ken.friesen@usda.gov (K.F.); morgan.olmstead@usda.gov (M.L.O.); erin.scully@usda.gov (E.S.); james.campbell@usda.gov (J.F.C.); 2 Institute of Biochemistry and Biophysics, Polish Academy of Sciences, Pawinskiego 5A, 02-106 Warsaw, Poland; ania.muszewska@gmail.com (A.M.); kamil.steczkiewicz@gmail.com (K.S.); 3 Division of Molecular Biology, Ruđer Bošković Institute, Bijenička 54, 10000 Zagreb, Croatia; esatovic@irb.hr (E.Š.-V.); plohl@irb.hr (M.P.); 4 USDA ARS U.S. Arid-Land Agricultural Research Center, Maricopa, AZ 85138, USA; jeff.fabrick@usda.gov; 5 Institute of Entomology, Biology Centre of the Czech Academy of Sciences, Branišovská 1160/31, 370 05 České Budejovice, Czech Republic; orchesia@gmail.com; 6 Institute of Plant Molecular Biology, Biology Centre of the Czech Academy of Sciences, Branišovská 1160/31, 370 05 České Budejovice, Czech Republic; koloniuk@umbr.cas.cz; 7 Department of Entomology, Texas A&M University, College Station, TX 77843, USA; spencerj@tamu.edu; 8 USDA ARS U.S. Meat Animal Research Center, Clay Center, NE 68933, USA; tim.smith2@usda.gov; 9 Departamento de Entomologia, Universidade Federal de Viçosa, Viçosa 36570-900, MG, Brazil; guedes@ufv.br; 10 Department of Biochemistry, Institute of Chemistry, University of São Paulo, Av. Prof. Lineu Prestes 748, São Paulo 05508-000, SP, Brazil; warterra@iq.usp.br (W.R.T.); clfterra@iq.usp.br (C.F.); 11 Institute of Biological Sciences, Federal University of Goiás, Av. Esperança s/n, Goiânia 74690-900, GO, Brazil; renata_dias@ufg.br; 12 Faculty of Bioengineering and Bioinformatics, Lomonosov Moscow State University, 119991 Moscow, Russia; k.chaply@gmail.com; 13 A.N. Belozersky Institute of Physico-Chemical Biology, Lomonosov Moscow State University, 119991 Moscow, Russia; elp@belozersky.msu.ru; 14 Department of Chemistry, Lomonosov Moscow State University, 119991 Moscow, Russia; v.tereshchenkova@gmail.com; 15 Department of Biology, University of Wisconsin Oshkosh, Oshkosh, WI 54901, USA; mitchellr@uwosh.edu (R.F.M.); jensonaudra@gmail.com (A.J.J.); rmckay20@alumni.uwosh.edu (R.M.); 16 Department of Entomology and Plant Pathology, Oklahoma State University, Stillwater, OK 74078, USA; tisheng.shan@okstate.edu (T.S.); xiaolong.cao@okstate.edu (X.C.); zelong.miao@okstate.edu (Z.M.); chaxion@okstate.edu (C.X.); haobo.jiang@okstate.edu (H.J.); 17 Genome Informatics Section, Computational and Statistical Genomics Branch, National Human Genome Research Institute, National Institutes of Health, Bethesda, MD 20892, USA; sergey.koren@nih.gov; 18 School of Biological Sciences, The University of Queensland, Brisbane 4072, QLD, Australia; david.schlipalius@uq.edu.au; 19 Department of Entomology and Plant Pathology, North Carolina State University, Raleigh, NC 27695, USA; mdlorenz@ncsu.edu (M.D.L.); ywang259@ncsu.edu (Y.-H.W.); 20 USDA Agricultural Research Service, Commodity Protection and Quality Research, 9611 S. Riverbend Ave., Parlier, CA 93648, USA; raman.bansal@usda.gov; 21 USDA Agricultural Research Service, Insect Control and Cotton Disease Research, 2771 F and B Road, College Station, TX 77845, USA; lindsey.perkin@usda.gov; 22 USDA Agricultural Research Service, National Agricultural Library, 10301 Baltimore, Ave., Beltsville, MD 20705, USA; monica.poelchau@usda.gov

**Keywords:** Bostrichidae, insect reference genome, insecticide resistance, insect genetics, lesser grain borer, *Rhyzopertha dominica*

## Abstract

The lesser grain borer, *Rhyzopertha dominica* (F.) (Coleoptera: Bostrichidae), is a major global pest of cereal grains. Infestations are difficult to control as larvae feed inside grain kernels, and many populations are resistant to both contact insecticides and fumigants. We sequenced the genome of *R. dominica* to identify genes responsible for important biological functions and develop more targeted and efficacious management strategies. The genome was assembled from long read sequencing and long-range scaffolding technologies. The genome assembly is 479.1 Mb, close to the predicted genome size of 480.4 Mb by flow cytometry. This assembly is among the most contiguous beetle assemblies published to date, with 139 scaffolds, an N_50_ of 53.6 Mb, and L_50_ of 4, indicating chromosome-scale scaffolds. Predicted genes from biologically relevant groups were manually annotated using transcriptome data from adults and different larval tissues to guide annotation. The expansion of carbohydrase and serine peptidase genes suggest that they combine to enable efficient digestion of cereal proteins. A reduction in the copy number of several detoxification gene families relative to other coleopterans may reflect the low selective pressure on these genes in an insect that spends most of its life feeding internally. Chemoreceptor genes contain elevated numbers of pseudogenes for odorant receptors that also may be related to the recent ontogenetic shift of *R. dominica* to a diet consisting primarily of stored grains. Analysis of repetitive sequences will further define the evolution of bostrichid beetles compared to other species. The data overall contribute significantly to coleopteran genetic research.

## 1. Introduction

**Figure 1:**
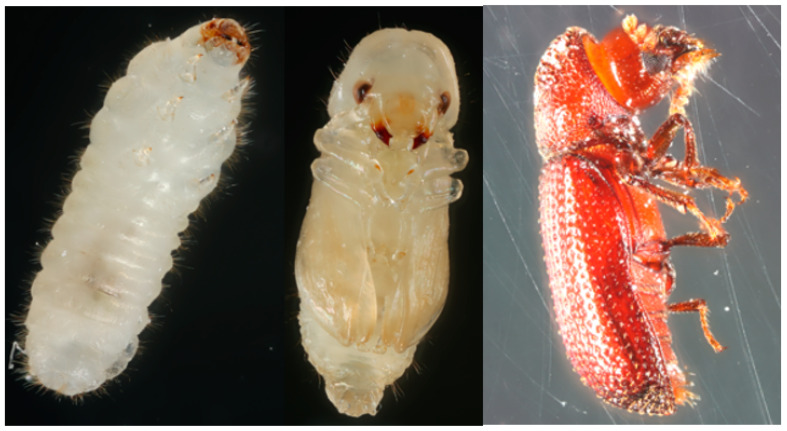
Photographs of different life stages of *R. dominica.* Left, larva; middle, pupa, right, adult.

The family Bostrichidae mainly consists of species of wood-boring insects that feed on living trees, dead branches, and wood products. The species *Rhyzopertha dominica* (F.) (Coleoptera: Bostrichidae) (lesser grain borer; Figure 1) was reported under the bark of *Quercus suber* (cork oak) and *Cytisus spinosus* (spiny broom) as early as 1849 [1] and more recently in other wooded environments [2,3,4]. The origin of *R. dominica* is speculated to be India, and most early citations were from India and Australia [5]. Currently the insect has a cosmopolitan distribution and has been found in wood-based materials, leather stuffing, pharmaceuticals, and mud plaster [6]. *Rhyzopertha dominica* also can be found in a variety of non-agricultural seed and fruits, with damaged acorns being a particularly favorable host [6,7,8]. 

*Rhyzopertha dominica* has adapted to a diet of cereal grains and subsequently evolved into a major global pest of stored grains including wheat, rice, and corn [6]. Cereal grains are now the predominant hosts for *R. dominica*. This insect lays its eggs outside grain kernels, and early instar larvae bore into the kernels to complete their development internally. The adult beetle emerges from the grain kernel and continues to feed on nearby grain throughout its lifespan, creating substantial damage to stored grain.

The remarkable adaptation of *R. dominica* to diverse environments, along with its strong dispersal capacity and ability to use food resources in landscapes surrounding food storage facilities, has reduced the effectiveness of control efforts [9], similar to other stored-product bostrichids, such as the larger grain borer, *Prostephanus truncates* [10]. Phosphine fumigation has successfully protected stored grain, but this approach has been complicated by the emergence of resistant populations on multiple continents including North America [11], South America [12], Africa [13], and Australia [14]. Phosphine resistance is an inherited trait that results from two incompletely recessive variants in the genes *rph1* and *rph2* [14], which act synergistically to increase phosphine resistance [15]. The *rph1* gene encodes a cytochrome b5-related fatty acid desaturase and is the main driver of low-level resistance [14], and loss of function mutations are associated with this phenotype. The *rph2* gene encodes dihydrolipoamide dehydrogenase (DLD) that participates in four steps of core metabolism [16]. The DLD enzyme is highly conserved and essential to life, and specific resistance variants are the main driver of strong resistance when combined with the *rph1* gene. 

Contact insecticides applied as grain protectants also can be used to prevent and manage infestations of *R. dominica* [17], including newer pyrethroid insecticides such as deltamethrin [18] and the biological pesticide spinosad [19]. In addition, the insect growth regulator methoprene can effectively control *R. dominica* if larvae are exposed before they enter the kernel [20]. However, use of the organophosphate insecticide malathion led to widespread issues with resistance several decades ago [21], and it is likely that resistance to other insecticides will continue to evolve in these beetles.

Understanding of the underlying mechanisms of resistance to grain protectants in *R. dominica* is largely limited to *in vivo* studies of insecticide synergists that inhibit detoxification enzymes, and a few *in vitro* studies of purified enzymes [22,23]. Resistance of *R. dominica* to organophosphate insecticides, a group of potent acetycholinesterase inhibitors, involves both detoxification enzymes, particularly phosphotriesterases and mainly altered acetycholisterase [23].

Grain protectants of more recent use, spinosad and methoprene, were the target of molecular studies of the underlying mechanisms of insecticide resistance [24,25]. The bioinsecticide spinosad is an actinomycete fermentation product acting as a nicotinic acetylcholine receptor (allosteric) modulator, while methoprene is a juvenile hormone analogue and thus an insect growth regulator. Spinosad resistance was recently associated with mutations leading to reduced expression of the nicotinic acetylcholinesterase receptor subunit α6, and consequently target site altered sensitivity, in a Taiwanese strain of *R. dominica* [24]. A recent transcriptomic analysis of methoprene resistance in *R. dominica* recognized the potential role of a set of differentially expressed genes related to cytochrome P450 (particularly *CYP6BQ11* and *CYP6RU* (Clan 3) and *CYP3747A* (Clan 4)), which can be potentially mitigated with piperonyl butoxide, a known inhibitor of P450 [25]. 

In addition to its diverse resistance mechanisms, *R. dominica* also responds to a variety of semiochemicals from conspecifics and the environment. Both males and females readily respond to the male-produced aggregation pheromone, which gives infested grain a characteristic sweetish odor [26]. The two-component *R. dominica* aggregation pheromone was identified as (*S*)-(+)-1-methylbutyl-(*E*)-2-methyl-2-pentenoate (dominicalure 1) and (*S*)-(+)-1-methylbutyl-(*E*)-2,4-dimethyl-2-pentenoate (dominicalure 2) [27] and was effective at trapping both sexes individually and in combination in the field. Volatiles produced by stored grains play a key role in the pheromone biology; while the attractiveness of host volatiles alone is unclear [28,29], adults produce more pheromone on a diet of wheat [30], are attracted to conspecifics feeding on wheat [31], and move toward infested kernels in the grain mass [32]. 

Chemosensory biology is mediated by three diverse gene families of odorant (OR), gustatory (GR), and ionotropic (IR) receptors, which are expressed in sensory neurons and are the primary means by which insects detect odors and tastes in their environment (reviewed in [33]). Genomes of beetles usually encode hundreds of these chemoreceptors [34], and while their abundance appears to be positively correlated with the host range of the species [35], their ligands are almost entirely unknown. A few functional studies have characterized pheromone-sensitive ORs from the families Cerambycidae and Curculionidae [36,37,38], various ORs sensitive to host plant volatiles [39,40,41], and a GR sensitive to sugar alcohols in *T. castaneum* [42]. Thus, the chemosensory genomics of beetles is mostly described from the size of the repertoire and its phylogeny relative to other species, and function is inferred from conserved homologs in other insect orders and/or expression data [34]. Chemosensory genes were annotated previously from an antennal transcriptome of *R*. *dominica* [43], which identified six ORs and eight IRs, as well as a few supporting proteins (16 odorant binding proteins, 8 chemosensory proteins, and 5 sensory neuron membrane proteins). Apart from this study, relatively little research has investigated the genetic underpinnings of olfaction in *R. dominica*. 

Here, we present a genome assembly of *R. dominica* to provide a basis for identification of genes related to adaptation and insecticide resistance. This reference assembly will improve detection and monitoring of phosphine resistance and provide a baseline to study development of resistance to other insecticides. We describe annotation of the genome with emphasis on genes and gene families relevant to the life cycle and adaptation of the insect as well as genes known or predicted to be involved in insecticide resistance. Moreover, *R. dominica* represents one of more than 700 species in the Bostrichidae family and thus the genome sequence improves our ability to study the evolution and biology of these beetles.

## 2. Methods

### 2.1. Insect Strain

A laboratory colony of *R. dominica* was established from grain bins in Eastern Kansas and has been maintained at CGAHR since 1972. To simplify assembly, a single pair subculture from the laboratory colony was inbred for 20 generations, from December 2009 to March 2012, designated “LGB Inbred D”, and was used for all genome DNA extractions. 

### 2.2. Photography

*Rhyzopertha dominica* insects were point-mounted on insect pins (#1, Bioquip, Rancho Dominguez, CA, USA), pulled from the colony as adults, or as pupae or larvae developing within a single grain kernel. Developing life stages were excised from the kernel, and along with unmounted specimens were placed on a series of two back-to-back petri dishes (35 mm × 15 mm), which comprised a platform on which to photograph the life stages. Pictures of the life stages were taken using a DSLR camera (EOS 7D Mark II, Canon, Tokyo, Japan) mounted to 3D imaging StackShot (CogniSys, Inc., Traverse City, MI, USA) equipped with a dual flash (MT-26EX-RT, Canon, Tokyo, Japan). A macro lens (MP-E 65 mm f/2.8, Canon, Tokyo, Japan) was used to focus on the life stages at 1–5-fold a 1:1 life size ratio. Light was diffused using a partially cut occluded plastic jar (15.2 cm × 7.6 cm D:H). A stack of between 25–45 slices was taken, depending on the size of the specimen, then combined using image montage software (Helicon Focus, Helicon Soft Ltd., Kharkiv, Ukraine) to create a single image in-focus throughout the range of the specimen. 

### 2.3. Measurement of Genome Size

The size of the *R. dominica* genome was previously estimated at 476 Mb [44]. The sex of the sample and standard were not reported in that study, and so we measured the genome size of males and females from the sequenced strain, LGB inbred D, by flow cytometry as described in [45]. Nuclei were released from tissues in the head of a *R. dominica* and a *Drosophila virilis* female (1C = 328 Mbp) with 15 strokes of the “A” pestle in a 2 ml Kontes Dounce tube containing 1 mL of Galbraith buffer. The released nuclei were filtered through 40 µ nylon, stained for 3 h in the cold and dark with 0.25 mg/mL of propidium iodide, and the relative fluorescence of the sample and standard nuclei scored as a mean channel number using a CytoFlex flow cytometer (Beckman Coulter, Pasadena, CA, USA). At least 1000 nuclei were scored under the 2C peaks of the sample and the standard, with a C.V. < 2.0 for each 2C peak. The Mbp of DNA in each sample was scored as the ratio of the mean channel number of the 2C sample nuclei to the mean channel number of the 2C standard nuclei times the 1C genome size of the standard.

### 2.4. Extraction of Nucleic Acids, Sequencing, and Assembly

#### 2.4.1. Genome Sequencing and Assembly

Different life stages were evaluated for optimal extraction of gDNA. *R. dominica* pupae dissected from wheat kernels gave the best high quality long gDNA. For short- and long read sequencing, genomic DNA was extracted from 20 mixed-sex *R. dominica* pupae of the LGB Inbred D strain using Quick-DNA™ Tissue/Insect Miniprep Kit (Zymo Research, Irvine, CA, USA). Precautions were taken to avoid shearing, such as gently inverting to mix, and use of wide bore pipettes for all steps. 

*Rhyzopertha dominica* gDNA was transported by laboratory personnel to Clay Center, NE, to avoid shearing that may occur during courier transport. Size selection of a portion of the gDNA for long read sequencing was performed with a BluePippin instrument (Sage Science Inc., Beverly, MA, USA) using a 15 kb lower cutoff value. Libraries for long read sequencing on the RSII platform were constructed using the SMRTbell™ Template Prep Kit 1.0 as recommended by the manufacturer (Pacific Biosciences, Menlo Park, CA, USA). Four libraries were prepared from the same gDNA and sequenced on sixteen SMRT cells of the RSII using P5/C3 and P6/C4 chemistry (eight cells each). 

For short-read sequencing, a portion of the gDNA was sheared using a Covaris S220 for 400 base fragments as recommended by the manufacturer (Covaris Inc. Woburn, MA, USA). A library was prepared with the TruSeq^®^ PCR-Free library preparation kit (Illumina Inc., San Diego, CA, USA) and sequenced on a MiSeq instrument using a 2 × 300 base paired read v3 reagent kit. Reads were submitted to NCBI SRA accession SUB10415981. 

The genome was assembled from PacBio only reads using CANU v1.3 (genomeSize = 476 m with default settings) on cloud HPC (Nimbix, Richardson, TX, USA) (Table 1) and polished with long read data in Arrow (SMRT Link 3.1.1, PacBio). *Rhyzopertha dominica* gDNA (extracted as previously described) was shipped to Dovetail Genomics for sequencing and scaffolding. The draft assembly was scaffolded by Chicago long-range data (Dovetail Genomics). A Chicago library was prepared as described previously [46]. Briefly, ~500 ng of HMW gDNA was reconstituted into chromatin *in vitro* and fixed with formaldehyde. Fixed chromatin was digested with *Dpn II*, the 5’ overhangs filled in with biotinylated nucleotides, and then free blunt ends were ligated. After ligation, crosslinks were reversed and the DNA purified from protein. Purified DNA was treated to remove biotin that was not internal to ligated fragments. The DNA was then sheared to ~350 bp mean fragment size and sequencing libraries were generated using NEBNext Ultra enzymes and Illumina-compatible adapters. Biotin-containing fragments were isolated using streptavidin beads before PCR enrichment of each library. The libraries were sequenced on an Illumina HiSeq X to produce 53 million 2 × 150 bp paired-end reads. 

The Chicago-scaffolded assembly was assembled with Mi-Seq short reads in SeqManNGen (DNAStar Lasergene v12, Madison, WI, USA). This hybrid assembly was scaffolded with Hi-C data (Dovetail Genomics). A Dovetail HiC library was prepared in a similar manner as described previously [48]. The libraries were sequenced on an Illumina HiSeq X to produce 89 million 2 × 150-bp paired-end reads. 

The input *de novo* assembly (our draft assembly from CANU), Chicago library reads, and Dovetail HiC library reads were used as input data for HiRise, a software pipeline designed specifically for using proximity ligation data to scaffold genome assemblies [49]. An iterative analysis was conducted. First, Chicago library sequences were aligned to the draft input assembly using a modified SNAP read mapper [50]. The separations of Chicago read pairs mapped within draft scaffolds were analyzed by HiRise to produce a likelihood model for genomic distance between read pairs, and the model was used to identify and break putative misjoins, to score prospective joins, and make joins above a threshold. After aligning and scaffolding Chicago data, Dovetail HiC library sequences were aligned and scaffolded following the same method (Figure S1). 

The final genome assembly for *R. dominica* was submitted to NCBI accession SUB2507831.

#### 2.4.2. Transcriptome Sequencing and Gene Expression Analysis

To obtain larvae for dissection, infested wheat kernels with 3–4-week-old *R. dominica* larvae (30 °C, 65% R.H.; tempered wheat; 100+ adults/jar/week) were X-rayed as in [51]. A detailed description of the dissection procedure with diagrams is found in File S1.

RNA was extracted and sequenced as in [52]. Briefly, RNA was collected as three independent biological replicates from each larval tissue (head, gut, carcass). The tissue was pulverized in TRIZOL (BulletBlender, Next Advance Inc., Averill Park, NY, USA) at speed 8 for 2 min with RNAse-free ziroconium oxide beads. RNA extraction and purification were with a Zymo mini prep kit (Irvine, CA, USA). DIRECTbeads (Agilient, Santa Clara, CA, USA) were used to isolate polyA mRNA from total RNA, and libraries were made with a 200 bp RNA-Seq v2 kit (Life Technologies, Grand Island, NY, USA). Samples were sequenced on 318v2 chips on the Ion Torrent Personal Genome Machine (PGM, Life Technologies). Total reads per sample were: head—216,180; gut—263,938; carcass—268,508. Reads for the *R. dominica* head, gut, carcass data were submitted to SRA SUB6755681.

Differential expression of transcriptome data was determined using ArrayStar (DNAStar Lasergene). Reads were mapped to the *R. dominica* genome assembly. Read counts were normalized by Reads Per Kilobase of template per Million mapped reads (RPKM, [53]). 

### 2.5. Post Genome Analysis

#### 2.5.1. Gene Prediction and Annotation

Annotation of the *R. dominica* was generated by Dovetail Genomics. Repeat families found in the genome assemblies of *R. dominica* were identified *de novo* and classified using RepeatModeler (version 2.0.1, [54]). RepeatModeler depends on RECON (version 1.08) and RepeatScout (version 1.0.6) for the de novo identification of repeats within the genome. The custom repeat library obtained from RepeatModeler was used to discover, identify, and mask the repeats in the assembly file using RepeatMasker (version 4.1.0, [55]). Coding sequences from *Dendroctonus ponderosae*, *Hypothenemus hampei* and *Tribolium castaneum* were used to train the initial *ab initio* model for *R. dominica* using AUGUSTUS (version 2.5.5, [56]), with six rounds of prediction optimization. The same coding sequences also were used to train a separate *ab initio* model for *R. dominica* using SNAP (version 2006-07-28, [46]). Reads were mapped to the genome using STAR aligner (version 2.7, [57]), and intron hints were generated with the bam2hints tools within AUGUSTUS. MAKER and SNAP used the intron-exon boundary hints provided from aligned reads to predict genes in the repeat-masked reference genome. Swiss-Prot peptide sequences from the UniProt database were downloaded and used in conjunction with the protein sequences from the same training species to generate peptide evidence in MAKER [58]. Only genes that were predicted by both SNAP and AUGUSTUS were retained in the final gene sets. AED scores were generated for each of the predicted genes as part of the MAKER pipeline to assess the quality of the gene prediction. Genes were further characterized for putative function by performing a BLAST [59] search of the peptide sequences against the UniProt database. tRNA were predicted using the software tRNAscan-SE (version 2.05, [60]). Predicted genes were analyzed by BUSCO (v.2.0, [47]) using the lineage dataset insecta_odb9 (Creation date: 21 October 2016, number of species: 42, number of BUSCOs: 1658).

#### 2.5.2. Manual Annotation

The deduced amino-acid sequence of the 73 ABC transporter genes [61], along with the deduced amino-acid sequence of the *T. castaneum brown* ortholog [62] were used as query to identify *R. dominica* gene models in a *R. dominica* transcriptome assembly and the *R. dominica* genome. Forty-four ABC transporter genes were identified through blast analysis of gene prediction models, while an additional ABC transporter gene was identified during the analysis of the genome assembly.

Sequences corresponding to C1 cysteine peptidases were identified by tBLASTn [59] using default parameters. Cathepsins from *T. castaneum* (TcL_NP_001164001, TcLhom_XP_970773, TcB_XP_974298, TcBhom_XP_968689, TcTINAL_XP_008195382, TcO_XP_970512, TcF_XP_008195656, TcK_XP_001814509) were used as the protein query. Genes were further filtered manually and annotated within the web-based genome editing platform WebApollo [63] in the JBrowse Genome Browser [64] at i5k (https://i5k.nal.usda.gov/, accessed on 12 December 2020. The definition of the cathepsin L subfamily included analysis for the inhibitor domain I29 (pfam08246), and cathepsin B subfamily was analyzed for the propeptide domain (pfam08127) [65].

Sequences corresponding to proline-specific peptidases (PSPs) were identified with tBLASTn [59] using default parameters. PSPs from *Tribolium castaneum* (XP_008193477, XP_975053, XP_008193691, XP_971949, XP_015837624, XP_015836080, XP_015837563, XP_971305, XP_972807, XP_974698, EEZ97287, XP_008199099, XP_971576), *Homo sapiens* (NP_002717, NP_001926, NP_932064, NP_631898, NP_004451, NP_570629, NP_065919, NP_005031, NP_037511, NP_001161076, NP_003390, NP_071381, NP_000276), *Sipha flava* (XP_025423599.1), *Polistes dominula* (XP_015174578.1), *Onthophagus taurus* (XP_022921264.1), *Nicrophorus vespilloides* (XP_017775143.1) and *Blatella germanica* (PSN40454.1) were used as the protein query. Genes were further filtered manually and annotated as with cysteine peptidases.

We annotated chemoreceptors from the genome of *R*. *dominica* by iterative BLAST searches, initially using a database of ORs from ten diverse species of beetles [66] and GRs/IRs from the genomes of the Asian longhorned beetle (*Anoplophora glabripennis* (Motschulsky)), the emerald ash borer (*Agrilus planipennis* Fairmaire), the mountain pine beetle (*Dendroctonus ponderosae* Hopkins), and the flour beetle *T*. *castaneum* [35]. BLAST searches were repeated with each annotated model from *R*. *dominica* until no further hits were obtained. Predicted chemoreceptor sequences were annotated onto scaffolds manually using the software Geneious 9.1.8 (Biomatters Ltd., Auckland, NZ) and exons of ORs were named following [66]. Partial models were retained only if the sequence exceeded a threshold size (300 bp for ORs/GRs, 600 bp for IRs) and overlapped with all other partial models in a multiple sequence alignment (to prevent assigning unique names to multiple fragments of the same gene). We confirmed potential PSE in the assembly (deletion of coding sequence/splice sites, presence of stop codons, and/or frameshifts in the coding sequence) by mapping the raw genomic reads to the assembled model (Geneious 9.1.8 Mapper, default settings) and observing for the mutation in the reads. The PSE was promoted to functional status if the raw reads did not include the mutations observed in the assembled model. Alternative splicing has been described from ORs and GRs of beetles [35,66], and all models to date involve mutually exclusive N-terminal exons and shared C-terminal exons. Alternative splicing was proposed here for chemoreceptor models that lacked terminal exons if they were arrayed with other chemoreceptors that included the missing exons, and the intervening sequence had no unassembled regions. 

#### 2.5.3. Phylogenetic Analysis

Sequence alignments of *R. dominica* and *T. castaneum* cysteine peptidases were made with Clustal Omega [67], whereas alignments of *R. dominica*, *T. castaneum* and *H. sapiens* PSPs were with MUSCLE [68]. Cysteine peptidase phylogenetic trees were constructed in MEGA 7 [69] and PSPs in MEGA X [70] using Maximum Likelihood analysis with 500 bootstrapping iterations.

Chemoreceptors were aligned (MUSCLE; gap penalty −5; [68]) with members of their respective families annotated from genomes of a cucjiform species (*A*. *glabripennis*), a non-cucujiform species (*Nicrophorus vespilloides* Herbst or *A*. *planipennis*, depending on available gene sets), and a grain pest (*T*. *castaneum*). Only models > 450 bp were included from the comparison species to minimize problems with the alignment. Alignments were manually adjusted when necessary and trimmed with trimAL 1.2 [71] (similarity threshold 0, gap threshold 0.7, minimum 25% conserved positions). Phylogenies were generated using FastTree 2.1.11 at its default settings [72] and edited within Geneious, FigTree 1.4.4 [73], and Inkscape 1.0.2-2 (inkscape.org). ORs were rooted with Orco, GRs with sugar receptors, and IRs with IR8a/25a, as these are assumed to be ancestral lineages [33]. Chemoreceptors were initially numbered sequentially down the phylogeny, but when tandem arrays were present, numbering was rearranged to match the order within the array. Suffixes to gene names were assigned based on PSE status or in the case of incomplete models (NTE, CTE, INT; missing N-terminal, C-terminal, or internal exons, respectively).

#### 2.5.4. Expression Analysis

To analyze the expression of *R. dominica* cysteine and proline-specific peptidase transcripts, we mapped reads from the transcriptomes of different stages and tissues (whole adult, larvae gut, carcass, or head) to predicted cathepsin and PSP genes by the programs BWA [74] and SAMtools [75]. Expression values were normalized by RPKM. 

Antennal transcriptomes were not sequenced by the present genome project, but a few chemoreceptors of *R*. *dominica* were previously described from a transcriptome of antennal tissues from pooled sexes [43]. We re-mapped those data to our genomic OR models to confirm expression of these genes and identify other chemoreceptors that were highly expressed in the antennae. Expression was superficially assessed as absent or potential genomic contamination (0–5 reads), possible antennal expression (6–20 reads), or unambiguous expression with a tiled assembly (>20 reads).

For other digestive peptidases, *de novo* assembling of RNA-seq reads were performed with Trinity (v 2.8.5) and Trans-ABySS (v2.0.1) [76] RNA-seq reads were aligned to the genome with HISAT2 (v 2.1.0) [77] and map-based gene models were refined with StringTie (1.3.6) [78]. The transcript sequences from Trinity, Trans-ABySS and StringTie, and Swiss-Prot protein sequences were used as input to train the gene-prediction models in the MAKER pipeline (v2.3.10) [58] to generate *ab initio* gene models. MCuNovo [79] was used to select the best protein-coding genes modeled from those four programs. 

#### 2.5.5. Repeat Structure Analysis

Mobile elements were discovered and annotated using both *de novo* and homology-based tools. Transposons (TE) with terminal inverted repeats (TIR) were identified *de novo* with an inverted repeat finding tool IRF [80]. Different classes of repetitive sequences including TE were identified using RepeatModeler [54]. All TE candidate sequences were clustered with CD-HIT [81] and subsequently scanned for protein domains related to transposons using PFAM 31 [65] and CDD protein domains [82]. Only those TE-candidates with similarity to transposons were retained and merged with RepBase 2017 edition [83] as a custom library for RepeatMasker 4.0.7 [55]. RepeatMasker output was parsed with in house scripts filtering out hits with scores below 200, a threshold set by plotting scores for manually curated elements. Two datasets were created, namely (i) all TE with RepeatMasker scores better than 200 and (ii) TE additionally retaining similarity to typical TE coding regions.

For the detection and clustering of satellite DNAs, the *R. dominica* genome was imported to Tandem Repeats Database (TRDB, https://tandem.bu.edu/cgibin/trdb/trdb.exe, accessed on 3 August 2018) [84]. A search for tandem repeats (TRs) was performed using default parameters: alignment parameters 2, 7, and 7 (match, mismatch, indels) and 70% as the minimum alignment score. To preferentially focus our search on satellite DNA repeats (satDNA), arrays TRs were filtered using the following criteria: pattern size ≥ 100 and repeat copy number ≥ 2. Filtered arrays were processed with redundancy set at 50% overlap and PER (period to eliminate multiple reporting of repeats, i.e., same repeats found at different period sizes). The Clustering tool was used to group satDNA repeats into families that share at least 70% similarity, using the following conditions: cutoff value was set to 70; heuristical program DUST (to filter low complexity regions) and PAM (default values) options were included. Monomer sequences belonging to a specific cluster were downloaded from TRDB for further analysis. Multiple sequence alignments were performed to obtain consensus sequences for monomers. All subsequent analyses and sequence editing were done in Geneious^®^ 11.0.4.

## 3. Results

### 3.1. Sequencing and Assembly

We inbred a single mate pair of *R. dominica* for over 10 generations prior to initiation of the sequencing project. Previously, the size of the *R dominica* genome was estimated at 476 Mb and karyotype of 2n = 18, 8A + Xy_p_ [44,85]. We measured the genome size of each sex of the inbred strain by flow cytometry and found that males were 480.4 Mb (±1.2, 1C = 480 ± 1.7 Mb, *n* = 7), and females were 493.7 Mb (±1.2, 1C = 494 ± 1.7 Mb, *n* = 7).

Genomic DNA was extracted from male pupae from an inbred line and was sequenced with both short and long-read technologies. The long-read primary assembly resulted in 1861 contigs and an N_50_ of 0.87 Mb (Table 1). The primary assembly was then scaffolded with long-range data (Chicago) resulting in 948 scaffolds and an increased N_50_ of 7.32 Mb. The scaffolded primary assembly was again assembled with short reads for a hybrid assembly that further reduced the number of scaffolds to 336 and increased the N_50_ to 7.44 Mb. The hybrid assembly was rescaffolded with Hi-C data to achieve the final assembly of 479,170,650 bases, close to the predicted genome size of 480.4 Mb for males, in 139 scaffolds, with an N_50_ of 53.6 Mb. There were 10 larger scaffolds ranging in size from 14,104,112 to 82,855,609 bp. The BUSCO score [47] of the final assembly was 99.4% (98.9% single-copy, 0.5% duplicated, 0.2% fragmented, and only 0.4% missing reference gene sequences). 

### 3.2. Manual Annotation

The following sections are the results from manual annotation and in-depth analysis of select gene groups potentially associated with *R. dominica* ecology and behavior. Gene predictions are deposited at Ag Data Commons (doi.org/10.15482/USDA.ADC/1524749, accessed on 20 December 2020).

#### 3.2.1. Detoxification Genes

##### ATP Binding Cassette Transporters

*Rhyzopertha dominica* has evolved high levels of resistance to contact insecticides, such as chlorpyriphos-methyl, pirimiphos-methyl, malathion (organophosphates) [86], deltamethrin (pyrethroid) [87], and s-methoprene (juvenile hormone analog) [88]. The complete family of ATP binding cassette (ABC) transporters encoded in the *R. dominica* genome was manually annotated, as they have been implicated in resistance to several classes of insecticides through active transport of insecticides and their metabolized products across cellular membranes.

**Figure 2:**
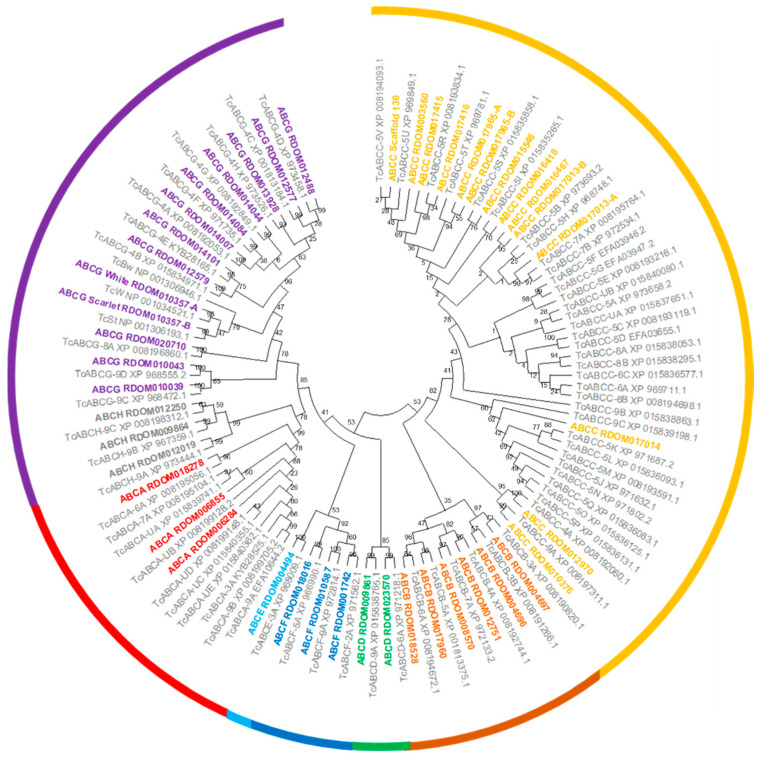
Phylogenetic tree of ABC transporters from *R. dominica* and *T. castaneum*. ABC transporters from *R. dominica* were named by combining the subfamily name with the name of the corresponding gene model. ABC transporters from *T. castaneum* were taken from previous studies [61] with the accession number provided for reference. The bootstrap values (%) are given at the nodes. ABC subfamilies (A–H) are indicated by different colors.

We identified and annotated a total of 45 ABC transporter genes in the *R. dominica* genome (Table 2). All *R. dominica* ABC transporters were grouped into eight subfamilies named ABCA to ABCH based on sequence homology (Figure 2). All ABCA and ABCC and two ABCB subfamily members encoded two nucleotide-binding domains and two transmembrane binding domains and thus are full-transporters. To date, only members of the ABCB and ABCC subfamilies have demonstrated involvement in insecticide resistance, while members of other subfamilies are presumed to be involved solely in the transport of endogenous substrates. Members of both the ABCB and ABCC subfamilies often are referred to as multidrug-resistance proteins (MRPs) since they can transport a variety of xenobiotic chemicals. 

A comparison among various beetle species and *D. melanogaster* indicated that *R. dominica* has one of the smallest repertoires of ABC transporter genes (Table 2), mainly due to far fewer ABCC genes (14) compared to other beetle species (which range from 24 to 37). The ABCC subfamily has undergone species-specific and in-tandem expansion in many beetles resulting in much higher gene counts [90]. However, *R. dominica* appears to lack the type of ABCC subfamily expansion seen in *T. castaneum* and *Aethina tumida* (Figure S2). The lack of gene expansion was particularly in contrast to that observed in the ABCC-5 subfamily in *T. castaneum* (Figure 2). Internally feeding larvae such as *R. dominica* are protected from externally applied contact insecticides until they emerge from the kernels as adults. Therefore, internal feeders such as *R. dominica* may have reduced selection for metabolic detoxification, thus explaining the lack of ABCC family expansion typically seen in other beetle species. 

Amongst members of subfamily G, the *white*, *scarlet*, and *brown* genes are the most studied in insects due to their conserved roles in eye pigmentation. The eye color gene *white* is frequently an initial target when testing the CRISPR/Cas9 system since the loss-of-function phenotype for the *white* eye-color gene is usually white eyes. Orthologs of *white* and *scarlet* were identified in the *R. dominica* genome and are prime targets for Cas9-based technologies. The *brown* ortholog was not found in the *R. dominica* transcriptome or genome, but this could be due to an insufficient degree of conservation, similar to *T. castaneum* [62]. Members of ABCE and ABCF are highly conserved among other subfamilies in number and sequence between insects and humans (Table 2) and do not function as transporters. Moreover, RNAi targeting ABCE and one of the ABCF genes in *T. castaneum* resulted in complete mortality, suggesting that the critical cellular roles of these genes also may be conserved [61].

##### Cytochrome P450s, UDP-Glucuronosyltransferases, Glutathione S-Transferases, and Carboxylesterases 

Families of genes encoding detoxification enzymes in the *R. dominica* genome included 84 gene models for cytochrome P450s (CYP450s), 46 UDP-glucuronosyltransferases (UGTs), 22 glutathione S-transferases (GSTs), and 15 carboxylesterases (CES, EC 3.1.1.1). Many of these genes were in tandem arrays similar to other sequenced beetle genomes, the largest of which was an array containing seven CYP450s on scaffold 2 from position 9413016–9561807 from the CYP9 family, six CYP450s on scaffold 137 from position 4120104–4224177 from the CYP6 family, and four CYP450s on scaffold 97 from position 30456026–30539921 also belonging to the CYP6 family. The most prominent CYP450 families were CYP6 (26 members), CYP9 (7 members) and CYP4 (6 members), which are also prominent in the genomes and transcriptomes of other beetles and whose members have been implicated in insecticide resistance [94,95]. 

CES have been linked previously to insecticide resistance and digestion/detoxification of recalcitrant dietary substrates (e.g., woody tissue; [96,97], and 15 copies were annotated in the genome of *R. dominica*. Four copies were found on scaffold 97 from 79510888–79557151 while the remainder were found as single copies or as a pair of tandemly duplicated genes. Interestingly, *R. dominica* appears to lack the large expansions of CES observed previously in the genomes of other beetle taxa, including *A. glabripennis* (>70 copies), *A. tumida* (>50 copies), *Agrilus planipennis* (>30 copies; Buprestidae), *Dendroctonus ponderosae* (>60 copies; Curculionidae), and even *T. castaenum* (>40 copies). We speculate that this may be similar to what was observed with the lack of expanded ABCC genes, a result of larvae feeding within the kernel and avoiding insecticide selection pressure. 

Gene models for UGTs were the most abundant phase II detoxification enzymes in the genome and were mostly found on scaffold 135, occurring as several small arrays of 2–3 genes across the scaffold and one large array of 14 genes from 34909176–35093838 (Figure S3). This 14 gene array represents a species-specific expansion of UGTs relative to other insects [90,98,99,100]. Similar small arrays of 2–3 UGT gene models could also be found on scaffolds 100 and 3. GSTs were prominent on scaffold 97, which contained an array of eight GSTs that represented a species-specific expansion. GSTs were also abundant on scaffold 100, but they were arranged as single copies and tandem duplications. 

Two gene models containing deltamethrin resistance pfam domains also were found in the *R. dominica* genome, both in distinct locations on scaffold 97. This domain was initially identified in the *prag01* gene, which is linked to deltamethrin resistance in *Culex pipiens pallens* and encodes a protein of 89 amino acids with unknown function [101]. The predicted proteins from *prag01*-like genes in *R. dominica* are both 96 amino acids in length and share <30% amino acid similarity with the resistance gene annotated in *C. pipiens pallens*. 

#### 3.2.2. Chemosensory Biology

We identified 311 chemoreceptor genes (123 ORs, 118 GRs, 70 IRs) in the genome of *R*. *dominica*, of which 45 (23, 9, 13, respectively) were predicted to be pseudogenes, and 24 (8, 5, 10) of those were pseudogenized by a single point mutation (Table S1). An additional nine pseudogenes were not supported by the raw reads and thus not considered to be functional. Almost all functional gene models were full-length, but 30 (20, 2, 8) partial models were included, of which most were missing only a small N- or C-terminal exon. 

##### Odorant Receptors

**Figure 3:**
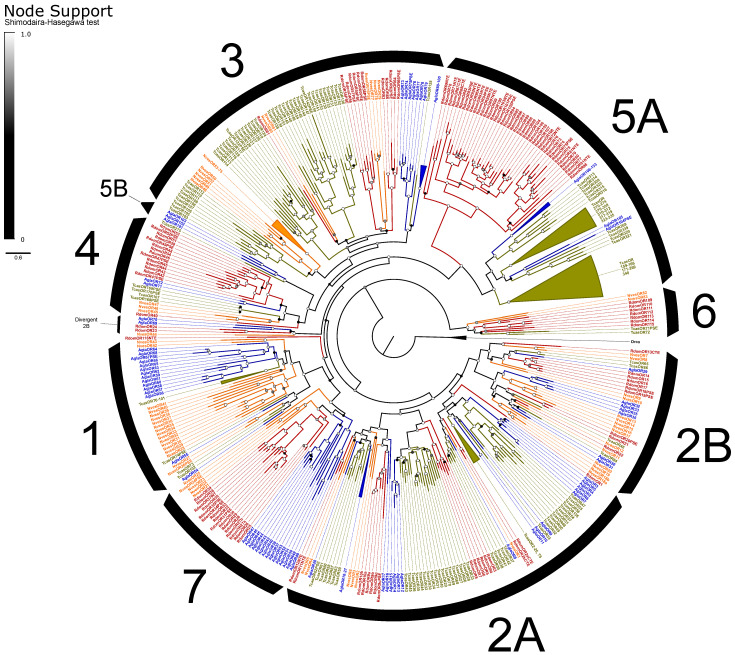
Phylogeny comparing odorant receptors (ORs) of *Rhyzopertha dominica* (red) to those of *Anoplophora glabripennis* (blue), *Tribolium castaneum* (yellow), and *Nicrophorus vespilloides* (orange), rooted with the conserved co-receptor Orco lineage. Colored circles at nodes represent Shimodaira-Hasegawa support values, shaded from black (0–0.5) to white (1.0). Black arcs describe the known subfamilies of coleopteran ORs (1–7). Some large lineage-specific expansions of ORs have been collapsed for clarity; identities of the removed genes are listed next to the collapsed sector.

The ORs included the expected single copy of the co-receptor Orco [102] and representatives from all recognized OR subfamilies in beetles except for Groups 1 and 5B (Figure 3). ORs exhibited an elevated rate of pseudogenization, with 19% of models considered pseudogenes compared to an average of 10% of ORs across other annotated beetle genomes [66]. Alternative splicing of ORs is uncommon in insects, and we identified only four loci that potentially exhibit alternative splicing, including three functional isoforms of RdomOR9 that shared only the terminal exons D and E. The remaining alternative splices consisted of paired isoforms, one pair of which (RdomOR10) also shared D and E exons, and two pairs (RdomOR2 and RdomOR27) that shared all but the first fragments of exon A. However, no alternative splices could be confirmed with transcriptome data.

ORs of *R*. *dominica* sorted into the expected lineage-specific expansions among the larger OR subfamilies [66], though Group 2A appears paraphyletic, and a divergent group of 2B genes (including RdomOR23–24) are misplaced due to the limited gene set used to construct these phylogenies (Figure 3). The largest expansion (RdomOR67–108) emerged in Group 5A, a prolific subfamily previously known only from cucujiform beetles [34]. No members of Group 5A have been functionally characterized, but the subfamily includes over 150 members in *T*. *castaneum*, where their expression is strongly associated with mouthparts [103]. *R*. *dominica* also presented a large radiation of ORs in Group 4 (RdomOR40–54), which was previously notable as the only coleopteran OR subfamily lacking such radiations.

Six conserved lineages of coleopteran ORs are presently recognized in beetles and are placed in Groups 1, 2A, and 2B [66]. *R*. *dominica* included four sets of genes (RdomOR9–10; RdomOR14–19; RdomOR22; RdomOR23–24) homologous to the four conserved lineages described from Groups 2A and 2B (Figure 3). Most notably, RdomOR14–19 are members of a lineage that also includes the pheromone receptor McarOR20 from the cerambycid *Megacyllene caryae* (Gahan) [36,66].

Six ORs were previously described from the transcriptome of antennal tissues [43], all of which were partial models except for Orco. We extended those models to full-length, reclassified one OR as a fragment of RdomGR3, and combined two OR models that were fragments of RdomOR1. This resulted in a total of four ORs annotated from the transcriptome: Rdom\Orco, OR1, OR10a, and OR35PSE. Re-mapping the antennal transcriptome data to our genomic models supported the paucity of ORs recovered by the previous publication [43], with only Orco, OR1, and one additional model, OR17, represented robustly, and most ORs unrepresented. A correspondence of gene names between the previous annotation and the present study is in Table S2.

The pseudogene OR35PSE was highly expressed in the published antennal transcriptome [43] and also was expressed in other tissues (head, carcass, adult body) measured by the genome project. The read mapping initiated downstream (nt515–520) of the predicted start codon and suggests a new initiation site at nt685 that skips the nonsense mutation. If translated, this abbreviated protein would include only three of the seven predicted transmembrane domains, and presumably no longer has olfactory function.

##### Gustatory and Ionotropic Receptors

**Figure 4:**
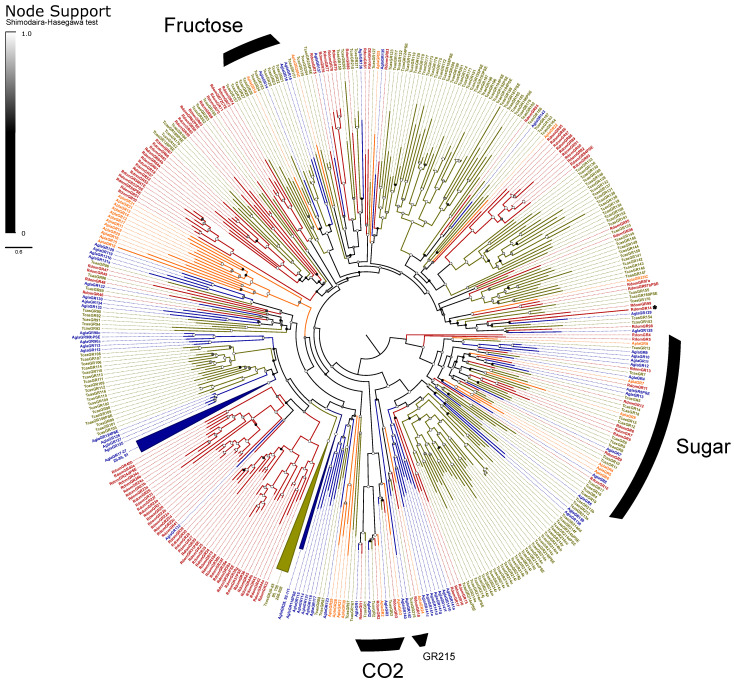
Phylogeny comparing gustatory receptors (GRs) of *Rhyzopertha dominica* (red) to those of *Anoplophora glabripennis* (blue), *Tribolium castaneum* (yellow), and *Agrilus planipennis* (orange), rooted with the conserved lineage of sugar-sensitive receptors. Colored circles at nodes represent Shimodaira–Hasegawa support values, shaded from black (0–0.5) to white (1.0). Black arcs describe the known monophyletic subfamilies of coleopteran GRs, and remaining unlabeled GRs are assumed to be bitter sensitive. Three large lineage-specific radiations of GRs have been collapsed for clarity; identities of the removed genes are listed next to the collapsed sector. An asterisk denotes RdomGR14, which is likely to be a divergent member of the fructose GRs (see text).

The GRs are classified into three monophyletic lineages (gaseous carbon dioxide, sugar, and fructose receptors) and a paraphyletic assembly believed to detect bitter tastants [33]. We recovered the expected orthologs to all three CO2 receptors (*GR1–3*) and a radiation of ten sugar receptors (*GR4–13*; Figure 4). We assumed that the single gene *GR14* is a highly divergent member of the fructose-sensitive GRs based on BLAST similarity scores to the ortholog *Gr43a* of *D. melanogaster* Meigen, but we could not recover this placement in our phylogeny. The remaining 85 genes, including all pseudogenes, were considered as bitter GRs and included eight alternatively spliced loci for a total of 118 isoforms. All alternative splices follow the canonical pattern of mutually-exclusive N-terminal exons spliced with 1–2 shared short C-terminal exons [33]. The bitter GRs also include a single representative (*RdomGR18*) of the recently described “*GR215* clade”, a lineage of simple orthologs that appear to persist throughout the coleopteran tree of life [35].

**Figure 5:**
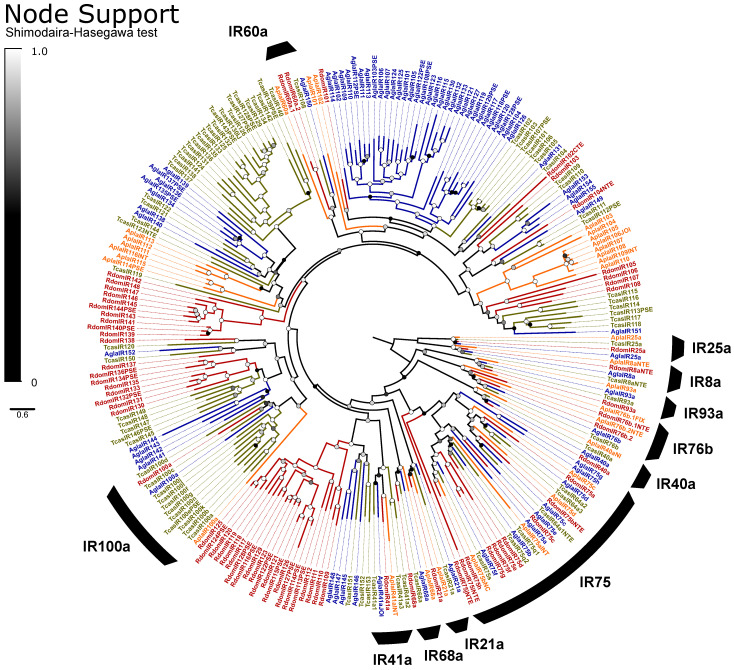
Phylogeny comparing ionotropic receptors (IRs) of *Rhyzopertha dominica* (red) to those of *Anoplophora glabripennis* (blue), *Tribolium castaneum* (yellow), and *Agrilus planipennis* (orange), rooted with the conserved co-receptors IR8a and IR25a. Colored circles at nodes represent Shimodaira–Hasegawa support values, shaded from black (0–0.5) to white (1.0). Black arcs describe the coleopteran lineages of conserved antennal IRs, named by their ortholog in *Drosophila melanogaster* (bottom right). Unlabeled IRs and the conserved monophyletic lineages IR100a (left) and IR60a (top) are classified as divergent IRs.

IRs are broadly separated into the “antennal IRs”, which are preserved as orthologs among many orders of insects, and “divergent IRs” that radiate in lineage-specific expansions similar to the ORs and bitter GRs [104]. Antennal IRs cover a range of olfactory functions, including sensing amines, acids, and aldehydes, as well as temperature and humidity, while divergent IRs are associated with gustatory function (summarized in [105,106]. Due to their high sequence similarity, the names and presumed function of coleopteran antennal IRs are taken from their orthologs in *D*. *melanogaster*, with the radiations of divergent IRs numbered sequentially beginning at *IR100* (except for the conserved lineages of *IR60a* and *IR100a*). Ten lineages of the conserved antennal IRs are sustained in beetles [66], and we annotated a single ortholog of each in *R*. *dominica*, plus two copies of *IR76b* and ten paralogs in the *IR75* clade (Figure 5). The divergent IRs included 50 genes, including all annotated pseudogenes, two members of the *IR60a* clade, and one member of the *IR100a* clade. 

Partial models of eight IRs were previously annotated from the antennal transcriptome study [43]. We extended these models to full-length, revealing that three were separate fragments of *IR25a* and two were fragments of *IR93a*. This resulted in a total of five IRs noted from antennal tissues, all of which were antennal IRs, including *41a*, *68a*, *25a*, *76b.2*, and *93a*. IR41a is associated with the olfactory detection of amines, while IR68a detects humidity, and the latter three IRs are required co-receptors in those same processes [107,108]. Re-mapping the reads from the previous study [43] to genomic IRs and GRs again supported the limited expression of chemoreceptors, with reads mapping almost exclusively to the above-named genes, as well as *IR8a* and the CO2 receptor *RdomGR3*. However, we also observed support for at least some expression of isoforms of *RdomGR24*, scattered bitter and sugar GRs, and members of the *IR75* clade (Table S1).

Our data also provide a candidate pheromone-sensitive OR in *R*. *dominica*. *RdomOR17* is a member of a lineage of conserved ORs that dates to the earliest extant beetles (see “2B.ii”, [66]) and which includes a pheromone receptor of the cerambycid beetle *M*. *caryae* (McarOR3, [36]). *RdomOR17* was also one of only four ORs that were highly expressed in a transcriptome of antennal tissues of combined male and female *R*. *dominica* although it was overlooked in that study [43]. These data are consistent with an OR that is sensitive to an aggregation pheromone, and we recommend OR17 as a strong candidate for future functional characterization.

#### 3.2.3. Digestive Enzymes

Digestive enzymes are important in adaptation to food sources, and the following are annotation projects that were focused on different hydrolases that may contribute to the ability of *R. dominica* to successfully digest cereal grains.

##### Carbohydrases

Glycoside hydrolases (GHs) are the major digestive enzymes that facilitate breakdown of complex carbohydrates ingested by insects. *R. dominica* feeds on grains that have a high content of starch, so we investigated the genome for expansion of α-amylases, maltases, and GHs relative to the genomes of other beetles that break down carbohydrate and di-, tri-, and oligosaccharides released from starch molecules. Because *R. dominica* reportedly bores into wood in the field, we also looked for genes that coded for Plant Cell Wall Degrading Enzymes (PCWDEs). Genes encoding GH 5, 43, 44, 45, and 48 enzymes have been implicated as PCWDEs in wood-feeding beetles, but members of GH family 9 encoded by insects also have been implicated in wood digestion in other orders (reviewed in [109]). 

**Figure 6:**
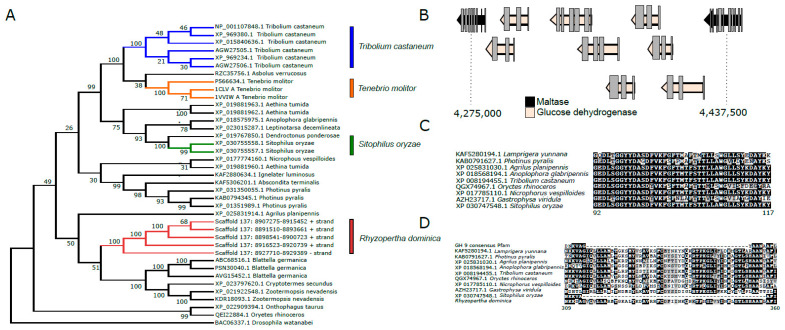
Carbohydrases present in the genome of *Rhyzopertha dominica*. (**A**). Neighbor-joining tree of α-amylases in *R. dominica* and in the genomes of other beetles. Sequences with >40% similarity to those annotated in *R. dominica* were downloaded from NCBI for phylogenetic analysis. Proteins were aligned with MUSCLE in MEGA X, and a bootstrap consensus tree was constructed using 500 bootstrap pseudoreplicates. Evolutionary distances were computed using the JTT distance matrix, and rate variation was modeled with a gamma distribution (shape parameter = 1). Accession numbers of proteins used in the tree are presented, and numbers on nodes indicate bootstrap support. (**B**). An expansion of glucose dehydrogenases on scaffold 4 in *R. dominica*. An array of 10 gene models for glucose dehydrogenases was found between two maltase genes. Although similar arrays of glucose dehydrogenases can be found in the genomes of other beetles, none were flanked by maltase gene models. Whether adjacent models can be used to infer putative gene function has not yet been widely investigated in insect genomes. (**C**). A deletion in a gene model coding for a GH 9 enzyme in *R. dominica*. One gene coding for a putative GH 9 enzyme was identified in the *R. dominica* genome. However, an ~25-amino-acid deletion was noted relative to other beetle endoglucanses, suggesting it may have a different function. (**D**). An insertion in a gene model coding for a putative GH 9 enzyme in all beetles genomes. A large ~50-amino-acid insertion was observed in all sequenced beetles relative to the consensus Pfam sequence. Notably, although many GH 9 gene models code for endoglucanses, many beetle GH 9 enzymes are thought to have different substrate preferences. This large insertion suggestions that these enzymes may no longer act on cellulose and may have different functions even in wood-feeding insects, such as *Anoplophora glabripennis*.

Analysis of the *R. dominica* genome revealed that copy number expansions of genes coding for α-amylases (EC 3.2.1.1) are common among stored product beetles. An array of six genes encoding α-amylases were identified on scaffold 137 (Figure 6A). *T. castaneum* has a similar array of genes on linkage group 2 [110]. An expansion of three genes coding for α-amylases also was found in the genome of *Tenebrio molitor* (GCA_014282415.2); however, the structure of these genes is unknown since it was from an unannotated genome. We hypothesize that expansions of α-amylase gene copies in stored-product beetles from divergent taxonomic lineages (Bostrichidae and Tenebrionidae) represent a convergent adaptation for feeding on amylaceous commodities. Notably, copy numbers of genes encoding α-amylases tend to be more numerous in dipteran genomes compared to coleopterans [111,112], as they largely consist of species occupying ecological niches as scavengers or phytophages.

The *R. dominica* genome contained multiple copies of genes encoding maltase (EC 3.2.1.20) that likely degrade maltose and α-1,4-linked glucose oligosaccharides released by α-amylases during digestion. Copies of maltase genes were found on scaffolds 3, 4, and 135. Maltase genes on scaffold 4 were a region containing multiple copies of glucose dehydrogenase genes, an enzyme that catalyzes the conversion of glucose to D-glucono-1,5-lactone, which can enter the pentose phosphate pathway for synthesis of NADPH and ribose 5-phosphate (Figure 6B). 

Other prominent GH families found in the *R. dominica* genome were chitinases (EC 3.2.1.14) from GH 18 and 20, α-glucosidases (EC 3.2.1.20) from GH 31, and α-mannosidases (EC 3.2.1.24) from GH 38 and 47, all of which are commonly present as multiple copies in other beetle genomes [90,98,113]. The precise metabolic or physiological functions of these gene families are not well-characterized in most cases, but their presence in multiple beetle genomes from disparate lineages suggests they are likely involved in conserved metabolic functions rather than coding for niche-specific digestive enzymes. *GH 1* genes in *R. dominica* (15) were similar in copy number to those detected in the *T. castaneum* and *A. tumida* (Nitidulidae) genomes, which code for 13 and 8 GH Family 1 enzymes, respectively, and are thought to have roles in digesting di- and tri-oligosaccharides. Interestingly, the first exons of a *GH 1* pseudogene on scaffold 98 shares 60% amino acid similarity with GH 1 enzymes derived from plants, several of which are annotated as 4-hydroxy-7-methoxy-3-oxo-3,4-dihydro-2H-1,4-benzoxazin-2-yl glucoside beta-D-glucosidase (DIMBOA glucosidase). DIMBOA is a powerful benzoxazinoid toxin produced by maize and wheat. Although prominent in young seedlings to protect against herbivory during vegetative growth [114], DIMBOA is also stored in seeds as DIMBOA-glucoside [115]. However, this gene is disrupted by a transposase and contains several frameshift mutations in *R. dominica*. The *R. dominica GH 1* pseudogene is predicted to be inactive. GH in insect salivary secretions or plant-derived GH stored in the chloroplast can remove glucose and activate DIMBOA. Many insect species have evolved mechanisms to protect them against DIMBOA and other benzoxazinoids. For example, *D. virgifera virgifera* has evolved the ability to sequester and re-glycosylate several metabolites of DIMBOA to less-toxic compounds [116]. The pseudogenization of this GH in *R. dominica* may reduce the effective hydrolysis of DIMBOA-glucoside. Similar pseudogenes have not been found in the genomes of other sequenced beetle species. 

Cellulase or other PCWDEs genes were not identified in the genome, contrary to documented reports of wood-feeding in field populations of *R. dominica*. A single copy of a gene encoding GH 9 endoglucanase was found on scaffold 137 downstream from the array of genes encoding α-amylases (Figure 6C). However, *GH 9* orthologs exist in the genomes of most sequenced insects (Figure 6D). In many cases, including *R. dominica*, the catalytic domain appears to be non-functional, and thus, it is currently hypothesized that these genes do not have cellulolytic capacities but instead code for other carbohydryases. GH 9 genes are not thought to be involved in cellulose digestion in phytophagous coleopteran species [117] although these genes may act as cellulases in Blattodea (termites) and Phasmatodea (stick insects) and other insect orders [118,119,120]. No other genes coding for putative plant cell wall degrading enzymes were identified in the *R. dominica* genome (e.g., GH 5, 43, 44, 45, 48). 

##### Peptidases

Insect digestive peptidases are among the most abundant and essential enzymes necessary for metabolism. Based on their catalytic mechanisms and a specific residue in the active site, peptidases are classified into five main subclasses: cysteine (cysteine proteases), aspartate (aspartate proteases), metal ion (metalloproteases), threonine (threonine proteases), and serine (serine proteases) [121]. 

##### Proline-Specific Peptidases (PSPs)

Proline-specific peptidases (PSPs, EC 3.4.x.x) hydrolyze bonds formed by a proline residue, which is resistant to proteolysis by most peptidases with broad substrate specificity [122,123]. Most PSPs are exopeptidases, cleaving amino acids from both ends of a polypeptide chain: dipeptidylpeptidases (DPP, EC 3.4.14.x) DPP2 (EC 3.4.14.2), DPP4 (EC 3.4.14.5), DPP8, DPP9, fibroblast activation protein (FAP, EC 3.4.21.B28), prolylcarboxypeptidase (PRCP, EC 3.4.16.2), aminopeptidase P (APP, E.C. 3.4.11.9) APP1, APP2, APP3, prolidase (EC.3.4.13.9); and only one endopeptidase—prolyloligopeptidase (POP, EC 3.4.21.26). There are also two inactive homologs of DPP4-like proteins—DPP6 and DPP10. 

The diet of *R. dominica* includes wheat with the storage proteins gliadins that have up to 30% proline residues [124,125]. We expected that *R. dominica*, similar to *T. castaneum*, would express enzymes that effectively digest proline-rich proteins, and therefore we annotated PSPs in the genome sequence of *R. dominica.*

Thirteen PSP sequences were annotated in the genome of *R. dominica* (Table 3). According to MEROPS classification [121], nine sequences were serine peptidases of the S9 family, namely POP (*RDOM022815*), DPP4 (*RDOM016604*, *RDOM017825*), DPP9 (*RDOM002099*), and DPP10 (*RDOM016697*, *RDOM007500*, *RDOM007053*), and the S28 family, namely PRCP (*RDOM004644*, *RDOM021888*). Four were metallopeptidases of the M24 family, namely APP1 (*RDOM016283*, *RDOM01413*), APP3 (*RDOM000819*), and prolidase (*RDOM021565*). *R. dominica* lacked DPP2, DPP6, DPP8 (which is similar to DPP9), FAP, and APP2 compared to human PSPs [122,123]. However, there were three isoforms of DPP10 and two DPP4 and two PRCP sequences, similar to *T. castaneum* [110]. 

**Figure 7:**
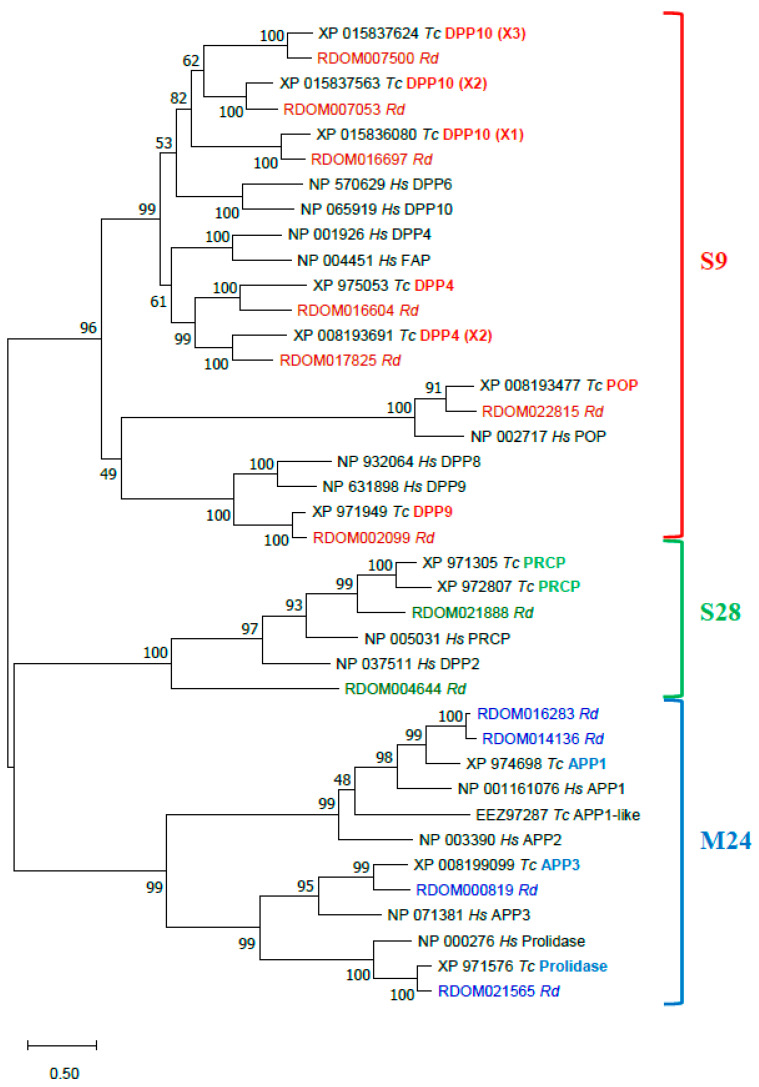
Phylogenetic analysis of *R. dominica, T. castaneum,* and *H. sapiens* PSPs. Maximum Likelihood trees with 500 iterations of bootstrapping were used; bootstrapping values are represented as a percentage (0–100) by each branch. Evolutionary distances are represented by branch’s length. Serine PSPs of S9 family are colored with red, S28 with green, and metallopeptidases of M24 with blue. Types of peptidases (on the example of *T. castaneum*) are indicated in bold with corresponding color; X1, X2, X3—mean isoforms of one type. *R. dominica* sequences are colored according to the family.

The annotated *R. dominica* PSP proteins were compared to PSPs of *T. castaneum* and human in a phylogenetic analysis of proteins sequences (Figure 7). All *R. dominica* PSPs clustered with the corresponding conserved proteins from *T. castaneum* and *H. sapiens*. All presumably active serine PSPs had the typical Ser-Asp-His catalytic triad (Table 3). Three sequences of DPP10 homologs of S9 serine PSPs were found in *R. dominica*. Two (RDOM016697 and RDOM007500) had a Gly residue instead of Ser in the active site, and the third (RDOM007053) had a Ser residue like other active members. Orthologs of *R. dominica* DPP10 also were found in *T. castaneum* (Figure 7); *T. castaneum* sequences XP_015836080 (isoform X1) and XP_015837624 (isoform X3) had a Gly instead of Ser in the active site, and XP_015837563 (isoform X2) had no substitutions in the active site. This feature may be typical for only insects. Metallopeptidases of the M24 family had conserved residues in two Mn^2+^-binding sites.

Most PSP genes from *R. dominica* demonstrated similar levels of RNA expression in the larval gut, carcass and head; however, some were expressed predominantly in the gut (DPP4 *RDOM016604*, prolidase *RDOM021565*), carcass (PRCP *RDOM004644*), head (POP *RDOM022815*, DPP10 *RDOM016697*), and both gut and carcass (APP3 *RDOM000819*) (Table 3). Three PSP sequences of *R. dominica* contained a signal peptide, which indicates they are secreted enzymes. Two (*RDOM004644* and *RDOM021888*) were predicted to be PRCP-like peptidases, which is consistent with biochemical studies that have shown lysosomal localization for PRCP of *T. molitor* [126], swine [127], and human [128,129]. The third (*RDOM016604*) was one of two predicted DPP4 peptidases in *T. molitor* [130]. Interestingly, all mammalian PSPs of the S9 family do not have signal peptide contrary to DPP4 of *T. castaneum* (XP_975053) and *T. molitor* DPP4 (QAY29072) [130]; however, *R. dominica* DPP4 (*RDOM016604*) had a signal peptide and was expressed predominantly in the gut, supporting the possible role in digestion.

##### Serine Peptidases

Serine endopeptidases (EC 3.4.21) are an important part of digestion and involved in development and immunity in *R. dominica*. Serine peptidases belong to the largest peptidase clan, PA, with over one third of all known proteolytic enzymes [131]. Serine peptidases are produced by the midgut epithelial cells and secreted into the lumen and are responsible for 95% of protein digestion in lepidopteran insects [132]. Protein digestion was attributed to a complex of serine proteinases present in the midgut of *R. dominica* larvae [133]. The full cDNA sequences for trypsin and chymotrypsin were isolated from the larval midgut [134,135], but otherwise little is known about peptidases in *R. dominica.*

An in-depth study of all the functions of serine peptidases in *R. dominica* is beyond the scope of this general overview of the genome and will be expanded in a companion article. Here we limit discussion to the role(s) of serine peptidases in digestion. A previous study on digestion-related proteins in *Manduca sexta* [136] was used as a guide to search for homologs in *R. dominica*. There were in total 125 serine peptidase-related proteins in the S1A family. Putative digestive enzymes included 32 that encoded single-domain serine peptidases (SPs) and two that encoded non-catalytic SP homologs (SPHs) (Table S3). Digestive serine peptidases ranged from 254 to 469 amino acids and from 26.5 to 50.3 kDa in molecular mass, with a wide pI range of 3.2 to 8.3, which suggests these enzymes are sensitive to pH regulation of activity. The average mRNA levels of our predicted serine peptidase genes in the gut, head, and carcass of *R. dominica* were 121:1.2:1.0, suggestive of a major role in the digestion of dietary proteins. We estimated that 27% of the *R. dominica* SPs and SPHs are probably related to digestion, similar to five other holometabolous insects, which employ 24–30% of their SP-like genes in digestion [137]. The data suggest that *R. dominica* peptidase genes are expanding rapidly by gene duplication and divergence. Most peptidase genes were present in clusters on scaffolds, likely formed by lineage specific gene duplication. The largest cluster of serine peptidase genes consisted of 10 genes on scaffold 137. 

##### Metalloexopeptidases

Ten zinc carboxypeptidases (EC 3.4.16-3.4.18) and 10 aminopeptidases (EC 3.4.11) were expressed in the gut at much higher levels than in head or carcass, indicating that metalloexopeptidases likely actively participate in protein hydrolysis as well (Table S3). 

#### 3.2.4. Other Genes of Interest

##### Aquaporins

Aquaporins (AQPs) belong to the major intrinsic protein family that function in the transport of water and other small solutes across biological membranes and play important roles in osmoregulation, water retention/excretion, stress (desiccation/thermal/oxidative) tolerance, digestion, and reproduction [138,139]. AQPs generally consist of six transmembrane domains connected by five intra- and extracellular loops, with intracellular amino and carboxyl terminal ends [138]. Prototypical AQP water transporters contain two tandem repeats of Asn-Pro-Ala motifs that regulate the single-file conductance of water, while selectively restricting the flow of protons and other cations [140]. Solute selectivity is also regulated by a second constriction formed by an aromatic residue and an Arg (known as the ar/R motif) [141,142,143,144,145].

To date, eukaryotic AQPs are classified into four primary grades, including the classical AQPs, aquaglyceroporins (Glps), unorthodox AQPs, and Aqp8-type aquaammoniaporins [140]. Insects possess classical, unorthodox, and Glps, with classical AQPs being highly abundant in insects. Glycerol transport is associated with the Glps in many eukaryotes and has seemingly been replaced in holometabolan insects by the evolution of the entomoglyceroporins (Eglps) [146]. Eglps specifically arose from other classical AQPs through the substitution of the conserved His in the ar/R selectivity filter to uncharged residues such as Ala and Ser. Unorthodox aquaporin 12-like (AQP12L) AQPs also exist in insects [146,147]. AQP12Ls are related to the vertebrate AQP12 channels and are unique in that they may have intracellular localization, do not have conserved NPA motifs, and have substitution of the Arg within the ar/R selectivity filter [148,149]. Aqp8-type aquaammoniaporins have yet to be found and appear to be absent in arthropods [140].

**Figure 8:**
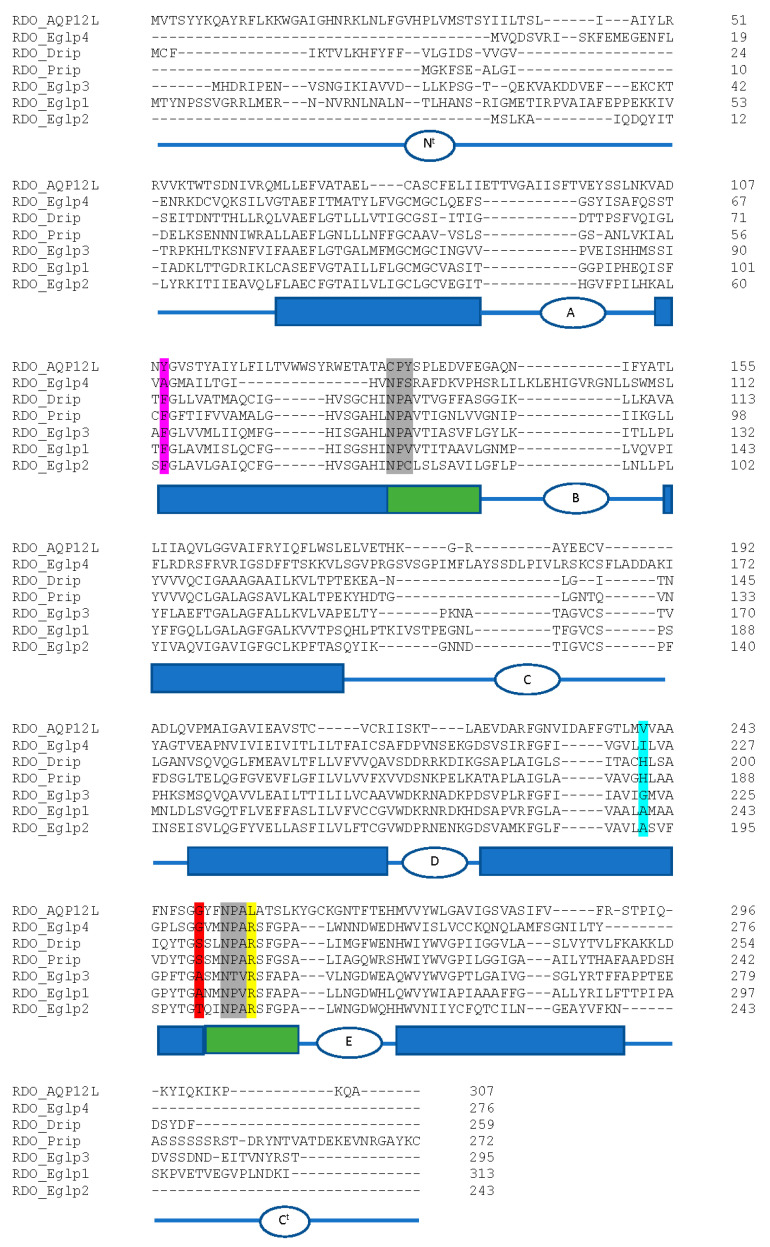
Amino acid sequence alignment of *R. dominica* aquaporins. The deduced amino acids of the *R. dominica* AQPs (excluding big brain) were aligned using CLUSTALW. NPA motifs are highlighted in grey. Residues that correspond to the ar/R selectivity site from *Homo sapiens* AQP1 (F56, H180, C189, R195; EAL24446.1) are highlighted in magenta, cyan, red, and yellow, respectively. Schematic below sequence alignment show domain structure based on predictions from the TMHMM Server v. 2.0. Rectangular boxes denote the position of the transmembrane helices (blue boxes) and the hemi-helices with the NPA motifs (green boxes). Intra- and extracellular loops A-E are denoted as ovals with letters. N^t^, amino-terminal domain, C^t^, carboxyl-terminal domain.

We identified a total of eight aquaporin (AQP) genes in the *R. dominica* genome (Table 4), which is similar to the total number found in most other insect species [150,151,152,153]. Phylogenetic analysis of the translated protein sequences from each of the eight genes revealed representatives belonging to the *Drosophila* integral protein (Drip), *Pyrocoelia rufa* integral protein (Prip), big brain (Bib) proteins, Eglps, and unorthodox AQP12L families (Figure S4). Both canonical NPA sites were conserved within RDO_Drip, RDO_Prip, and RDO_Eglp3 (Figure 8). All other *R. dominica* AQPs had only one or no conserved NPA sites. 

##### RdCad1, a Putative Receptor of Insecticidal Cry Toxins

Atypical insect BtR1-like cadherins are proposed to be key determinants in establishing the structural and functional integrity of the alimentary channel throughout the larval growth and molting phases [154]. BtR1 cadherins also are important elements of the *Bacillus thuringiensis* (Bt) insecticidal Cry toxin binding interface, where binding to Cry toxins leads to severe disruption of the midgut epithelial tissue in susceptible insects [154,155]. These cadherins only share similar topology with vertebrate “classical cadherins” in that they are type-1 transmembrane proteins composed of an extracellular domain with up to twelve cadherin repeats or ectodomain modules (ECs, IPR002126), most containing Ca^2+^-binding sites, a hydrophobic single-span transmembrane domain, and a cytoplasmic region (CYT). Cadherin repeat domains are followed by the loosely conserved membrane-proximal extracellular domain (MPED) located immediately adjacent to the TM domain [156]. 

Cry toxin-binding regions (TBRs) in *M. sexta* BtR1 were localized within EC7 (TBR1), EC11 (TBR2), and EC12 (TBR3) [157,158,159]. These conserved sequences have been used to identify potential TBRs of other insect midgut BtR1-like cadherins, including those that are proposed to interact with coleopteran-specific Cry3Aa/3Bb toxins [160,161,162]. 

**Figure 9:**
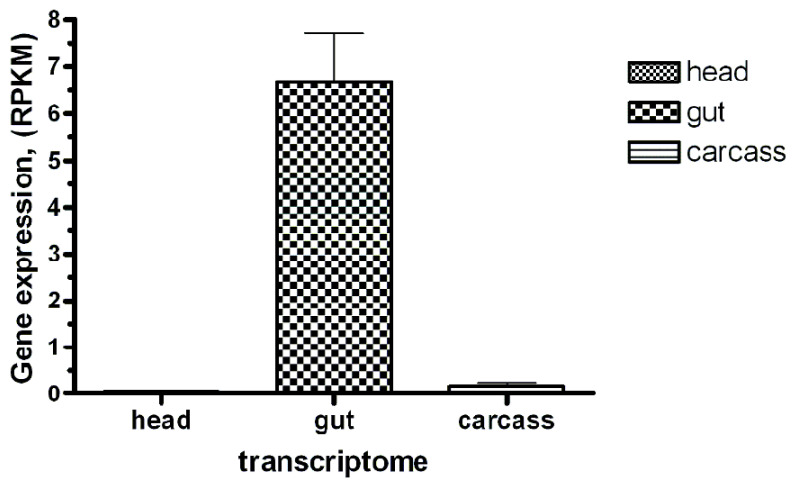
The Bt-R1-like cadherin-specific transcript abundance in the *R. dominica* larvae transcriptomes (head, gut, and carcass). Sequence reads obtained after Ion Torrent sequencing were mapped to the open reading frame of the putative cadherin identified by similarity with several previously studied “reference” insect Bt toxin receptor cadherins. The mapped read counts were normalized by Reads Per Kilobase of template per Million mapped reads (RPKM, ±SE, *n* = 3).

**Figure 10:**
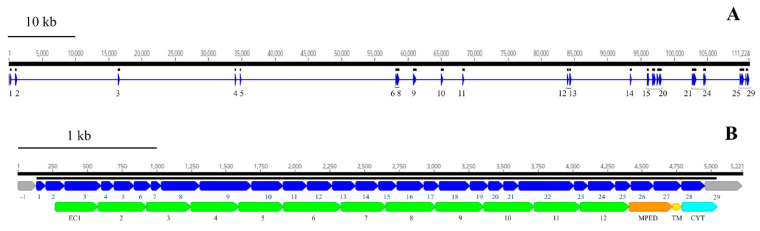
Genomic and domain organization of the putative Bt-R1-like cadherin of *R. dominica*. (**A**) Schematic representation of organization of the RdCad1 cadherin gene. Thin, horizontal blue line below the black solid scale indicates the genomic sequence, with the individual exons shown as blue triangles. (**B**) Schematic representation of the exons (blue rectangles) and their encoded RdCad1 functional domains EC1–12, MPED, TM, and CYT (lower). The above black, solid lines correspond to the transcript coding region and the scale, respectively. The first 5’-UTR (−1) and the last 3’ (29) exons are depicted in grey, as their untranslated sequence regions were not experimentally confirmed.

We identified and annotated a single *BtR1*-like cadherin gene (*RdCad1*) on Scaffold 97 of the *R. dominica* genome (Figure S5), and the predicted *RdCad1* transcript was highly expressed in midgut tissue (Figure 9). The genomic sequence of this midgut-specific cadherin was approximately 111.2 kb and was interspersed with 29 introns (Figure 10). The first intron of the sequence was in the 5’-UTR region, 3 bp upstream of the ATG start codon, whereas the final cadherin protein translation site was encoded by 29 exons. The exon length varied from 64 bp for the smallest exon 1, to 399 bp for exon 22, with an average length of 169 (Table S4). The total coding exons of *RdCad1* were 4.9 kb, close to the 5.1 kb reported for other coleopteran cadherins and even more distant lepidopteran cadherins. The introns of *RdCad1* were more variable in length than the exons, ranging from a 51 bp intron 7 to intron 6 spanning 23,227 bp. The intronic sequences of beetle BtR1-like cadherin genes are generally larger than those of related lepidopteran genes [163]. Longer introns may be due to the accumulation of numerous transposable elements (Table S4) that intersperse many arthropod genomes [164] as discussed in Section 3.3.1.

TBRs from *M. sexta* BtR1 cadherin were used to identify RdCad1 TBR1 (amino acid 810–851), TBR2 (1268–1312), and TBR3 (1358–1389) as potential regions of interest (Figures S5 and S6). TBR1 in EC7 contained the consensus signature ITIYIxDxNN which is shared in many BtR1 cadherins (Figure S6). Although the importance of this TBR in Bt toxicity remains somewhat controversial [158,165], the TBR1 motif KV/I (aa: 25–26, consensus) also is conserved among many Cry susceptible lepidopterans, as well as in BtR1 from the Colorado potato beetle, *Leptinotarsa decemlineata*. This species is susceptible to Cry3A and formulations thereof but is notorious for resistance development [166]. TBR2 mapped to the distal part of EC11 and shares the hydrophobic motif SxLTVTV. The basic R31 residue (consensus) was conserved among the cadherins of only Cry toxin susceptible lepidopterans and chrysomelid beetles (CPBCad and DvCad) but was not found among the less susceptible tenebrionids or *R. dominica*. TBR3 in EC12 adjacent to the MPED domain did not contain the three lysine residues found in TBR3 of *T. castaneum* cadherin, proposed as potentially disruptive to toxin binding and contributing to reduced sensitivity to Cry3A Bt toxins [161]. Since RdCad1 did not contain similarly charged residues in TBR3, this may explain the moderate toxicity of Cry3Aa in *R. dominica* [167].

Sequence comparison of RdCad1 with insect cadherin orthologs revealed 30–34% identity with lepidopteran and tenebrionid cadherins and 28% identity with chrysomelid cadherins (Figure S7). Phylogenetic analysis suggests that the first Bostriciformia BtR1-related cadherin represents a distinct clade of beetle cadherins. 

##### Cysteine Peptidases

Cysteine peptidases (EC 3.4.3) from the C1 family (MEROPS classification, [121]) are the major lysosomal proteins in all eukaryotic organisms and, in addition to protein turnover, participate in many biological processes studied in mammals [168]. Cysteine cathepsins in a limited number of insect groups (mostly from Coleoptera, Hemiptera, and Ixodida) evolved from lysosomal ancestors to enzymes capable of hydrolyzing ingested food [169]. Cysteine cathepsins with digestive functions are found in insects from the Cucujiformia infraorder and are thought to be an evolutionary response to a seed diet rich in serine peptidase inhibitors [170,171] as well as more efficient hydrolysis of seed storage proteins [172]. A detailed study of cysteine cathepsins in the tenebrionid beetles *Tenebrio molitor* and *T. castaneum* from Cucujiformia revealed expansions of genes encoding cysteine digestive cathepsins [173,174]. Cysteine cathepsins in *T. castaneum* larvae are important components of adaptive responses in overcoming the effect of dietary protease inhibitors [175]. *R. dominica* does not belong to the Cucujiformia infraorder, and only serine peptidases, trypsin- and chymotrypsin-like, have been described so far in this species [134,135]. We annotated and evaluated cysteine peptidase genes in the *R. dominica* genome for functions using structural comparisons to model enzymes and gene expression analysis.

**Figure 11:**
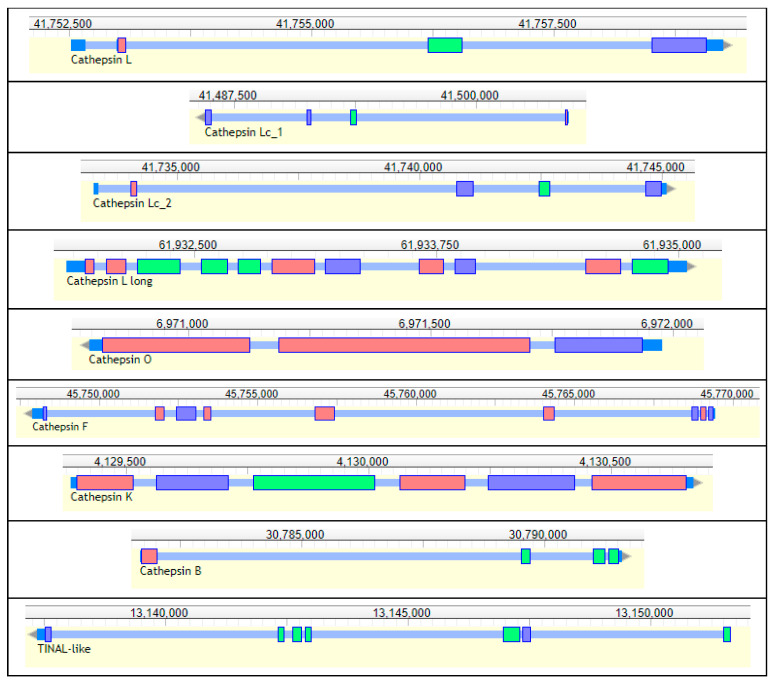
Structure of *R. dominica* cathepsins genes. Short, multicolored rectangles represent exons, tiny blue regions between them represent introns, and terminal dark blue sites represent UTRs. Upper numbers are sequence coordinates in scaffolds.

Nine genes encoding cysteine peptidases (cathepsins) from the C1 papain family were annotated in the genome of *R. dominica*. (Table 5, Figure 11). Seven genes belonged to the cathepsin L-like subfamily, and two were from the cathepsin B-like subfamily [176]. We found one typical animal cathepsin L (*RdL_97*), two related to insect cathepsin L (*RdLc1_97*, *RdLc2_97*), a cathepsin L with a typical long proregion (*RdLl_3*), as well as cathepsin F (*RdF_3*), cathepsin O (*RdO_135*), and cathepsin I (*RdI*, an insect variant of cathepsin K). Those from the cathepsin B subfamily included a typical cathepsin B (*RdB_100*) and a conserved inactive cathepsin—TINAL-like protein (*RdTINAL-like_3*), in which the active site Cys residue is replaced by a Ser [177]. Genes encoding shorter typical cathepsin L peptidases *RdL_97*, *RdLc1_97*, and *RdLc2_97* were found on the same scaffolds in close proximity, while the other cathepsins were scattered in other scaffolds. 

The annotation of *R. dominica* cathepsins was confirmed by a phylogenetic analysis with the cysteine cathepsins of *T. castaneum*, which have been functionally characterized in detail [110,173,174,175] (Figure S8). All *R. dominica* cathepsins clustered with the corresponding conserved cathepsins from *T. castaneum* that were expressed throughout the majority of life stages, including feeding and nonfeeding, and presumably belong to lysosomal or regulatory cathepsins [174]. Most *R. dominica* cathepsins were highly similar (bootstrap values > 70) to the orthologous *T. castaneum* cathepsins, with one exception with lower interspecies similarity, *RdL_97*. The major *T. castaneum* digestive cathepsin L genes, *TcL_NP_001164001* and *TcL_NP_001164314*, clustered with an inactive cathepsin L homolog, *TcLhom_XP_970773*, in a separate branch. The most highly expressed digestive *T. castaneum* cathepsin B, *TcB_XP_974298*, formed a separate branch with another digestive cathepsin *TcB_NP_001164205*. All the remaining *T. castaneum* cathepsin L and B genes were in separate clades with no orthologs among *R. dominica* cathepsins. 

Cysteine cathepsin genes from *R. dominica* demonstrated comparable levels of expression in whole adults and larval tissues, and none were expressed predominantly in the gut (Table 5). While sequences of *RdLc1_97* and *RdLc2_97* were highly similar, expression of their genes differed extremely, where *RdLc2_97* had relatively high levels of expression but expression of *RdLc1_97* was very low. From these observations we speculate that *RdLc1_97* is likely a non-functional paralog of *RdLc2_97* and their close localization in scaffold 97 support this hypothesis. 

##### Phosphine Resistance Genes

The strong resistance to phosphine phenotype in *R. dominica* is an inherited trait with incompletely recessive alleles at two autosomal loci [14]. The two resistance loci, *rph1* and *rph2* (resistance to phosphine 1 and 2), individually contribute to a weak resistance phenotype when homozygous but act synergistically to increase phosphine resistance when individuals are homozygous for both genes [15]. 

The *rph1* gene encodes a cytochrome-b5-related fatty acid desaturase (*Cyt-b5-r*, [178]) on Scaffold_2 that has two exons: a highly conserved cytochrome-b5 domain and a fatty acid desaturase domain. This gene contributes only low levels of resistance when rendered non-functional, often through deletions causing frameshifts or nonsense mutations. Larger deletion mutations can cause the region to be difficult to sequence in resistant insects, so the reference genome will be very helpful in identifying resistance alleles at the *rph1* locus in future projects.

The *rph2* locus is the major locus associated with high levels of resistance [16], and the gene encodes the dihydrolipoamide dehydrogenase (DLD) enzyme located on Scaffold_98. This enzyme participates in several key steps of core metabolism and is highly conserved amongst eukaryotes, where it is essential to life. This may explain the slower rates of evolution of resistance at *rph2* than *rph1*. Resistance from the *rph2* locus arises from specific point mutations in or around the active site of DLD that allow normal enzyme function but also reduce phosphine toxicity. 

### 3.3. Repeat Sequence/Structure

As mentioned in a previous section, the larger genome size of *R. dominica* relative to the model coleopteran *T. castaneum* is evidently due to an expansion of longer introns with repetitive sequences. In the final sections, we analyzed the repeat structures found in the *R. dominica* genome.

#### 3.3.1. Transposon Elements (TE)

The *R. dominica* genome contains more than 35.5% transposon-related sequences. However only about 9% of those sequences retain similarity to protein domains typical of transposons, and the remainder is composed of either simple repeats or remnants of ancient transposons (Table S5). The TE content is moderate considering the assembly quality measured by N_50_ or BUSCO. The size of the genome suggests genome expansion with mobile element proliferation. The genome has a relatively low GC content of 35%, similar to *T. castaneum*, which can result from high repeat content and lead to more efficient repeat proliferation, as repeats are usually AT rich and spread in AT rich regions.

**Figure 12:**
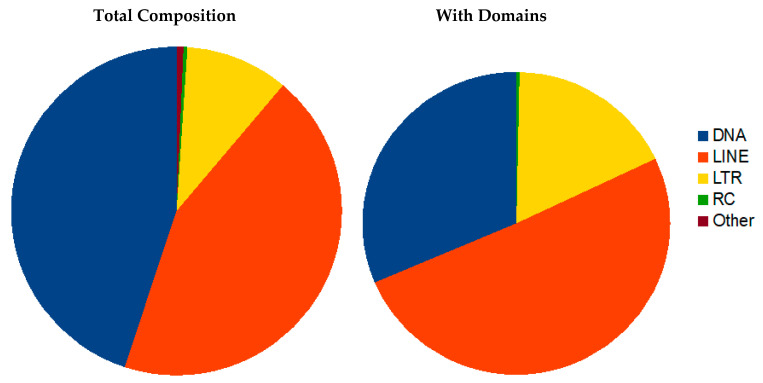
Composition of the transposable elements (TE) in the genome of *R. dominica*.

Most of the retrotransposons identified in *R. dominica* belong to LINE (Penelope, L2 and CR1) and LTR retrotransposon (Ty3/Gypsy) families, successful in many eukaryotic genomes (Figure 12). The genome has ten times more Ty3/Gypsy elements compared to Ty1/Copia. Members of the Tc1/Mariner superfamily are the most successful category of TE in *R. dominica*, with more than 119,000 remnant copies and 7000 copies within a transposase-conquered genome. Tc1/Mariner elements have been successful in colonizing genomes of all domains of life. DDE transposases from 15 super-families, are present, and representatives of 12 superfamilies retain coding regions that constitute a highly diverse TE landscape. Retroelements are more likely to retain protein coding regions than transposons with a “copy & paste” mode of mobilization. Additionally, the genome harbors a variety of less common TE, among them LTR DIRS elements, Cryptons and rolling circle Helitrons, and a wide variety of remnants of many DNA and LINE families. 

#### 3.3.2. Identification and Characterization of Satellite DNAs

The genome assembly was initially screened for satellite DNA by searching for sequence similarity with the Tandem Repeat Database (TRBD), which identified 25,810 satellite arrays. Delimiting monomer size to ≥ 100 bp and number of repeats to ≥ 2 reduced the number of arrays of interest to 7,347. Running of the Redundancy Tool, which removes the overlapping repeats, gave us a final number of 5,263 arrays which were analyzed in more detail.

We explored the distribution of monomer length within arrays and noticed that those with up to 400 bp-long monomers constituted 95% of arrays (Figure S9a). We also noticed that monomers with sizes between 120–130, 140–150 and 270–280 bp predominated (Figure S9b). The clustering tool integrated in TRDB resulted in the formation of 315 clusters, groups of arrays of tandem repeats that represent potential satDNA families. Ten clusters holding the largest number of arrays were chosen to be analyzed in more detail. The selected 10 most abundant clusters comprise 37% of all clustered arrays. Repetitive DNA families from 10 clusters have consensus monomer size between 123 and 294 bp, and their nucleotide sequences can be found in Table S6. 

The properties and abundance of the *R. dominica* DNA families are in Table 6. Tandem repeats belonging to these 10 families compose 0.5% of the *R. dominica* genome assembly. A NCBI blast search against nucleotide collection and expressed sequence tags databases showed multiple hits, with >93% of similarity in the genome of *Bombyx mori* when RD rep5 consensus sequence was used as a query. Additional RD rep5 monomer sequence blasts against *B. mori* Refseq project resulted in large number of hits with significant similarity (first 100 hits showing sequence similarity between 91 and 98%). Consensus sequences of other nine repeats showed no significant hits against aforementioned databases. These satellite DNA families show no similarities among themselves, except between RD rep6 and RD rep7 which exhibit 74.5% similarity to each other, with multiple short stretches of high sequence identity (Figure S9c). 

## 4. Discussion

The *R. dominica* genome sequence is significant from several aspects. This insect is a major stored product pest causing serious economic damage to grain worldwide. The insect is difficult to control because the immature stages feed within the grain kernel, and because it has become resistant to many contact insecticides and fumigants. Our assembly length is near the predicted genome size for *R. dominica* and contains presumably scaffolded chromosomes. The *R. dominica* genome assembly is the most contiguous beetle assembly published to date, and the first representative assembly from the Bostrichidae family. 

Our annotation study of insecticide detoxification genes suggested that the ecological niche of an insect that spends much of its development within a grain kernel may have protected this insect from selection pressure for some insecticide-related gene expansions that have been observed in other Coleoptera species. *R. dominica* has fewer ABCC genes (14) compared to other coleopterans (24–37) and lacks the typical CES gene expansions found in other Coleoptera species (14 in *R. dominica* vs. 30–70 or more copies in other coleopterans). ABCC genes are multidrug transporters that are usually conjugated to chemicals for transport and are more abundant in the insect Malpighian tubules (reviewed in [179]). Insect esterases hydrolyze ester bonds, such as those in organophosphates, to an acid and alcohol, and resistant insects have expanded gene copies and/or amplified esterase gene expression (reviewed in [180]). Lack of gene expansions in these groups indicate that insecticide-resistant *R. dominica* insects depend on other mechanisms, and there is evidence for both phosphotriesterases and acetylcholinesterase as detoxification enzymes [22,23].

Phase II detoxification enzymes that directly conjugate toxins or metabolites of toxins [181] also were observed in the *R. dominica* genome. Arrays containing species-specific amplifications may represent evolutionary hotspots in the genome that encode enzymes for adaptation to biotic and abiotic stresses. Such regions could contain arrays of genes for insecticide resistance or resistance to allelochemicals associated with feeding niches. 

Deltamethrin resistance pfam domains also were found in the *R. dominica* genome, similar to domains found in the *prag01* gene, The *R. dominica* colony that was inbred for sequencing was from a lab colony with no known exposure to insecticides and is thus presumed to be susceptible to most insecticides, including deltamethrin. Furthermore, putative orthologs of these proteins exist in the genomes of most other beetle species, lending additional support to the idea that the mere presence of this domain in a genome is not necessarily associated with resistance to deltamethrin. However, the gene models and predicted amino acids sequences in the *R. dominica* genome can serve as references for susceptible populations for comparisons to populations where resistance to deltamethrin has emerged. 

Our effort marks the first complete chemoreceptor suite annotated from *R. dominica* and the Bostrichidae as a whole. We found that the chemoreceptor families of *R*. *dominica* are expanded but not notably so, numbering slightly above those of species with limited host breadth, such as *A*. *planipennis* or *D*. *ponderosae*, but below those of highly polyphagous species, such as *A*. *glabripennis* [35]. Chemoreceptors have been annotated from one other beetle pest of grain, *T*. *castaneum*, which encodes a similar number of IRs but roughly twice as many GRs and ORs [182]. However, many of the chemoreceptors in *T*. *castaneum* are not expressed at detectable levels [103,183]. Expression in *R*. *dominica* may operate similarly, given the high number of chemoreceptor genes (311) compared to those even moderately expressed in antennal tissues (~10, [43]).

The *R*. *dominica* genome encodes a higher percentage of OR pseudogenes compared to other annotated beetle genomes [66]. These pseudogenes are unusually recent, with the majority exhibiting only a single nonsense or missense mutation, and they might easily exist as functional alleles in some populations. This abundance of recent pseudogenes supports the hypothesis of interbreeding populations of wild beetles and populations that have colonized granaries. The former must maintain a broad and sensitive chemosensory system to seek and identify host material, while the latter experience a ubiquitous food supply, which might relax selection on chemosensory genes and spawn novel pseudogenic alleles. Future chemosensory research could better resolve this issue with targeted resequencing of presumed PSE in natural and pest populations of *R*. *dominica*.

Annotation of potential digestive enzymes in the *R. dominica* genome indicate gene expansions of GHs including α-amylases, maltases, chitinases, α-glucosidases, and α-mannosidases to efficiently hydrolyze carbohydrates found in cereals. The lack of PCWDEs in the genome of *R. dominica* is further evidence that this insect does not utilize woody substrates as a food resource. There are several possible explanations for the wood-boring behavior that has been documented in the field previously. First, it is possible that the field insects were misidentified as *R. dominica*. Several powderpost beetles in the family Bostrichidae bear resemblance to *R. dominica* and feed on wood and wood products [184]. Second, facultative symbionts associated with *R. dominica* in the field may produce PCWDEs that enable them to digest a broader range of food resources. Symbiotic relationships between wood-feeding beetles and microbes are common in nature and can facilitate niche expansion [185]. Finally, it is possible that *R. dominica* does chew and tunnel into woody substrates in the field for refugia but does not actually digest lignocellulose or other cell wall polysaccharides as they pass through the gut. *R. dominica* can tunnel into twigs from maples, oaks, pines, and cedars collected from the field in Kansas, but survival is low, and no progeny are produced [8], suggesting that they cannot exclusively use woody plant materials as food resources.

*R. dominica* digestive SPs evidently serve as the major digestive enzymes for cereal proteins. There were 32 genes encoding single-domain SPs and two SPH genes (27% of total annotated SPs and related enzymes) that were highly expressed in larval gut tissue, with an average of more than 120-fold higher expression in the gut compared to that found in the head or carcass. Digestive SPs were found in clusters within scaffolds. One PSP serine peptidase, a DPP4 enzyme, was expressed mostly in larval gut tissue and is a candidate for hydrolyzing the large number of bonds formed by proline residues found in cereal proteins. Twenty genes encoding metalloexopopeptidases (carboxypeptidase and aminopeptidase) also had higher gut expression and likely further process cereal seed proteins. 

All predicted *R. dominica* cysteine cathepsins were functionally annotated as conserved lysosomal and/or regulatory genes. There were no digestive cathepsins in the *R. dominica* genome, in agreement with biochemical data [134,135]. This digestive system distinguishes *R. dominica* from the tenebrionids *T. molitor* and *T. castaneum* from the infraorder Cucujiformia, where the main digestive peptidases are cysteine cathepsins L and B with extremely high level of gene expression [125,173,174,175]. Digestive cysteine cathepsins are responsible for the hydrolysis of the main storage proteins of cereal seeds, which are the main dietary proteins of stored product pests [172]. In the absence of cysteine digestive peptidases, digestion of protein in *R. dominica* is predicted to be by serine peptidases adapted to successfully consume dry grains, as was demonstrated for the hemipteran cereal pest *Eurygaster integriceps* [186]. Bostrichoidea has been placed near or among early-divergent Polyphaga by molecular phylogenetic analyses [187]. Our data on the more ancient serine peptidase-based protein digestion found in *R. dominica* are consistent with the molecular phylogeny that distinguishes bostrichids from the evolved cysteine peptidase protein digestion described in Cucujiformia. 

Some *R. dominica* AQPs had only one or no conserved NPA sites, suggesting that solute transport activity for AQP12L and the Eglps from *R. dominica* may have expanded function beyond water and/or glycerol-specific channels. Although these proteins likely play critical functions in water homeostasis in *R. dominica*, additional functional studies are needed to establish precise roles they play in the unique biology of this stored-product insect.

We provided the first sequence of a potential Cry toxin binding cadherin-like protein from the Bostrichidae family, *RdCad1*, which formed a distinct clade among coleopteran cadherins. Comparing the sequence to other insect BtR1-like cadherins indicated that the lengths of individual exons varied significantly among the orders (e.g., lepidopteran vs. coleopteran cadherins), while lengths were more similar within orders. Predicted TBR regions had similarity to *BtR-1* from other Lepidoptera and Coleoptera insects. However, a region within TBR3 may explain the differential toxicity of Cry3Aa in stored product insects [167] although biochemical studies are needed to confirm the role of *RdCad1* in Bt toxicity. 

Delineation of phosphine resistance genes has been useful in monitoring the movement and development of strong phosphine resistance in populations with low frequencies. For example, a survey of *R. dominica* populations on organic farms with no prior history of phosphine use was conducted, first in 2006 and again in 2011 [188]. The study found that markers linked to *rph2* alleles significantly increased over the period, indicating movement of resistant individuals from nearby storage locations in the landscape. Further surveys using genotyping-by-sequencing methods have shown that phosphine resistance often arises independently and then spreads across large areas through movement of grain and natural dispersion [189,190]. Having a reference genome will assist in determining how often independent outbreaks occur and how they may be related as well as aid in characterizing the population genetics of resistance spread globally.

More than a third of the *R. dominica* genome sequences are related to transposons, but only 9% of the transposons have typical protein domains. Genome expansion in this insect has occurred via mobile element proliferation. Most retrotransposons were from the LINE and LTR families, mainly from the Tc1/Mariner superfamily. DDE transposases were from 15 superfamilies in a highly diverse TE landscape. These observations were reflected in several of the annotation datasets, in which longer genes compared to orthologs in other species were due to increased intron size due to TEs. 

The more frequent monomer lengths of satellites found in the assembled *R. dominica* genome were mostly consistent with the previously observed phenomena that certain monomer sizes in satDNA arrays are evolutionarily favored corresponding to the nucleosomal length [191,192]. We selected the 10 most prominent DNA families of tandem repeats that constituted 0.5% of the assembled genome. This analysis was on the assembled genome, and we recognize that limitations in sequencing and assembly of arrays composed of highly similar tandem repeats are known to cause satDNA sequences to be seriously underrepresented in genome sequencing outputs. This particularly applies for sequences located in heterochromatic genome compartment. For example, the major satDNA of the beetle *T. castaneum* was experimentally estimated to compose about 17% of the genome [193], while in the assembled genome it is represented only as 0.3% [194], with only approximately two-thirds of the estimated genome sequence assembled [110]. A more detailed and thorough satellitome profiling of unassembled reads and additional long read data are needed for a future more comprehensive study.

The presence of RD rep5 in both *R. dominica* (Coleoptera) and *B. mori* (Lepidoptera) is quite interesting. Taking into consideration that these species are very distantly related, we conclude that this satellite sequence may originate in the common ancestor of these two insect groups. If so, this satDNA sequence exhibits extraordinary long preservation throughout long evolutionary periods, with very high nucleotide sequence conservation. The similarity between RD rep6 and RD rep7 sequences indicates that they have originated from the same ancestral sequence, which subsequently diverged into two related families sharing sequence homology. Interestingly, despite the several indel events, accumulation of mutations throughout the monomer sequence is evidently non-random, resulting in the conserved blocks shared by the two families. Conserved segments are sometimes known to have some functional roles, for example, in DNA-protein interactions that may cause their persistence. Such an example is CENP-B protein, which plays a role in centromere formation, recognizing and binding the CENP-B box present within the higher primate alpha-satellite DNA [49]. 

## 5. Conclusions

The *R. dominica* genome sequence provides insights into an insect that has successfully adapted to survive on stored grain with immature development within the kernel. While over 300 chemoreceptor genes were identified in the genome, this beetle is unique among the studied Coleoptera with about 15% OR pseudogenes carrying a recently acquired single-point mutation, indicative of interbreeding in a closed system, such as granaries and relaxed selection on chemosensory genes. Annotation of peptidase and carbohydrase genes in *R. dominica* provided support for an expanded repertoire of enzymes that are necessary to reduce the impact of enzyme inhibitors in cereals, and enzymes adapted to efficiently hydrolyze cereal proteins and carbohydrates. The expansion of some insecticide-related genes found in other coleopteran genomes was not found in the *R. dominica* genome assembly and may be a result of development in the somewhat ecologically protected niche of the stored-grain environment. Presumably, *R. dominica* larvae developing within the kernel have reduced selection pressure from spray-insecticides, which may have contributed to the loss of expansion of ABCC family genes and some carboxylesterases compared to those in other coleopterans. Annotation of aquaporin genes in *R. dominica* suggest that larvae have adapted some genes to maintain water homeostasis in grain storage, which is an extremely arid environment. Analysis of the sequence of a potential Bt Cry toxin-binding protein, RdCad1, indicated that the first BtR1 sequence from a bostrichid may be unique from those previously described in cucujiformian coleopterans and may explain the differential toxicity of Cry toxins against this coleopteran storage pest. Signatures of repetitive sequences found in the genome may provide insights into the evolutionary relationship of bostrichid insects.

Populations of *R. dominica* resistant to various insect control products are economically costly and difficult to control. Sequencing the genome was a major impetus to replace or complement insect control with a more targeted approach based on genetic vulnerabilities that underly the biology of *R. dominica*. With such an approach, we move closer to products that will provide greater efficacy, lower non-target effects, and circumvent resistance. Data from this genome project provide the basis for such a targeted approach.

## Figures and Tables

**Table 1:** *Rhyzopertha dominica* genome assembly metrics.

Assembly	# Scaffolds	Total Mb	N_50_^1^ (Mb)	L_50_^1^	N_90_^1^ (Mb)	L_90_^1^	Longest Scaffold	BUSCO ^2^
CANU	1861 ^3^	493	0.87	158	0.15	627	5,205,710	99.3
Chicago/HiRise	948	493	7.32	20	1.11	84	27,933,969	99.4
Hybrid ^4^	336	479	7.44	19	1.48	74	27,934,817	97.6
Hi-C/HiRise	139	479	53.6	4	15.9	9	82,855,609	98.5

^1^ N_50_, the scaffold length such that the sum of the lengths of all scaffolds of this size or larger is equal to 50% of the total assembly length; N_90_, the scaffold length such that the sum of the lengths of all scaffolds of this size or larger is equal to 90% of the total assembly length; L_50_, the smallest number of scaffolds that make up 50% of the total assembly length; L_90_, the smallest number of scaffolds that make up 90% of the total assembly length. ^2^ [47], Insecta dataset. ^3^ Canu produces only contigs so the statistics for it reflect only contigs, not scaffolds. ^4^ Hybrid assembly with Dovetail Chicago scaffolds and MiSeq data in SeqManNGen (DNAStar Lasergene, Madison, WI, USA).

**Table 2:** Number of annotated genes in ABC transporter subfamilies in various coleopterans and *Drosophila melanogaster*.

Species	Subfamily								Total	Reference
	A	B	C	D	E	F	G	H		
*Rhyzopertha dominica*	3	6	14	2	1	3	13	3	45	This study
*Diabrotica v. virgifera*	4	7	32	2	1	3	12	4	65	[89] *
*Aethina tumida*	4	6	24	2	1	3	13	3	56	[90]
*Tribolium castaneum*	10	6	35	2	1	3	14	3	74	[61]
*Altica viridicyanea*	8	9	37	2	1	3	8	1	69	[91]
*Chrysomela populi*	5	8	29	2	1	3	14	3	65	[92] *
*Drosophila melanogaster*	10	8	14	2	1	3	15	3	56	[93]

* Estimated based on transcriptome data.

**Table 3:** General characteristics of *R. dominica* PSPs (active site residues, RNA expression and signal peptide prediction).

Type	MEROPS	Peptidase *	*R. dominica* Sequence ID	Active Site Residues	RNA Expression (RPKM)			Signal Peptide (Amino Acid)
					Gut	Carcass	Head	
Serine	S9	POP	*RDOM022815*	SDH	40.0	66.0	179	n/a
		DPP 4	*RDOM016604*	SDH	29.0	15.0	6.00	13
			*RDOM017825*	SDH	13.0	10.0	9.00	n/a
		DPP 9	*RDOM002099*	SDH	4.40	4.30	6.00	n/a
		DPP 10	*RDOM007500*	GDH	0.60	1.30	1.30	n/a
			*RDOM016697*	GDH	0.04	11.0	26.0	n/a
			*RDOM007053*	SDH	0.50	1.00	1.00	n/a
	S28	PRCP	*RDOM021888*	SDH	25.0	47.0	60.0	18
			*RDOM004644*	SDH	56.0	105	79.0	18
Metal-dependent	M24	APP1	*RDOM014136*	DDHEE	0.50	1.40	0.60	n/a
			*RDOM016283*	DDHEE	50.0	29.0	29.0	n/a
		APP3	*RDOM000819*	DDHEE	246	222	88.0	n/a
		Prolidase	*RDOM021565*	DDHEE	182	74.0	71.0	n/a

* POP—prolyloligopeptidase, DPP—dipeptidylpeptidase, PRCP—prolylcarboxypeptidase, APP—aminopeptidase P.

**Table 4:** *Rhyzopertha dominica* aquaporins (AQPs) predicted from manual genome annotation.

Name	Position	Intron #	CDS (bp)	Residues
*RDO_Drip*	Scaffold_97:54496632–54558762 − strand	4	681	227
*RDO_Prip*	Scaffold_97:54640277–54649322 + strand	3	819	272
*RDO_Bigbrain*	Scaffold_5:3510369–3521423 − strand	3	1161	386
*RDO_AQP12L*	Scaffold_2:13423224–13433006 − strand	4	924	307
*RDO_Eglp1*	Scaffold_1:3563523–3572656 + strand	5	942	313
*RDO_Eglp2*	Scaffold_1:34240108–34250443 + strand	4	660	219
*RDO_Eglp3*	Scaffold_1:3546737–3555431 + strand	6	888	295
*RDO_Eglp4*	Scaffold_1:26416818–26424599 − strand	4	831	276

**Table 5:** General characteristics of *R. dominica* cathepsins (gene coordinates, expression and enzyme active site residues).

Gene names	Annotation	Gene Coordinates	Expression (RPKM)				Active Site Residues
			Adult	Gut ^1^	Carcass ^1^	Head ^1^	
*RdL_97*	Cathepsin L	Scaffold_97:41752520–41759245 + strand	305	202	272	359	QCHN
*RdLc1_97*	Cathepsin Lc1	Scaffold_97:41485991–41504771 − strand	5.79	0	2.17	0.49	QCHN
*RdLc2_97*	Cathepsin Lc2	Scaffold_97:41733271–41745109 + strand	115	14.8	903	335	QCHN
*RdLl_3*	Cathepsin L1	Scaffold_3:61931842–61935045 + strand	114	160	145	108	QCHN
*RdO_135*	Cathepsin O	Scaffold_135:6970795–6971976 − strand	12.0	7.41	8.53	7.30	QCHN
*RdF_3*	Cathepsin F	Scaffold_3:45747905–45769454 − strand	94.8	57.2	176	213	QCHN
*RdI_5*	Cathepsin I	Scaffold_5:4129386–4130669 + strand	9.99	0.21	0.58	0.31	QCHN
*RdB_100*	Cathepsin B	Scaffold_100:30781674–30791599 + strand	272	729	571	164	QCHN HH
*RdTINAL-like_3*	Cathepsin B TINAL-like	Scaffold_3:13137364-13151656 − strand	32.3	27.4	35.3	43.9	QSHN

^1^ Tissues were from the same group of dissected larvae.

**Table 6:** Characteristics, organizational properties, and abundance of 10 major families of repeats found in the *R. dominica* genome.

Repeat Name	Consensus Monomer Length (bp)	Number of Arrays in a Cluster	Total Number of Monomers	Maximum Number of Monomers in an Array	Average Number of Monomers in an Array	AT Content of Consensus Sequence (%)	Average Monomer Similarity (%)	Genome Occupancy (bp)	% of the Assembled Genome
RD rep1	147	320	1836.8	37.1	5.7	52.4	93.6	270,614	0.0565
RD rep2	123	308	3765.4	230.0	12.2	61.0	78.4	462,627	0.0965
RD rep3	126	266	3389.4	190.7	12.7	63.5	85.2	418,239	0.0873
RD rep4	126	182	2390.3	173.5	13.1	65.1	91.1	298,197	0.0622
RD rep5	127	107	429.3	11.3	4.0	55.1	91.9	54,080	0.0113
RD rep6	272	98	751.1	58.3	7.6	61.4	89.0	203,159	0.0424
RD rep7	294	97	1350.2	59.7	13.9	59.5	86.6	397,984	0.0831
RD rep8	110	97	260.2	7.0	2.7	63.6	96.4	28,933	0.0060
RD rep9	135	95	734.4	23.7	7.7	65.2	89.4	95,988	0.0200
RD rep10	153	89	898.4	31.7	10.1	54.9	93.6	137,075	0.0286

## Data Availability

The datasets supporting the results presented in this article are available in the NCBI repository: Dataset Brenda Oppert; 2017, Lesser Grain Borer Genome Assembly December 2017, NCBI; SUB2507831; Dataset Brenda Oppert, 2017, *Rhyzopertha dominica* isolate: LGB_DtHiC_Dec2017 Genome sequencing, PRJNA449115; Dataset Brenda Oppert and Lindsey Perkin, 2019, Rdo Transcriptome tissue, PRJNA598370. Gene predictions are deposited at Ag Data Commons (doi.org/10.15482/USDA.ADC/1524749). The datasets supporting the conclusions of this article are included within the article and its additional files.

## Supplementary Materials

The following supporting information can be downloaded at: https://www.mdpi.com/article/10.3390/genes13030446/s1, Figure S1: Link density histogram of Hi-C scaffolded *R. dominica* genome assembly. Figure S2: Conserved domain analysis of the ABC transporters of *R. dominica*. The numbers in parantheses indicate the number of ABC transporters in each subfamily. TMD: transmembrane domain; NBD: nucleotide binding domain. Figure S3: Phylogenetic comparison of UGT sequences found on scaffold 135 of the *R. dominica* genome and grouping within a single clade and those found in other clades (*Nicrophorus vespilloides*, *Callosobruchus maculatus*, *Ignelater luminosus*, *Photinus pyralis*, *L. decemlineata,* and *D. virgifera virgifera*). Figure S4: Phylogenetic analysis of *R. dominica* aquaporins. The evolutionary history of representative insect aquaporins was inferred using the Neighbor-Joining method [195]. The bootstrap consensus tree inferred from 500 replicates is taken to represent the evolutionary history of the taxa analyzed [196]. Branches corresponding to partitions reproduced in less than 50% bootstrap replicates are collapsed. The percentage of replicate trees in which the associated taxa clustered together in the bootstrap test (500 replicates) are shown next to the branches. The evolutionary distances were computed using the Dayhoff matrix-based method [197] and are in the units of the number of amino acid substitutions per site. The analysis involved 101 amino acid sequences. All positions containing gaps and missing data were eliminated. There were a total of 88 positions in the final dataset. Evolutionary analyses were conducted in MEGA7 [67]. Figure S5: cDNA sequence and deduced open reading frame of the *R. dominica* BtR1-like cadherin gene (*RdCad1*). The predicted domains are underlined: the membrane signal peptide with a dashed line, the MPED domain with a solid line, and CYT domain with a dotted line. The start of each of the twelve cadherin repeat domains is marked with a solid arrow. The TM domain region is shaded gray. Predicted *N*-and *O*-glycosylation sites are marked by asterisks. The putative toxin binding regions (TBR1-3) are designated by the rounded rectangle below the sequence. Figure S6: Alignment of the putative toxin binding regions (TBR1; 2 and 3) of insect midgut cadherins—the Cry toxin receptors. Cadherins include those from *M. sexta* (MsBtR1, AAM21151), *Lymantria dispar* (LdCad, AF317621_1), *Helicoverpa armigera* (HaBtR, ACF94775), *Heliothis virescens* (HvCad, AAV80768), *Tribolium castaneum* (TcCad1, XP_971388), *Tenebrio molitor* (TmCad1, DQ988044), *Alphitobius diaperinus* (AdCad1, AHJ10508), and *R. dominica* (RdCad1). Critical toxin-binding region of EC1-10 cadherin of chrysomelid beetles *Diabrotica virgifera* (DvCad, ABU50692) and the putative Colorado potato beetle (*Leptinotarsa decemlineata*) ortholog (CPBCad, XP_023015891) demonstrate similarity with an epitope of TBR2, and there was no obvious match to TBR3. The TBRs residues that may potentially affect the Cry toxin interaction are outlined in red boxes (refer to the text for more details). Figure S7: Phylogenetic analysis of insect cadherin protein sequences. Phylogenetic data obtained from ClustalW-aligned sequences were processed using Maximum Likelihood tree builder algorithm (MEGA X) with 500 bootstrap iterations. Protein sequences used in the analysis were from *M. sexta* (MsBtR1), *Bombyx mori* (BmCad, BAA99404), *H. virescens* (HvCad), *H. armigera* (HaBtR), *L. dispar* (LdCad), *Ostrinia furnacalis* (OfCad), *O. nubilalis* (OnCadA1, AAT37678), *Plutella xylostella* (PxCad, ABU41413), *Chilo suppressalis* (CsCad, AAM78590), *R. dominica* (RdCad1), *A. diaperinus* (AdCad1); *T. molitor* (TmCad1), *T. castaneum* (TcCad1), *Leptinotarsa decemlineata* (CPBCad), and *D. virgifera* (DvCad). The *T. castaneum* cadherin (TcCad88C, XP_971786) was used as an outgroup. The tree was drawn to scale, with branch lengths showing the number of substitutions per site. Figure S8: Phylogenetic analysis of *R. dominica* and *T. castaneum* cathepsins. Maximum likelihood tree with 500 iterations of bootstrapping were used; bootstrapping values are represented as a percentage (0–100) by each branch. Evolutionary distances are represented by branch length. Figure S9: Evaluation of monomers in the *R. dominica* genome. (a) Distribution of number of arrays according to the monomer length. Monomer size of up to 2000 bp was examined (shown only up to 1100 bp due to the decreasing abundance). (b) Number of monomers in relation to the monomer size. (c) Alignment of consensus sequences of two related families, RD rep6 and rep7. Table S1: Annotation notes, expression data, and nucleotide and protein sequences for the chemosensory gene families of OR, GR, and IR in *R. dominica*. Table S2: Comparison of chemosensory genes in the *R. dominica* genome from the present study to the results of [43]. Table S3: Peptidase genes annotated in the genome of *R. dominica.* Table S4: Genomic organization of *R. dominica* Bt-R1-like cadherin gene. The number and length of the deduced exons and their encoding cadherin domains are shown. All the intron-exon borders provided were followed the GT-AG rule for RNA 5’ acceptor and 3’ donor splice sites. The exon sequences are shown in upper case, coding regions for the first and last exons are shown in parentheses, asterisk indicates that the lengths provided for 5’- and 3’-UTRs were only approximate. The start and stop codon (TGA) were underlined and shaded grey, respectively. The transposable elements (TE) predicted by Censor (https://www.girinst.org/censor/, accessed on 25 December 2020) inside the introns of cadherin gene are provided. Table S5: Composition of repetitive elements in the *R. dominica* genome. Table S6: Consensus monomer sequences of the 10 most prominent repeat families in the *R. dominica* genome. File S1: Extraction and dissection of *R. dominica* larvae from wheat kernels.

## Abbreviations

ABC	ATP binding cassette
APP	aminopepdidase P
AQP	Aquaporin
Bib	*Drosophila* big-brain
*Bt*	*Bacillus thuringiensis*
BUSCO	Benchmarking Universal Single-Copy Orthologs
CGAHR	Center for Grain and Animal Health Research
COesterases	Carboxylesterases
CYT	Cytoplasmic
DLD	dihydrolipoamide dehydrogenase
DPP	dipeptdidylpeptidases
Drip	*Drosophila* integral protein
EC	Ectodomain
Eglps	entomoglyveroporins
FAP	fibroblast activation protein
GH	glucosyl hydrolase
GLPs	aquaglyceroporins
GR	gustatory receptor
INT	internal exons
IR	ionotropic receptor
LINE	long interspersed nuclear element
LTR	long terminal repeat;
MPED	membrane proximal extracellular domain
MRPs	multidrug resistance proteins
NBD	nucleotide binding domain
NPA motif	asparagine-proline-alanine motif
NTE	N-terminal
OR	odorant receptor
PCWDE	plant cell wall degrading enzymes
PSPs	proline-specific peptidases
PER	period to eliminate multiple reporting of repeats
POP	prolyloligopeptidase
PRCP	prolylcarboxypeptidase
Prip	*Pyrocoelia rufa* integral protein
RdCad1	BtR1-like cadherin gene
RNAi	RNA interference
RPKM	reads per kilobase per million mapped reads
satDNA	satellite DNA
SP	single-domain serine peptidases
SPHs	non-catalytic SP homologs
TBR	toxin-binding regions
TE	transposable elements
TIR	terminal inverted repeats
TM/TMD	transmembrane/transmembrane domain
TRBD	tandem repeat database
UGTs	Uridine 5′-diphospho-glucuronosyltransferase

## References

[1] Lucas H. (1849). Crustacés, arachnides, myriopodes et hexapodes: Exploration scientifique de l’Algérie pendant les années 1840, 1841, 1842. Sci. Phys. Zool. Hist. Nat. Animaux Articul..

[2] Fields P., Van Loon J., Dolinski M., Harris J., Burkholder W. (1993). The distribution of *Rhyzopertha dominica* (F.) in Western Canada. Can. Entomol..

[3] Edde P. (2012). A review of the biology and control of *Rhyzopertha dominica* (F.) the lesser grain borer. J. Stored Prod. Res..

[4] Toews M.D., Campbell J.F., Arthur F.H., Ramaswamy S.B. (2006). Outdoor flight activity and immigration of *Rhyzopertha dominica* into seed wheat warehouses. Entomol. Exp. Appl..

[5] Schwardt H.H. (1993). Life history of the lesser grain borer. J. Kans. Entomol. Soc..

[6] Edde P.A., Phillips T.W. (2006). Potential host affinities for the lesser grain borer, Rhyzopertha dominica: Behavioral responses to host odors and pheromones and reproductive ability on non-grain hosts. Entomol. Exp. Appl..

[7] Wright V.F., Fleming E.E., Post D., Wright F. (1990). Survival of *Rhyzopertha dominica* (Coleoptera, Bostrichidae ) on fruits and seeds collected from woodrat nests in Kansas. J. Kans. Entomol. Soc..

[8] Jia F., Toews M.D., Campbell J.F., Ramaswamy S.B. (2008). Survival and reproduction of lesser grain borer, *Rhyzopertha dominica* (F.) (Coleoptera: Bostrichidae) on flora associated with native habitats in Kansas. J. Stored Prod. Res..

[9] Mahroof R.M., Edde P., Robertson B., Puckette J.A., Phillips T.W. (2010). Dispersal of *Rhyzopertha dominica* (Coleoptera: Bostrichidae) in different habitats. Environ. Entomol..

[10] Quellhorst H., Athanassiou C.G., Zhu K.Y., Morrison W.R. (2021). The biology, ecology and management of the larger grain borer, *Prostephanus truncatus* (Horn) (Coleoptera: Bostrichidae). J. Stored Prod. Res..

[11] Opit G.P., Phillips T.W., Aikins M.J., Hasan M.M. (2012). Phosphine resistance in *Tribolium castaneum* and *Rhyzopertha dominica* from stored wheat in Oklahoma. J. Econ. Entomol..

[12] Lorini I., Collins P.J., Daglish G.J., Nayak M.K., Pavic H. (2007). Detection and characterisation of strong resistance to phosphine in Brazilian *Rhyzopertha dominica* (F.) (Coleoptera: Bostrychidae). Pest Manag. Sci..

[13] Benhalima H., Chaudhry M., Mills K., Price N. (2004). Phosphine resistance in stored-product insects collected from various grain storage facilities in Morocco. J. Stored Prod. Res..

[14] Collins P.J., Daglish G., Bengston M., Lambkin T.M., Pavic H. (2002). Genetics of resistance to phosphine in *Rhyzopertha dominica* (Coleoptera: Bostrichidae). J. Econ. Entomol..

[15] Schlipalius I.D., Chen W., Collins P.J., Nguyen T., Reilly P.E.B., Ebert P.R. (2008). Gene interactions constrain the course of evolution of phosphine resistance in the lesser grain borer, *Rhyzopertha dominica*. Heredity.

[16] Schlipalius D.I., Valmas N., Tuck A.G., Jagadeesan R., Ma L., Kaur R., Goldinger A., Anderson C., Kuang J., Zuryn S. (2012). A core metabolic enzyme mediates resistance to phosphine gas. Science.

[17] Arthur F.H. (1996). Grain protectants: Current status and prospects for the future. J. Stored Prod. Res..

[18] Kavallieratos N.G., Athanassiou C.G., Arthur F.H. (2015). Efficacy of deltamethrin against stored-product beetles at short exposure intervals or on a partially treated rice mass. J. Econ. Entomol..

[19] Sehgal B., Subramanyam B., Arthur F.H., Gill B.S. (2014). Variation in susceptibility of laboratory and field strains of three stored-grain insect species to β -cyfluthrin and chlorpyrifos-methyl plus deltamethrin applied to concrete surfaces. Pest Manag. Sci..

[20] Arthur F. (2016). Efficacy of methoprene for multi-year protection of stored wheat, brown rice, rough rice and corn. J. Stored Prod. Res..

[21] Haliscak J.P., Beeman R.W. (1983). Status of malathion resistance in five genera of beetles infesting farm-stored corn, wheat, and oats in the United States. J. Econ. Entomol..

[22] Guedes R.N.C., Kambhampati S., Dover B.A., Zhu K.Y. (1997). Biochemical mechanism of organophosphate resistance in Brazilian and U. S. populations of *Rhyzopertha dominica* (F.) (Coleoptera: Bostrichidae). Bull. Entomol. Res..

[23] Guedes R.N., Zhu K., Kambhampati S., Dover B. (1998). Characterization of acetylcholinesterase purified from the lesser grain borer, *Rhyzopertha dominica* (Coleoptera: Bostrichidae). Comp. Biochem. Physiol. Part C Pharmacol. Toxicol. Endocrinol..

[24] Wang H.-T., Tsai C.-L., Chen M.-E. (2018). Nicotinic acetylcholine receptor subunit α6 associated with spinosad resistance in *Rhyzopertha dominica* (Coleoptera: Bostrichidae). Pestic. Biochem. Physiol..

[25] Sakka M.K., Riga M., Ioannidis P., Baliota G.V., Tselika M., Jagadeesan R., Nayak M.K., Vontas J., Athanassiou C.G. (2021). Transcriptomic analysis of s-methoprene resistance in the lesser grain borer, *Rhyzopertha dominica*, and evaluation of piperonyl butoxide as a resistance breaker. BMC Genom..

[26] Khorramshahi A., Burkholder W.E. (1981). Behavior of the lesser grain borer *Rhyzopertha dominica* (Coleoptera: Bostrichidae). J. Chem. Ecol..

[27] Williams H.J., Silverstein R.M., Burkholder W.E., Khorramshahi A. (1981). Dominicalure 1 and 2: Components of aggregation pheromone from male lesser grain borer *Rhyzopertha dominica* (F.) (Coleoptera: Bostrichidae). J. Chem. Ecol..

[28] Bashir T., Birkinshaw L., Hall D., Hodges R. (2001). Host odours enhance the responses of adult Rhyzopertha dominica to male-produced aggregation pheromone. Entomol. Exp. Appl..

[29] Nguyen D.T., Hodges R.J., Belmain S.R. (2008). Do walking *Rhyzopertha dominica* (F.) locate cereal hosts by chance?. J. Stored Prod. Res..

[30] Edde P., Phillips T.W., Robertson J.B., Dillwith J.W. (2007). Pheromone output by *Rhyzopertha dominica* (Coleoptera: Bostrichidae), as affected by host plant and beetle size. Ann. Entomol. Soc. Am..

[31] Dowdy A.K., Howard R.W., Seitz L.M., McGaughey W.H. (1993). Response of *Rhyzopertha dominica* (Coleoptera: Bostrichidae) to its aggregation pheromone and wheat volatiles. Environ. Entomol..

[32] Cordeiro E.M.G., Campbell J.F., Phillips T.W. (2016). Movement and orientation decision modeling of *Rhyzopertha dominica* (Coleoptera: Bostrichidae) in the grain mass. Environ. Entomol..

[33] Robertson H.M. (2019). Molecular evolution of the major arthropod chemoreceptor gene families. Annu. Rev. Entomol..

[34] Mitchell R.F., Andersson M.N. (2020). Olfactory genomics of the Coleoptera. Insect Pheromone Biochemistry and Molecular Biology.

[35] Andersson M.N., Keeling C.I., Mitchell R.F. (2019). Genomic content of chemosensory genes correlates with host range in wood-boring beetles (*Dendroctonus ponderosae*, *Agrilus planipennis*, and *Anoplophora glabripennis*). BMC Genom..

[36] Mitchell R.F., Hughes D.T., Luetje C.W., Millar J.G., Soriano-Agatón F., Hanks L.M., Robertson H.M. (2012). Sequencing and characterizing odorant receptors of the cerambycid beetle *Megacyllene caryae*. Insect Biochem. Mol. Biol..

[37] Antony B., Johny J., Montagné N., Jacquin-Joly E., Capoduro R., Cali K., Persaud K., Al-Saleh M.A., Pain A. (2021). Pheromone receptor of the globally invasive quarantine pest of the palm tree, the red palm weevil (*Rhynchophorus ferrugineus*). Mol. Ecol..

[38] Yuvaraj J.K., Roberts R.E., Sonntag Y., Hou X.-Q., Grosse-Wilde E., Machara A., Zhang D.-D., Hansson B.S., Johanson U., Löfstedt C. (2021). Putative ligand binding sites of two functionally characterized bark beetle odorant receptors. BMC Biol..

[39] Wang X., Wang S., Yi J., Li Y., Liu J., Wang J., Xi J. (2020). Three host plant volatiles, hexanal, lauric acid, and tetradecane, are detected by an antenna-biased expressed odorant receptor 27 in the dark black chafer *Holotrichia parallela*. J. Agric. Food Chem..

[40] Hou X.Q., Yuvaraj J.K., Roberts R.E., Zhang D.D., Unelius C.R., Löfstedt C., Andersson M.N. (2021). Functional evolution of a bark beetle odorant receptor clade detecting monoterpenoids of different ecological origins. Mol. Biol. Evol..

[41] Ji T., Xu Z., Jia Q., Wang G., Hou Y. (2021). Non-palm plant volatile α-pinene is detected by antenna-biased expressed odorant receptor 6 in the *Rhynchophorus ferrugineus* (Olivier) (Coleoptera: Curculionidae). Front. Physiol..

[42] Takada T., Sato R., Kikuta S. (2017). A mannitol/sorbitol receptor stimulates dietary intake in *Tribolium castaneum*. PLoS ONE.

[43] Mandiana Diakite M., Wang J., Ali S., Wang M. (2016). Identification of chemosensory gene families in *Rhyzopertha dominica* (Coleoptera: Bostrichidae). Can. Entomol..

[44] Schlipalius I.D., Cheng Q., Reilly P.E.B., Collins P.J., Ebert P.R. (2002). Genetic linkage analysis of the lesser grain borer *Rhyzopertha dominica* identifies two loci that confer high-level resistance to the fumigant phosphine. Genetics.

[45] Johnston J.S., Bernardini A., Hjelmen C.E. (2019). Genome size estimation and quantitative cytogenetics in insects. Program. Necrosis.

[46] Putnam N.H., O’Connell B.L., Stites J.C., Rice B.J., Blanchette M., Calef R., Troll C.J., Fields A., Hartley P.D., Sugnet C.W. (2016). Chromosome-scale shotgun assembly using an in vitro method for long-range linkage. Genome Res..

[47] Simão F.A., Waterhouse R.M., Ioannidis P., Kriventseva E.V., Zdobnov E.M. (2015). BUSCO: Assessing genome assembly and annotation completeness with single-copy orthologs. Bioinformatics.

[48] Lieberman-Aiden E., Van Berkum N.L., Williams L., Imakaev M., Ragoczy T., Telling A., Amit I., Lajoie B.R., Sabo P.J., Dorschner M.O. (2009). Comprehensive mapping of long-range interactions reveals folding principles of the human genome. Science.

[49] Masumoto H., Masukata H., Muro Y., Nozaki N., Okazaki T. (1989). A human centromere antigen (CENP-B) interacts with a short specific sequence in alphoid DNA, a human centromeric satellite. J. Cell Biol..

[50] Johnson A.D., Handsaker R.E., Pulit S.L., Nizzari M.M., O’Donnell C.J., de Bakker P.I.W. (2008). SNAP: A web-based tool for identification and annotation of proxy SNPs using HapMap. Bioinformatics.

[51] Oppert B., Morgan T. (2013). Improved high-throughput bioassay for *Rhyzopertha dominica* (F.) (Coleoptera: Bostrichidae). J. Stored Prod. Res..

[52] Perkin L.C., Oppert B. (2019). Gene expression in *Tribolium castaneum* life stages: Identifying a species-specific target for pest control applications. PeerJ.

[53] Mortazavi A., Williams B.A., McCue K., Schaeffer L., Wold B. (2008). Mapping and quantifying mammalian transcriptomes by RNA-Seq. Nat. Methods.

[54] Flynn J.M., Hubley R., Goubert C., Rosen J., Clark A.G., Feschotte C., Smit A.F. (2020). RepeatModeler2 for automated genomic discovery of transposable element families. Proc. Natl. Acad. Sci. USA.

[55] Smit A.F.A., Hubley R., Green P. RepeatMasker Open-4.0. http://www.repeatmasker.org.

[56] Stanke M., Steinkamp R., Waack S., Morgenstern B. (2004). AUGUSTUS: A web server for gene finding in eukaryotes. Nucleic Acids Res..

[57] Dobin A., Davis C.A., Schlesinger F., Drenkow J., Zaleski C., Jha S., Batut P., Chaisson M., Gingeras T.R. (2013). STAR: Ultrafast universal RNA-seq aligner. Bioinformatics.

[58] Cantarel B.L., Korf I., Robb S.M., Parra G., Ross E., Moore B., Holt C., Alvarado A.S., Yandell M. (2007). MAKER: An easy-to-use annotation pipeline designed for emerging model organism genomes. Genome Res..

[59] Altschul S.F., Gish W., Miller W., Myers E.W., Lipman D.J. (1990). Basic local alignment search tool. J. Mol. Biol..

[60] Chan P.P., Lowe T.M. (2019). tRNAscan-SE: Searching for tRNA genes in genomic sequences. Springer Protocols Handbooks.

[61] Broehan G., Kroeger T., Lorenzen M., Merzendorfer H. (2013). Functional analysis of the ATP-binding cassette (ABC) transporter gene family of Tribolium castaneum. BMC Genom..

[62] Grubbs N., Haas S., Beeman R.W., Lorenzen M.D. (2015). The ABCs of eye color in *Tribolium castaneum*: Orthologs of the *Drosophila* white, scarlet, and brown genes. Genetics.

[63] Lee E., Helt A.G., Reese J.T., Munoz-Torres M.C., Childers C.P., Buels R.M., Stein L., Holmes I.H., Elsik C.G., Lewis E.S. (2013). Web Apollo: A web-based genomic annotation editing platform. Genome Biol..

[64] Skinner M.E., Uzilov A.V., Stein L.D., Mungall C.J., Holmes I.H. (2009). JBrowse: A next-generation genome browser. Genome Res..

[65] Finn R.D., Bateman A., Clements J., Coggill P., Eberhardt R.Y., Eddy S.R., Heger A., Hetherington K., Holm L., Mistry J. (2014). Pfam: The protein families database. Nucleic Acids Res..

[66] Mitchell R.F., Schneider T.M., Schwartz A.M., Andersson M.N., McKenna D.D. (2020). The diversity and evolution of odorant receptors in beetles (Coleoptera). Insect Mol. Biol..

[67] Sievers F., Wilm A., Dineen D., Gibson T.J., Karplus K., Li W., Lopez R., McWilliam H., Remmert M., Söding J. (2011). Fast, scalable generation of high-quality protein multiple sequence alignments using Clustal Omega. Mol. Syst. Biol..

[68] Edgar R.C. (2004). MUSCLE: Multiple sequence alignment with high accuracy and high throughput. Nucleic Acids Res..

[69] Kumar S., Stecher G., Tamura K. (2016). MEGA7: Molecular evolutionary genetics analysis version 7.0 for bigger datasets. Mol. Biol. Evol..

[70] Kumar S., Stecher G., Li M., Knyaz C., Tamura K. (2018). MEGA X: Molecular evolutionary genetics analysis across computing platforms. Mol. Biol. Evol..

[71] Capella-Gutiérrez S., Silla-Martínez J.M., Gabaldón T. (2009). trimAl: A tool for automated alignment trimming in large-scale phylogenetic analyses. Bioinformatics.

[72] Price M.N., Dehal P.S., Arkin A.P. (2010). FastTree 2—Approximately maximum-likelihood trees for large alignments. PLoS ONE.

[73] Rambaut A., Drummond A.J. Molecular Evolution, Phylogenetics and Epidemiology. http://tree.bio.ed.ac.uk/software/figtree/.

[74] Li H., Durbin R. (2009). Fast and accurate short read alignment with Burrows–Wheeler transform. Bioinformatics.

[75] Li H., Handsaker B., Wysoker A., Fennell T., Ruan J., Homer N., Marth G., Abecasis G., Durbin R. (2009). The sequence alignment/map format and SAMtools. Bioinformatics.

[76] Haas B.J., Papanicolaou A., Yassour M., Grabherr M., Blood P.D., Bowden J., Couger M.B., Eccles D., Li B., Lieber M. (2013). De novo transcript sequence reconstruction from RNA-seq using the Trinity platform for reference generation and analysis. Nat. Protoc..

[77] Zhang Y., Park C., Bennett C., Thornton M., Kim D. (2021). Rapid and accurate alignment of nucleotide conversion sequencing reads with HISAT-3N. Genome Res..

[78] Kovaka S., Zimin A.V., Pertea G.M., Razaghi R., Salzberg S.L., Pertea M. (2019). Transcriptome assembly from long-read RNA-seq alignments with StringTie2. Genome Biol..

[79] Cao X., Jiang H., Brown S., Pfrender M. (2019). Integrated modeling of structural genes using MCuNovo. Insect Genomics. Methods in Molecular Biology.

[80] Warburton P.E., Giordano J., Cheung F., Gelfand Y., Benson G. (2004). Inverted repeat structure of the human genome: The X-chromosome contains a preponderance of large, highly homologous inverted repeats that contain testes genes. Genome Res..

[81] Fu L., Niu B., Zhu Z., Wu S., Li W. (2012). CD-HIT: Accelerated for clustering the next-generation sequencing data. Bioinformatics.

[82] Marchler-Bauer A., Derbyshire M.K., Gonzales N.R., Lu S., Chitsaz F., Geer L.Y., Geer R.C., He J., Gwadz M., Hurwitz D.I. (2015). CDD: NCBI’s conserved domain database. Nucleic Acids Res..

[83] Jurka J., Kapitonov V.V., Pavlicek A., Klonowski P., Kohany O., Walichiewicz J. (2005). Repbase update, a database of eukaryotic repetitive elements. Cytogenet. Genome Res..

[84] Gelfand Y., Rodriguez A., Benson G. (2007). TRDB—The Tandem Repeats Database. Nucleic Acids Res..

[85] Smith S.G., Brower J.H. (1974). Chromosome numbers of stored-product Coleoptera. J. Kans. Entomol. Soc..

[86] Guedes R.N.C., Dover B.A., Kambhampati S. (1996). Resistance to chlorpyrifos-methyl, pirimiphos-methyl, and malathion in Brazilian and U.S. populations of *Rhyzopertha dominica* (Coleopera: Bostrichidae). J. Econ. Entomol..

[87] Daglish G.J., Nayak M.K. (2018). Prevalence of resistance to deltamethrin in *Rhyzopertha dominica* (F.) in eastern Australia. J. Stored Prod. Res..

[88] Daglish G., Holloway J.C., Nayak M.K. (2013). Implications of methoprene resistance for managing *Rhyzopertha dominica* (F.) in stored grain. J. Stored Prod. Res..

[89] Adedipe F., Grubbs N., Coates B., Wiegmman B., Lorenzen M. (2019). Structural and functional insights into the Diabrotica virgifera virgifera ATP-binding cassette transporter gene family. BMC Genom..

[90] Evans J.D., McKenna D., Scully E., Cook S.C., Dainat B., Egekwu N., Grubbs N., Lopez D., Lorenzen M., Reyna S.M. (2018). Genome of the small hive beetle (*Aethina tumida*, Coleoptera: Nitidulidae), a worldwide parasite of social bee colonies, provides insights into detoxification and herbivory. GigaScience.

[91] Xue H.-J., Niu Y.-W., Segraves K.A., Nie R.-E., Hao Y.-J., Zhang L.-L., Cheng X.-C., Zhang X.-W., Li W.-Z., Chen R.-S. (2021). The draft genome of the specialist flea beetle *Altica viridicyanea* (Coleoptera: Chrysomelidae). BMC Genom..

[92] Strauss A.S., Wang D., Stock M., Gretscher R., Groth M., Boland W., Burse A. (2014). Tissue-specific transcript profiling for ABC transporters in the sequestering larvae of the phytophagous leaf beetle *Chrysomela populi*. PLoS ONE.

[93] Dean M., Rzhetsky A., Allikmets R. (2001). The human ATP-Binding Cassette (ABC) transporter superfamily. Genome Res..

[94] David J.-P., Ismail H.M., Chandor-Proust A., Paine M.J.I. (2013). Role of cytochrome P450s in insecticide resistance: Impact on the control of mosquito-borne diseases and use of insecticides on Earth. Philos. Trans. R. Soc. B Biol. Sci..

[95] Zhu F., Moural T.W., Shah K., Palli S.R. (2013). Integrated analysis of cytochrome P450 gene superfamily in the red flour beetle, *Tribolium castaneum*. BMC Genom..

[96] Jackson C.J., Liu J.-W., Carr P.D., Younus F., Coppin C., Meirelles T., Lethier M., Pandey G., Ollis D.L., Russell R.J. (2013). Structure and function of an insect -carboxylesterase (Esterase7) associated with insecticide resistance. Proc. Natl. Acad. Sci. USA.

[97] Rane R., Walsh T., Pearce S.L., Jermiin L., Gordon K., Richards S., Oakeshott J.G. (2016). Are feeding preferences and insecticide resistance associated with the size of detoxifying enzyme families in insect herbivores?. Curr. Opin. Insect Sci..

[98] McKenna D.D., Scully E.D., Pauchet Y., Hoover K., Kirsch R., Geib S.M., Mitchell R.F., Waterhouse R.M., Ahn S.-J., Arsala D. (2016). Genome of the Asian longhorned beetle (*Anoplophora glabripennis*), a globally significant invasive species, reveals key functional and evolutionary innovations at the beetle–plant interface. Genome Biol..

[99] Kaplanoglu E., Chapman P., Scott I., Donly C. (2017). Overexpression of a cytochrome P450 and a UDP-glycosyltransferase is associated with imidacloprid resistance in the Colorado potato beetle, *Leptinotarsa decemlineata*. Sci. Rep..

[100] Scully E.D., Geib S.M., Carlson E.J., Tien M., McKenna D., Hoover K. (2014). Functional genomics and microbiome profiling of the Asian longhorned beetle (*Anoplophora glabripennis*) reveal insights into the digestive physiology and nutritional ecology of wood feeding beetles. BMC Genom..

[101] Zhang J., Yang M., Wang W., Sun H., Xu Y., Ma L., Sun Y., Zhu C. (2011). *prag01*, a novel deltamethrin-resistance-associated gene from *Culex pipiens pallens*. Parasitol. Res..

[102] Vosshall L.B., Hansson B.S. (2011). A unified nomenclature system for the insect olfactory coreceptor. Chem. Senses.

[103] Dippel S., Kollmann M., Oberhofer G., Montino A., Knoll C., Krala M., Rexer K.-H., Frank S., Kumpf R., Schachtner J. (2016). Morphological and transcriptomic analysis of a beetle chemosensory system reveals a gnathal olfactory center. BMC Biol..

[104] Croset V., Rytz R., Cummins S.F., Budd A., Brawand D., Kaessmann H., Gibson T.J., Benton R. (2010). Ancient protostome origin of chemosensory ionotropic glutamate receptors and the evolution of insect taste and olfaction. PLoS Genet..

[105] Rytz R., Croset V., Benton R. (2013). Ionotropic Receptors (IRs): Chemosensory ionotropic glutamate receptors in *Drosophila* and beyond. Insect Biochem. Mol. Biol..

[106] Sánchez-Alcañiz J.A., Silbering A.F., Croset V., Zappia G., Sivasubramaniam A.K., Abuin L., Sahai S.Y., Münch D., Steck K., Auer T.O. (2018). An expression atlas of variant ionotropic glutamate receptors identifies a molecular basis of carbonation sensing. Nat. Commun..

[107] Hussain A., Zhang M., Üçpunar H., Svensson T., Quillery E., Gompel N., Ignell R., Kadow I.C.G. (2016). Ionotropic chemosensory receptors mediate the taste and smell of polyamines. PLoS Biol..

[108] Knecht Z.A., Silbering A.F., Cruz J., Yang L., Croset V., Benton R., Garrity P.A. (2017). Ionotropic receptor-dependent moist and dry cells control hygrosensation in *Drosophila*. eLife.

[109] Pauchet Y., Wilkinson P., Chauhan R., Ffrench-Constant R. (2010). Diversity of beetle genes encoding novel plant cell wall degrading enzymes. PLoS ONE.

[110] Consortium T.G.S., Richards S., Gibbs R.A., Weinstock G.M., Brown S.J., Denell R. (2008). The genome of the model beetle and pest *Tribolium castaneum*. Nature.

[111] Da Lage J.-L. (2018). The amylases of insects. Int. J. Insect Sci..

[112] Da Costa-Latgé S.G., Bates P., Dillon R., Genta F.A. (2021). Characterization of glycoside hydrolase families 13 and 31 reveals expansion and diversification of α-amylase genes in the phlebotomine Lutzomyia longipalpis and modulation of sandfly glycosidase activities by leishmania infection. Front. Physiol..

[113] Keeling C.I., Henderson H., Li M., Yuen M., Clark E.L., Fraser J.D., Huber D.P., Liao N.Y., Docking T.R., Birol I. (2012). Transcriptome and full-length cDNA resources for the mountain pine beetle, *Dendroctonus ponderosae* Hopkins, a major insect pest of pine forests. Insect Biochem. Mol. Biol..

[114] Sicker D., Frey M., Schulz M., Gierl A. (2000). Role of natural benzoxazinones in the survival strategy of plants. Int. Rev. Cytol..

[115] Hanhineva K., Rogachev I., Aura A.-M., Aharoni A., Poutanen K., Mykkänen H. (2011). Qualitative characterization of benzoxazinoid derivatives in whole grain rye and wheat by LC-MS metabolite profiling. J. Agric. Food Chem..

[116] Robert C.A., Zhang X., Machado R.A., Schirmer S., Lori M., Mateo P., Erb M., Gershenzon J. (2017). Sequestration and activation of plant toxins protect the western corn rootworm from enemies at multiple trophic levels. eLife.

[117] Davison A., Blaxter M. (2005). Ancient origin of glycosyl hydrolase family 9 cellulase genes. Mol. Biol. Evol..

[118] Lo N., Tokuda G., Watanabe H., Rose H., Slaytor M., Maekawa K., Bandi C., Noda H. (2000). Evidence from multiple gene sequences indicates that termites evolved from wood-feeding cockroaches. Curr. Biol..

[119] Zhou X., Kovaleva E.S., Wu-Scharf D., Campbell J.H., Buchman G.W., Boucias D.G., Scharf M.E. (2010). Production and characterization of a recombinant beta-1,4-endoglucanase (glycohydrolase family 9) from the termite *Reticulitermes flavipes*. Arch. Insect Biochem. Physiol..

[120] Shelomi M., Watanabe H., Arakawa G. (2014). Endogenous cellulase enzymes in the stick insect (Phasmatodea) gut. J. Insect Physiol..

[121] Rawlings N.D., Barrett A.J., Thomas P.D., Huang X., Bateman A., Finn R.D. (2018). The MEROPS database of proteolytic enzymes, their substrates and inhibitors in 2017 and a comparison with peptidases in the PANTHER database. Nucleic Acids Res..

[122] Cunningham D.F., O’Connor B. (1997). Proline specific peptidases. Biochim. Biophys. Acta BBA—Protein Struct. Mol. Enzym..

[123] Dunaevsky Y.E., Tereshchenkova V.F., Oppert B., Belozersky M.A., Filippova I.Y., Elpidina E.N. (2020). Human proline specific peptidases: A comprehensive analysis. Biochim. Biophys. Acta BBA—Gen. Subj..

[124] Shewry P.R., Halford N.G. (2002). Cereal seed storage proteins: Structures, properties and role in grain utilization. J. Exp. Bot..

[125] Shewry P.R., Tatham A.S. (1990). The prolamin storage proteins of cereal seeds: Structure and evolution. Biochem. J..

[126] Goptar I.A., Filippova I.Y., Lysogorskaya E.N., Oksenoit E.S., Vinokurov K.S., Zhuzhikov D.P., Bulushova N.V., Zalunin I.A., Dunaevsky Y.E., Belozersky M.A. (2008). Localization of post-proline cleaving peptidases in *Tenebrio molitor* larval midgut. Biochimie.

[127] Kakimoto T., Oshima G., Yeh H., Erdös E. (1973). Purification of lysosomal prolylcarboxypeptidase angiotensinase C. Biochim. Biophys. Acta BBA—Enzym..

[128] Odya C., Marinkovic D., Hammon K., Stewart T., Erdös E. (1978). Purification and properties of prolylcarboxypeptidase (angiotensinase C) from human kidney. J. Biol. Chem..

[129] Tan F., Morris P., Skidgel R., Erdös E. (1993). Sequencing and cloning of human prolylcarboxypeptidase (angiotensinase C). Similarity to both serine carboxypeptidase and prolylendopeptidase families. J. Biol. Chem..

[130] Tereshchenkova V.F., Goptar I.A., Kulemzina I.A., Zhuzhikov D.P., Serebryakova M., Belozersky M.A., Dunaevsky Y.E., Oppert B., Filippova I.Y., Elpidina E.N. (2016). Dipeptidyl peptidase 4—An important digestive peptidase in *Tenebrio molitor* larvae. Insect Biochem. Mol. Biol..

[131] Di Cera E. (2009). Serine proteases. IUBMB Life.

[132] Srinivasan A., Giri A.P., Gupta V.S. (2006). Structural and functional diversities in lepidopteran serine proteases. Cell. Mol. Biol. Lett..

[133] Konarev A.V., Fomicheva Y.V. (1991). Cross analysis of the interaction of alpha-amylase and proteinase components of insects with protein inhibitors from wheat endosperm. Biokhimiya.

[134] Zhu Y.-C., Baker J.E. (1999). Characterization of midgut trypsin-like enzymes and three trypsinogen cDNAs from the lesser grain borer, *Rhyzopertha dominica* (Coleoptera: Bostrichidae). Insect Biochem. Mol. Biol..

[135] Zhu Y.-C., Baker J.E. (2000). Molecular cloning and characterization of a midgut chymotrypsin-like enzyme from the lesser grain borer, *Rhyzopertha dominica*. Arch. Insect Biochem. Physiol..

[136] Miao Z., Cao X., Jiang H. (2020). Digestion-related proteins in the tobacco hornworm, *Manduca sexta*. Insect Biochem. Mol. Biol..

[137] Cao X., Jiang H. (2018). Building a platform for predicting functions of serine protease-related proteins in *Drosophila melanogaster* and other insects. Insect Biochem. Mol. Biol..

[138] Agre P. (2004). Aquaporin water channels. Biosci. Rep..

[139] Verkman A. (2013). Aquaporins. Curr. Biol..

[140] Finn R.N., Cerdà J. (2015). Evolution and functional diversity of aquaporins. Biol. Bull..

[141] Fu D., Libson A., Miercke L.J.W., Weitzman C., Nollert P., Krucinski J., Stroud R.M. (2000). Structure of a glycerol-conducting channel and the basis for its selectivity. Science.

[142] Sui H., Han B.-G., Lee J.K., Walian P.J., Jap B.K. (2001). Structural basis of water-specific transport through the AQP1 water channel. Nature.

[143] Beitz E., Wu B., Holm L.M., Schultz J.E., Zeuthen T. (2006). Point mutations in the aromatic/arginine region in aquaporin 1 allow passage of urea, glycerol, ammonia, and protons. Proc. Natl. Acad. Sci. USA.

[144] Fu D., Lu M. (2007). The structural basis of water permeation and proton exclusion in aquaporins (review). Mol. Membr. Biol..

[145] Almasalmeh A., Krenc D., Wu B., Beitz E. (2014). Structural determinants of the hydrogen peroxide permeability of aquaporins. FEBS J..

[146] Finn R.N., Chauvigné F., Stavang J.A., Belles X., Cerdà J. (2015). Insect glycerol transporters evolved by functional co-option and gene replacement. Nat. Commun..

[147] Stavang J.A., Chauvigné F., Kongshaug H., Cerdà J., Nilsen F., Finn R.N. (2015). Phylogenomic and functional analyses of salmon lice aquaporins uncover the molecular diversity of the superfamily in Arthropoda. BMC Genom..

[148] Morishita Y., Matsuzaki T., Hara-Chikuma M., Andoo A., Shimono M., Matsuki A., Kobayashi K., Ikeda M., Yamamoto T., Verkman A. (2005). Disruption of aquaporin-11 produces polycystic kidneys following vacuolization of the proximal tubule. Mol. Cell. Biol..

[149] Gorelick A.D., Praetorius J., Tsunenari T., Nielsen S., Agre P. (2006). Aquaporin-11: A channel protein lacking apparent transport function expressed in brain. BMC Biochem..

[150] Drake L.L., Boudko D.Y., Marinotti O., Carpenter V.K., Dawe A.L., Hansen I.A. (2010). The aquaporin gene family of the yellow fever mosquito, *Aedes aegypti*. PLoS ONE.

[151] Fabrick J.A., Pei J., Hull J.J., Yool A.J. (2014). Molecular and functional characterization of multiple aquaporin water channel proteins from the western tarnished plant bug, *Lygus hesperus*. Insect Biochem. Mol. Biol..

[152] Van Ekert E., Chauvigné F., Finn R.N., Mathew L.G., Hull J.J., Cerdà J., Fabrick J.A. (2016). Molecular and functional characterization of *Bemisia tabaci* aquaporins reveals the water channel diversity of hemipteran insects. Insect Biochem. Mol. Biol..

[153] Yao X.-X., Meng Q.-W., Li G.-Q. (2018). RNA interference-mediated functional characterization of aquaporin genes in *Tribolium castaneum*. Insect Mol. Biol..

[154] Midboe E.G., Candas M., Bulla L.A. (2003). Expression of a midgut-specific cadherin BT-R1 during the development of *Manduca sexta* larva. Comp. Biochem. Physiol. Part B Biochem. Mol. Biol..

[155] Fabrick J.A., Wu Y., Wu Y., Soberón M., Gao A., Bravo A. (2015). Roles of insect midgut cadherin in Bt intoxication and resistance. Bt Resistance: Characterization and Strategies for GM Crops Producing Bacillus Thuringiensis Toxins.

[156] Adang M.J., Crickmore N., Jurat-Fuentes J.L. (2014). Diversity of *Bacillus thuringiensis* crystal toxins and mechanism of action. Adv. Insect Physiol..

[157] Dorsch J., Candas M., Griko N., Maaty W., Midboe E., Vadlamudi R., Bulla L. (2002). Cry1A toxins of *Bacillus thuringiensis* bind specifically to a region adjacent to the membrane-proximal extracellular domain of BT-R1 in Manduca sexta: Involvement of a cadherin in the entomopathogenicity of *Bacillus thuringiensis*. Insect Biochem. Mol. Biol..

[158] Hua G., Jurat-Fuentes J.L., Adang M.J. (2004). Bt-R1a extracellular cadherin repeat 12 mediates *Bacillus thuringiensis* Cry1Ab binding and cytotoxicity. J. Biol. Chem..

[159] Gómez I., Lopez L.P., Muñoz-Garay C., Fernandez L., Pérez C., Sánchez J., Soberón M., Bravo A. (2007). Role of receptor interaction in the mode of action of insecticidal Cry and Cyt toxins produced by *Bacillus thuringiensis*. Peptides.

[160] Sayed A., Nekl E.R., Siqueira H., Wang H.-C., Ffrench-Constant R., Bagley M., Siegfried B.D. (2007). A novel cadherin-like gene from western corn rootworm, Diabrotica virgifera virgifera (Coleoptera: Chrysomelidae), larval midgut tissue. Insect Mol. Biol..

[161] Fabrick J., Oppert C., Lorenzen M.D., Morris K., Oppert B., Jurat-Fuentes J.L. (2009). A novel tenebrio molitor cadherin is a functional receptor for *Bacillus thuringiensis* Cry3Aa toxin. J. Biol. Chem..

[162] Hua G., Park Y., Adang M.J. (2014). Cadherin AdCad1 in *Alphitobius diaperinus* larvae is a receptor of Cry3Bb toxin from *Bacillus thuringiensis*. Insect Biochem. Mol. Biol..

[163] Bel Y., Escriche B. (2006). Common genomic structure for the Lepidoptera cadherin-like genes. Gene.

[164] Petersen M., Armisén D., Gibbs R.A., Hering L., Khila A., Mayer G., Richards S., Niehuis O., Misof B. (2019). Diversity and evolution of the transposable element repertoire in arthropods with particular reference to insects. BMC Ecol. Evol..

[165] Gómez I., Oltean D.I., Gill S.S., Bravo A., Soberón M. (2001). Mapping the epitope in cadherin-like receptors involved in *Bacillus thuringiensis* Cry1A toxin interaction using phage display. J. Biol. Chem..

[166] Whalon M.E., Miller D.L., Hollingworth R.M., Grafius E.J., Miller J.R. (1993). Selection of a Colorado potato beetle (Coleoptera: Chrysomelidae) strain resistant to *Bacillus thuringiensis*. J. Econ. Entomol..

[167] Oppert B., Morgan T.D., Kramer K.J. (2011). Efficacy of *Bacillus thuringiensis* Cry3Aa protoxin and protease inhibitors against coleopteran storage pests. Pest Manag. Sci..

[168] Turk V., Stoka V., Vasiljeva O., Renko M., Sun T., Turk B., Turk D. (2012). Cysteine cathepsins: From structure, function and regulation to new frontiers. Biochim. Biophys. Acta BBA—Proteins Proteom..

[169] Terra W.R., Ferreira C. (1994). Insect digestive enzymes: Properties, compartmentalization and function. Comp. Biochem. Physiol. Part B Comp. Biochem..

[170] Wolfson J.L., Murdock L.L. (1990). Diversity in digestive proteinase activity among insects. J. Chem. Ecol..

[171] Terra W.R., Cristofoletti P. (1996). Midgut proteinases in three divergent species of Coleoptera. Comp. Biochem. Physiol. Part B Biochem. Mol. Biol..

[172] Goptar I., Semashko T., Danilenko S., Lysogorskaya E., Oksenoit E., Zhuzhikov D., Belozersky M., Dunaevsky Y., Oppert B., Filippova I. (2012). Cysteine digestive peptidases function as post-glutamine cleaving enzymes in tenebrionid stored-product pests. Comp. Biochem. Physiol. Part B Biochem. Mol. Biol..

[173] Martynov A.G., Elpidina E.N., Perkin L., Oppert B. (2015). Functional analysis of C1 family cysteine peptidases in the larval gut of *Tenebrio molitor* and *Tribolium castaneum*. BMC Genom..

[174] Perkin L., Elpidina E.N., Oppert B. (2016). Expression patterns of cysteine peptidase genes across the *Tribolium castaneum* life cycle provide clues to biological function. PeerJ.

[175] Oppert B., Elpidina E.N., Toutges M., Mazumdar-Leighton S. (2010). Microarray analysis reveals strategies of *Tribolium castaneum* larvae to compensate for cysteine and serine protease inhibitors. Comp. Biochem. Physiol. Part D Genom. Proteom..

[176] Novinec M., Lenarčič B. (2013). Papain-like peptidases: Structure, function, and evolution. Biomol. Concepts.

[177] Wex T., Lipyansky A., Brömme N.C., Wex H., Guan X.Q., Brömme D. (2001). TIN-ag-RP, a novel catalytically inactive cathepsin B-related protein with EGF domains, is predominantly expressed in vascular smooth muscle cells. Biochemistry.

[178] Schlipalius D., Tuck A.G., Jagadeesan R., Nguyen T., Kaur R., Subramanian S., Barrero R., Nayak M., Ebert P.R. (2018). Variant linkage analysis using de novo transcriptome sequencing identifies a conserved phosphine resistance gene in insects. Genetics.

[179] Wu C., Chakrabarty S., Jin M., Liu K., Xiao Y. (2019). Insect ATP-Binding Cassette (ABC) transporters: Roles in xenobiotic detoxification and Bt insecticidal activity. Int. J. Mol. Sci..

[180] Montella I.R., Schama R., Valle D. (2012). The classification of esterases: An important gene family involved in insecticide resistance—A review. Memórias Inst. Oswaldo Cruz.

[181] Ahn S.-J., Vogel H., Heckel D.G. (2012). Comparative analysis of the UDP-glycosyltransferase multigene family in insects. Insect Biochem. Mol. Biol..

[182] Abdel-Latief M. (2007). A family of chemoreceptors in *Tribolium castaneum* (Tenebrionidae: Coleoptera). PLoS ONE.

[183] Engsontia P., Sangket U., Robertson H.M., Satasook C. (2015). Diversification of the ant odorant receptor gene family and positive selection on candidate cuticular hydrocarbon receptors. BMC Res. Notes.

[184] Biebl S., Querner P. (2021). Transportation of wood boring beetles in wooden transport boxes, wooden pallets, and newly bought wood in museums. Stud. Conserv..

[185] Six D.L. (2020). A major symbiont shift supports a major niche shift in a clade of tree-killing bark beetles. Ecol. Entomol..

[186] Konarev A., Dolgikh V., Senderskiy I., Konarev A., Kapustkina A., Lovegrove A. (2019). Characterisation of proteolytic enzymes of *Eurygaster integriceps* Put. (Sunn bug), a major pest of cereals. J. Asia-Pac. Entomol..

[187] McKenna D.D., Wild A.L., Kanda K., Bellamy C.L., Beutel R.G., Caterino M.S., Farnum C.W., Hawks D.C., Ivie M.A., Jameson M.L. (2015). The beetle tree of life reveals that Coleoptera survived end-Permian mass extinction to diversify during the Cretaceous terrestrial revolution. Syst. Entomol..

[188] Kaur R., Daniels E.V., Nayak M.K., Ebert P.R., Schlipalius I.D. (2013). Determining changes in the distribution and abundance of aRhyzopertha dominicaphosphine resistance allele in farm grain storages using a DNA marker. Pest Manag. Sci..

[189] Schlipalius D.I., Tuck A.G., Pavic H., Daglish G.J., Nayak M.K., Ebert P.R. (2019). A high-throughput system used to determine frequency and distribution of phosphine resistance across large geographical regions. Pest Manag. Sci..

[190] Nayak M.K., Jagadeesan R., Singarayan V.T., Nath N.S., Pavic H., Dembowski B., Daglish G.J., Schlipalius D.I., Ebert P.R. (2021). First report of strong phosphine resistance in stored grain insects in a far northern tropical region of Australia, combining conventional and genetic diagnostics. J. Stored Prod. Res..

[191] Henikoff S., Ahmad K., Malik H.S. (2001). The centromere paradox: Stable inheritance with rapidly evolving DNA. Science.

[192] Heslop-Harrison J.S., Schwarzacher T. (2013). Nucleosomes and centromeric DNA packaging. Proc. Natl. Acad. Sci. USA.

[193] Ugarkovic D., Podnar M., Plohl M. (1996). Satellite DNA of the red flour beetle *Tribolium castaneum*—Comparative study of satellites from the genus *Tribolium*. Mol. Biol. Evol..

[194] Wang S., Lorenzen M., Beeman R.W., Brown S.J. (2008). Analysis of repetitive DNA distribution patterns in the *Tribolium castaneum* genome. Genome Biol..

[195] Saito N., Nei M. (1987). The neighbor-joining method: A new method for reconstructing phylogenetic trees. Mol. Biol. Evol..

[196] Felsenstein J. (1985). Confidence limits on phylogenies: An approach using the bootstrap. Evolution.

[197] Schwarz R., Dayhoff M., Dayhoff M. (1979). Matrices for detecting distant relationships. Atlas of Protein Sequences.

