# Supplementary material for: The Genome of Rhyzopertha dominica (Fab.) (Coleoptera: Bostrichidae): Adaptation for Success
Source: Genes (Basel). 2022 Feb 28;13(3):446. doi: 10.3390/genes13030446 (PMC8956072; doi:10.3390/genes13030446)
Supplement: Supplementary file 1 [file genes-13-00446-s001.zip › genes-1558055-suppl-final-revised/Figure S2 ABC transporters.pdf]

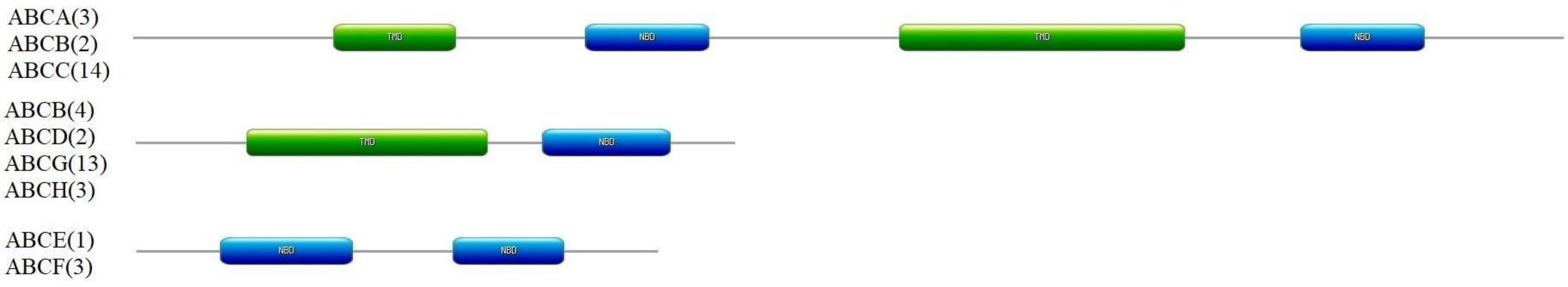

Supplementary Figure S2. Conserved domain analysis of the ABC transporters of *R. dominica*. The numbers in parantheses indicate the number of ABC transporters in each subfamily. TMD: transmembrane domain; NBD: nucleotide binding domain.
