# Supplementary material for: The Genome of Rhyzopertha dominica (Fab.) (Coleoptera: Bostrichidae): Adaptation for Success
Source: Genes (Basel). 2022 Feb 28;13(3):446. doi: 10.3390/genes13030446 (PMC8956072; doi:10.3390/genes13030446)
Supplement: Supplementary file 1 [file genes-13-00446-s001.zip › genes-1558055-suppl-final-revised/Figure S3.pdf]

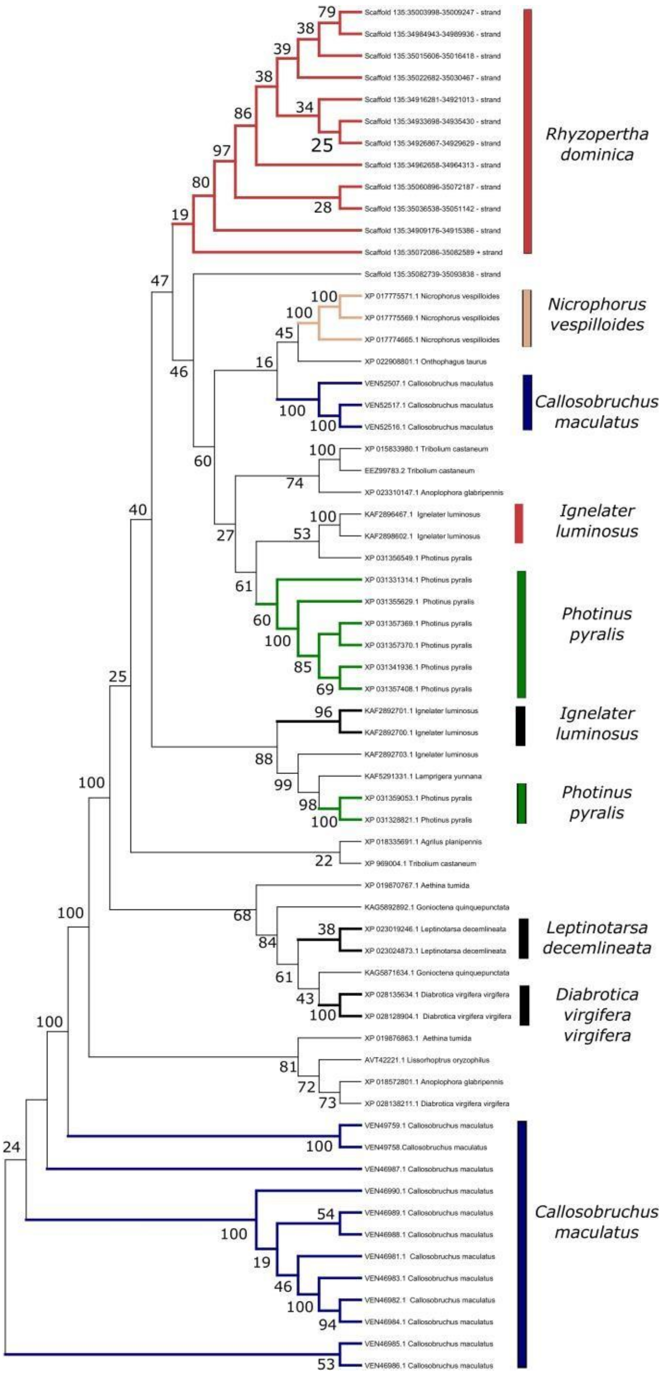

Supplementary Figure S3. Phylogenetic comparison of UGT sequences found on scaffold 135 of the *R. dominica* genome and grouping within a single clade and those found in other clades (*Nicrophorus vespilloides, Callosobruchus maculatus, Ignelater luminosus, Photinus pyralis, L. decemlineata* and *D. virgifera virgifera*).
