# Supplementary material for: The Genome of Rhyzopertha dominica (Fab.) (Coleoptera: Bostrichidae): Adaptation for Success
Source: Genes (Basel). 2022 Feb 28;13(3):446. doi: 10.3390/genes13030446 (PMC8956072; doi:10.3390/genes13030446)
Supplement: Supplementary file 1 [file genes-13-00446-s001.zip › genes-1558055-suppl-final-revised/Figure S5.pdf]

```
GAGCTGTACCATCGCCATTTTTATATCGTCTTTTGTTTTAAAACTAGCCAACTTTTTAATAATGTAAC 68
ATTTCTTCAGTTCTCAATATCTCGATCGAGTATGTAGATACAGAAAAACTGTAGACATTTTTATAGGA 136
TATGGACAAAGCAATTCGCTTTTATTTATATGGTTTAATAATAGCTCTTTTTACCTTCAAGGGTGTAC 204
  M  D  K  A  I  R  F  Y  L  Y  G  L  I  I  A  L  F  T  F  K  G  V   22

ATAGCTGGAGCTTCATACATTCCGATGACGACAGAGTTGACACAACTGACGTCATCACTACAAATAAT 272
H  S  W  S  F  I  H  S  D  D  D  R  V  D  T  T  D  V  I  T  T  N  N  45
                                             EC1          *
ATCCTAGTATATATGGATGAAGAACTGGAACCTAATATTATACTAGCATACGTGAATTATGATGGGTC 340
 I  L  V  Y  M  D  E  E  L  E  P  N  I  I  L  A  Y  V  N  Y  D  G  S 68

GTCAACACCAGAAATAGCACAGATTAATCCAGACAACAGTATTATAGGAGCAGTATTACAAGAAGAAT 408
  S  T  P  E  I  A  Q  I  N  P  D  N  S  I  I  G  A  V  L  Q  E  E   90

CACTTGAAGGCGAATCTTATTGGGCAGTTGTAATAACTAACAAAATTGATTATGAATCCATCCCAGAA 476
S  L  E  G  E  S  Y  W  A  V  V  I  T  N  K  I  D  Y  E  S  I  P  E  113

GATGTGATGGTGTTCGAAATAGAAATATCAGTTGAAGGTGACAGCAATACAATATTTCTTTACGTACA 544
 D  V  M  V  F  E  I  E  I  S  V  E  G  D  S  N  T  I  F  L  Y  V  Q 136

GAACATAGATGATAATGCACCAATAATAGTTGTTGTTACAAACCCATGCCAAATTGAGGAAAACTACG 612
  N  I  D  D  N  A  P  I  I  V  V  V  T  N  P  C  Q  I  E  E  N  Y   158
                                                  EC2
CTGGTCCTTCCAACTGTACATACAACATACAAGATGCTGATGGATGGCTACAATATATGAAAATGGAT 680
A  G  P  S  N  C  T  Y  N  I  Q  D  A  D  G  W  L  Q  Y  M  K  M  D  181
            *
GTTTCTGGAACAAGAAACGAAACGGAACTCTTCAAATTTGAATACGAAGATCTAAATGAGATAACTAA 748
 V  S  G  T  R  N  E  T  E  L  F  K  F  E  Y  E  D  L  N  E  I  T  N 204
                                                                     *
TACAACAAAATCAGCATCATTGCTGCTACATACTATAGCAGAACTGGACTATGAAACAATAACTCTCC 816
  T  T  K  S  A  S  L  L  L  H  T  I  A  E  L  D  Y  E  T  I  T  L   226

ATCTGATTAACTGGACGGTAACGGACAGCGGCAATCATTCTGTATCACAAACGGTGGTTGTAAATGTT 884
H  L  I  N  W  T  V  T  D  S  G  N  H  S  V  S  Q  T  V  V  V  N  V  249
         *
ATTGACCTACCTGATACTCCACCTAGATGGACGACAATTTTTGCAACTCAACAGTTTGACGAAAAAAC 952
 I  D  L  P  D  T  P  P  R  W  T  T  I  F  A  T  Q  Q  F  D  E  K  T 272

CGAACAGCACTTTTCCGTTGCAGCTATAGACGGAGACATTGGAGTAAATGACGATATAAAATATTCCT 1020
  E  Q  H  F  S  V  A  A  I  D  G  D  I  G  V  N  D  D  I  K  Y  S   294
      EC3
TAGAGTCTGAATATGACGATCTGTTTTCTATCGACAGCACTACAGGTGACCTCTATGTTAATGAAATT 1088
L  E  S  E  Y  D  D  L  F  S  I  D  S  T  T  G  D  L  Y  V  N  E  I  317

GACCGTGATACATTGAACATGCAAATTTTTACATTTCTTATATTTGCAACAGAGGAAAATGACCCATG 1156
 D  R  D  T  L  N  M  Q  I  F  T  F  L  I  F  A  T  E  E  N  D  P  W 340

GTATTCTACTAATCAAACCATCACATTTGTTGTAAATGATGTAAATGATCATGCTCCTAATGTAACAG 1224
  Y  S  T  N  Q  T  I  T  F  V  V  N  D  V  N  D  H  A  P  N  V  T   362
                                                            *
ATCCTGATCCAAAACAAATACACTTTTTACTGTTAGAAGAAAGGGTAACGCAACTGAATGATACGATT 1292
D  P  D  P  K  Q  I  H  F  L  L  L  E  E  R  V  T  Q  L  N  D  T  I  385
                                                  EC4
TCTGTACTAGATAGAGACTCGATAGAAAACGCCATGTTTACTATATCTTTGCAAGATACATCCGATAA 1360
 S  V  L  D  R  D  S  I  E  N  A  M  F  T  I  S  L  Q  D  T  S  D  N 408
```

```
TGGCATTTTGTACACCGAGGCTTTTCAAGTTCCTTCGCAAGGGTATCAGAATTACACGTTTTCTTTAT 1428
  G  I  L  Y  T  E  A  F  Q  V  P  S  Q  G  Y  Q  N  Y  T  F  S  L   430
                                                  *
CAATCATCGACAGTAGCCTCTTAGACTATGATGATGAAAACTGGCAAAATTTTAATTTTAAAGTTTTC 1496
S  I  I  D  S  S  L  L  D  Y  D  D  E  N  W  Q  N  F  N  F  K  V  F   452

ATAGAAGAAACGGTAGACCCATCGCATACTGATGAAGTCGAAATTACCGTAAATCTAATAAACTGGAA 1564
 I  E  E  T  V  D  P  S  H  T  D  E  V  E  I  T  V  N  L  I  N  W  N 476

TGACGAAACTCCCATATTTGAAAATGAGGAATATGATGCCGAAATTTATGAAAACGTCAGCATACATA 1632
  D  E  T  P  I  F  E  N  E  E  Y  D  A  E  I  Y  E  N  V  S  I  H   498
             EC5   --------->                          *
CCTTCATCCAACAGGTATATGCAACCGACAGAGACGTGGACGACGAAATTAGGTACAGTCTAGTAGGA 1700
T  F  I  Q  Q  V  Y  A  T  D  R  D  V  D  D  E  I  R  Y  S  L  V  G   521

GCATCGGTAAATACAATTCTTGCGGTCAATTCAAGTGGGATTGTTTCGACGACTGTTGACGGAGCATT 1768
 A  S  V  N  T  I  L  A  V  N  S  S  G  I  V  S  T  T  V  D  G  A  F 544
                            *
TGATTACGAGCGTCAAACAGAATACATTATTCAAATTGCTGCACAAGACACGCTGCAAGTTATAAACG 1836
  D  Y  E  R  Q  T  E  Y  I  I  Q  I  A  A  Q  D  T  L  Q  V  I  N   566

AATCTCTCCATACAGCATACGCTCAACTGACGATAAATATTCTGGATGTCAACGATGAAACGCCATCG 1904
E  S  L  H  T  A  Y  A  Q  L  T  I  N  I  L  D  V  N  D  E  T  P  S   589

ATCAGAGTTGAATCACTAATAAGTGTTGAAGAAAACAGGCCAAATGGCACAGAGATAGATTCTACTAT 1972
 I  R  V  E  S  L  I  S  V  E  E  N  R  P  N  G  T  E  I  D  S  T  I 612
    EC6  ---------->                          *
TACAGCAACCGATGATGACACTACAGCCGAGTTATTATTCTATATAGATTGGGATTCAACTTACGCTA 2040
  T  A  T  D  D  D  T  T  A  E  L  L  F  Y  I  D  W  D  S  T  Y  A   634

CAAAAAGTGGGCGAATGTATGATGACCTTCTAGAGTACTATAGAGACTGCATACTGGTAGAAACCGAA 2108
T  K  S  G  R  M  Y  D  D  L  L  E  Y  Y  R  D  C  I  L  V  E  T  E   657

GACATTTCAGACACATCATCAGGTTATGCTAAAGCAAAGCTTTTTATAAACGATGTGGTGGAAGGAAA 2176
 D  I  S  D  T  S  S  G  Y  A  K  A  K  L  F  I  N  D  V  V  E  G  N 680

CACCCCTGATTATGAAAAATTTGATACCTTATACATAAGACTAATAGTAGAAGACCTGAATCAGGAAG 2244
  T  P  D  Y  E  K  F  D  T  L  Y  I  R  L  I  V  E  D  L  N  Q  E   702

AAGGAGAAGCTACTGCTGCAGTTACTGTTACAATTGTGGTTGAGGACCTGAATGACAATTATCCAGTT 2312
E  G  E  A  T  A  A  V  T  V  T  I  V  V  E  D  L  N  D  N  Y  P  V   725

TTTACGGACAGTGTTAATACAAATAATACAGTCACAGAAGCTGCGGTTCGTGGTGTTACTATAGGAAC 2380
 F  T  D  S  V  N  T  N  N  T  V  T  E  A  A  V  R  G  V  T  I  G  T 748
       EC7  ---------->                                    *
CATTACTGCAACAGATGATGACGGACCAGGCTTCAATACTGTAACATATTCCATTGAGCCAGCAGGCA 2448
  I  T  A  T  D  D  D  G  P  G  F  N  T  V  T  Y  S  I  E  P  A  G   770

GTACTCCGCAAGGACTGGTCACGATTAATTCAACAACTGGACTACTCACTGTCGATGAGGATGGTGCA 2516
S  T  P  Q  G  L  V  T  I  N  S  T  T  G  L  L  T  V  D  E  D  G  A   793
```

ATAGATTGTGATAACCCACCTATATATTACCTTAACTATACGGTGATTGCTACGGATGGTGAACTAGA 2584

I D C D N P P I Y Y L N Y T V I A T D G E L D 816

*

TAGCACTGCAGAAATCCAGATTTATGTCTTTGACATAAACGACAATGCACCCATCATCGAAAGTTATG 2652

S T A E I Q I Y V F D I N D N A P I I E S Y 838

TBR1

AGGACACAATAGAAATACCTGAGAAATCCGCCAATGGTACCAATGTTGTAGAAATAATAGCTATTGAT 2720

E D T I E I P E K S A N G T N V V E I I A I D 861

EC8 *

GCCGACCGTGATAGTCCTTACAATGTCGTCTGGTATGAATTTACAACAGCATCTAACAGCGATGTAAG 2788

A D R D S P Y N V V W Y E F T T A S N S D V R 884

AAGTTTTTTCTTAATGGATATGGATACAGGAGTTATAAGCGTGCGTTACGTAAGTGCAGAATCGACTC 2856

S F F L M D M D T G V I S V R Y V S A E S T 906

TTGACAGAGACTATGGTGTTAGTTCATTTACGGTTGCCTTTAAAGCCGCTGATAACTACTACTCAACA 2924

L D R D Y G V S S F T V A F K A A D N Y Y S T 929

GGTGGCATACAAAGGAGTGCAGAAGGCGTATTTACGATTATTTTAACCGATATTAATGATAAGGTCCC 2992

G G I Q R S A E G V F T I I L T D I N D K V P 952

TGTTGTTACCACCAAATACATCAGTGTATCAGAAAATACATCTAATAATACTCAAATAAGCACTCGCA 3060

V V T T K Y I S V S E N T S N N T Q I S T R 974

EC9 *

TTGAAGCCTATGATGACGACGAGGAAGGAACTGACAATTCAAGAATTAACTTTGCAGTCAAAGGAATG 3128

I E A Y D D D E E G T D N S R I N F A V K G M 997

ACAGACGAAGAAGGTGCTGAATATCAGTTATTTGAGATAGTAAATGACGAAGATGAGAACGGTGTAGC 3196

T D E E G A E Y Q L F E I V N D E D E N G V A 1020

ATACCTTTCCACGCTTGTTGATTTAGAGGGTTATTATGGCAACTATACTTTGATCATTCAGGTGAACG 3264

Y L S T L V D L E G Y Y G N Y T L I I Q V N 1042

ATTTTGGAGAACCTATGAATACTGAAAATTATAGTGTAACCTTGGAAATTGAAAAGTATAGTTACTAT 3332

D F G E P M N T E N Y S V T L E I E K Y S Y Y 1065

GCACCAGACTATCTATACCCTGAAAATCCTGACGGTGAACGAGTGTACCTTAGAAAGACCCAAAACGT 3400

A P D Y L Y P E N P D G E R V Y L R K T Q N V 1088

TAACGGACAACTATACTTGTACGATGGACGTGAACTACCAAATATTCAAGCTACCGATAACCAAGGTG 3468

N G Q L Y L Y D G R E L P N I Q A T D N Q G 1110

EC10

AAAAATGGGATATTACTTTTACAATCGACTCTGATGATTCAGATGAGGCTATGATTTTCCAAATCGAC 3536

E K W D I T F T I D S D D S D E A M I F Q I D 1133

AATTCAGATTCTCTGGGTCAATCAACAGGAATGCTGCAATTAACTACAATAGAAAACTGGGAAGATCA 3604

N S D S L G Q S T G M L Q L T T I E N W E D Q 1156

ATATACCTTGAATATAATAGCAAATGATAACGGAACTCCACAACGCACTGCGAATGCTGTAATGTATA 3672

Y T L N I I A N D N G T P Q R T A N A V M Y 1178

TTCGATTCTTTGATTTAAATTCATTGCCATATTTTCCGGAAAATGAAAGGCAACTAACAGCGCAGTTC 3740

I R F F D L N S L P Y F P E N E R Q L T A Q F 1201

EC11

```
ACAGAAAATACAACAGGAATGTCTGAGAGTGTTTCCATCCCAGGTGCTTCATATAGTGGCACTGAAGA 3808
 T  E  N  T  T  G  M  S  E  S  V  S  I  P  G  A  S  Y  S  G  T  E  D 1224

CGAAGACTTTGATGTGTACTATTTATTGCTTGAAGGAAACAGCAGCATTTTTAATATCGATATTAATA 3876
  E  D  F  D  V  Y  Y  L  L  L  E  G  N  S  S  I  F  N  I  D  I  N  1246

CTGGACTAATTACCCTTTTGGAAATGTTAGACAGAGAAGAAACAGCATCACACGATATTACAATAATT 3944
T  G  L  I  T  L  L  E  M  L  D  R  E  E  T  A  S  H  D  I  T  I  I  1269

GCATCTAACAGTGATTCTGTTCCAACCAGTTATGATGAACAGTCCTTGCTTTTCGTTACTATTAAGGT 4012
 A  S  N  S  D  S  V  P  T  S  Y  D  E  Q  S  L  L  F  V  T  I  K  V 1292
    *                        TBR2
AATTGATGTAAATGACAATCCACCACAATTTAGCGAAAGTCGGTACACTGGAGGAATAAGTACTTCGA 4080
  I  D  V  N  D  N  P  P  Q  F  S  E  S  R  Y  T  G  G  I  S  T  S  1314
                                  EC12
GTTCTTTGGATTATACTGTGACTACAGTTTCAGCGTACGATCCAGATATTAACGATACCTTAACTTAC 4148
S  S  L  D  Y  T  V  T  T  V  S  A  Y  D  P  D  I  N  D  T  L  T  Y  1337

TCAATTAACACATCAAGTTTAACCGTTAGTGATTCATCGCTCTCCAACATTGCTACACCTTTCAGAAT 4216
 S  I  N  T  S  S  L  T  V  S  D  S  S  L  S  N  I  A  T  P  F  R  I 1360

TGATTCATCATCTGGTGCGGTAACATTAAACTTCGCAGTTCAAAGCAACATGCTTGGATATTTTGCGT 4284
  D  S  S  S  G  A  V  T  L  N  F  A  V  Q  S  N  M  L  G  Y  F  A  1382
                                 TBR3
TCAATGTTGTTGTAACTGACCCACCTCCATATCATACAGATACAGCACTCGTAGAGATTTATATAATC 4352
F  N  V  V  V  T  D  P  P  P  Y  H  T  D  T  A  L  V  E  I  Y  I  I  1405

ACGGATGAACATAGGGTGGAGTTTACTTTCCAGAATGATCTGAACACTGTAATAGCTCTTCAGTCCGA 4420
 T  D  E  H  R  V  E  F  T  F  Q  N  D  L  N  T  V  I  A  L  Q  S  E 1428

GGTAAAAGATGTATTAGATGACGCGTTTGAATATTTATGCAACATTGAATCTATTCTTAGTGATACGG 4488
  V  K  D  V  L  D  D  A  F  E  Y  L  C  N  I  E  S  I  L  S  D  T  1450

ATGATGATGGCCAAACTTTGGATACTCAAACTGTATGTACTGTACATTTTATTGATCCTGAATTAAAT 4556
D  D  D  G  Q  T  L  D  T  Q  T  V  C  T  V  H  F  I  D  P  E  L  N  1473

GAACCAGTTAATAAAACTACAATACAGAGCAAACTTAGTAGTCTAACATTCTATTCAAATCTGCGAAG 4624
 E  P  V  N  K  T  T  I  Q  S  K  L  S  S  L  T  F  Y  S  N  L  R  S 1496

CTCCTTCAGCAATATTACATTGTATCTTTTGAGTTACTCAAGTGGATCTACAACAACAGATAATCTTG 4692
  S  F  S  N  I  T  L  Y  L  L  S  Y  S  S  G  S  T  T  T  D  N  L  1518
                                             *
AAGATCAATTAAAAGCTTGGCTAATAGGTGTGACTGTTGTACTAGGATCTCTCTGTATTACACTCCTA 4760
E  D  Q  L  K  A  W  L  I  G  V  T  V  V  L  G  S  L  C  I  T  L  L  1541

GTTGGATTTATCATCAAGCTACAAACATTGAAACGACGCTTGAATAAGTTATCAGAAGTTAAATTTGG 4828
 V  G  F  I  I  K  L  Q  T  L  K  R  R  L  N  K  L  S  E  V  K  F  G 1564

TTCTCAAGAGTCTGGTTTAAACCGCATTGGTGTAGCTGTTCCAAATACTAACAAAAATGCAGTTGAAG 4896
  S  Q  E  S  G  L  N  R  I  G  V  A  V  P  N  T  N  K  N  A  V  E  1586

GTTCTAATCCAGTTTATAATGAGGAAAAGAAAATTCCAGATTTTGATCGATCAAGTATTCATAGTGGA 4964
G  S  N  P  V  Y  N  E  E  K  K  I  P  D  F  D  R  S  S  I  H  S  G  1609
```

```
GACTCCGACCTTATTGGTGTTGAAGACAATCCAGAATTTGACTATGGCATTAAAAATGAAAGATACTG 5032
 D  S  D  L  I  G  V  E  D  N  P  E  F  D  Y  G  I  K  N  E  R  Y    1631

ACAAAGTGAATCATATATAGGTAATATAAGGTTTTAGCTGGCAAAGAAATTTGAGAGCATATAAATAA 5100
ATAAATAAATAAATAAATGT                                                 5120
```

Supplementary Figure S5. cDNA sequence and deduced open reading frame of the *R. dominica* BtR1-like cadherin gene (*RdCad1*). The predicted domains are underlined: the membrane signal peptide with a dashed line, the MPED domain with a solid line, and CYT domain with a dotted line. The start of each of the twelve cadherin repeat domains is marked with a solid arrow. The TM domain region is shaded gray. Predicted N-and O-glycosylation sites are marked by asterisks. The putative toxin binding regions (TBR1-3) are designated by the rounded rectangle below the sequence.
