# Supplementary material for: The Genome of Rhyzopertha dominica (Fab.) (Coleoptera: Bostrichidae): Adaptation for Success
Source: Genes (Basel). 2022 Feb 28;13(3):446. doi: 10.3390/genes13030446 (PMC8956072; doi:10.3390/genes13030446)
Supplement: Supplementary file 1 [file genes-13-00446-s001.zip › genes-1558055-suppl-final-revised/Figure S6.pdf]

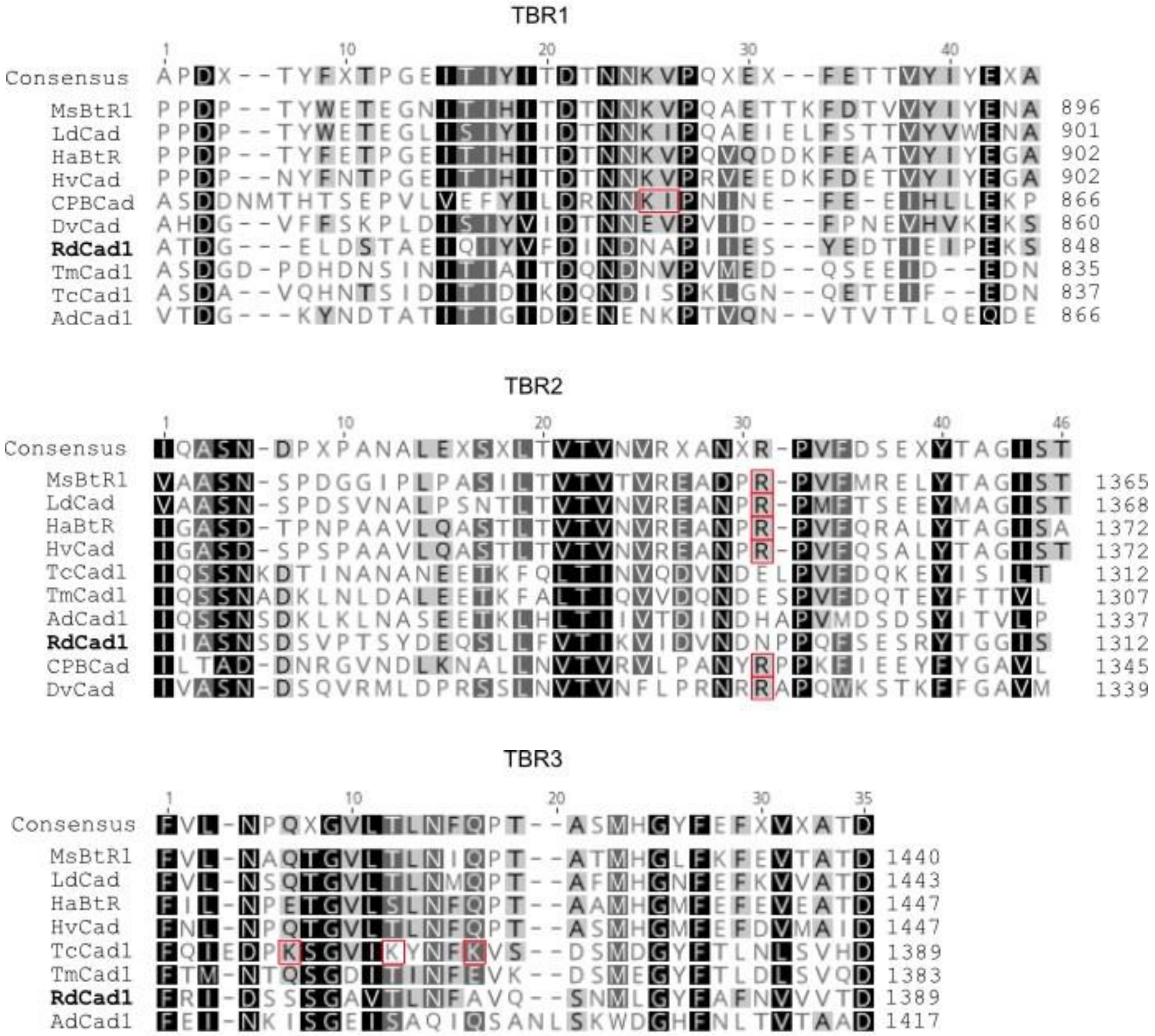

Supplementary Figure S6. Alignment of the putative toxin binding regions (TBR 1; 2 and 3) of insect midgut cadherins – the Cry toxin receptors. Cadherins include those from *M. sexta* (MsBtR1, AAM21151), *Lymantria dispar* (LdCad, AF317621_1), *Helicoverpa armigera* (HaBtR, ACF94775), *Heliothis virescens* (HvCad, AAV80768), *Tribolium castaneum* (TcCad1, XP_971388), *Tenebrio molitor* (TmCad1, DQ988044), *Alphitobis diaperinus* (AdCad1, AHJ10508) and *R. dominica* (RdCad1). Critical toxin-binding region of EC1-10 cadherin of chrysomelid beetles *Diabrotica virgifera* (DvCad, ABU50692), and the putative Colorado potato beetle (*Leptinotarsa decemlineata*) ortholog (CPBCad, XP_023015891) demonstrate similarity with an epitope of TBR2 and there was no obvious match to TBR3. The TBRs residues that may potentially affect on the Cry toxin interaction are outlined in red boxes (refer to the text for more details).
