# Supplementary material for: The Genome of Rhyzopertha dominica (Fab.) (Coleoptera: Bostrichidae): Adaptation for Success
Source: Genes (Basel). 2022 Feb 28;13(3):446. doi: 10.3390/genes13030446 (PMC8956072; doi:10.3390/genes13030446)
Supplement: Supplementary file 1 [file genes-13-00446-s001.zip › genes-1558055-suppl-final-revised/Figure S7.pdf]

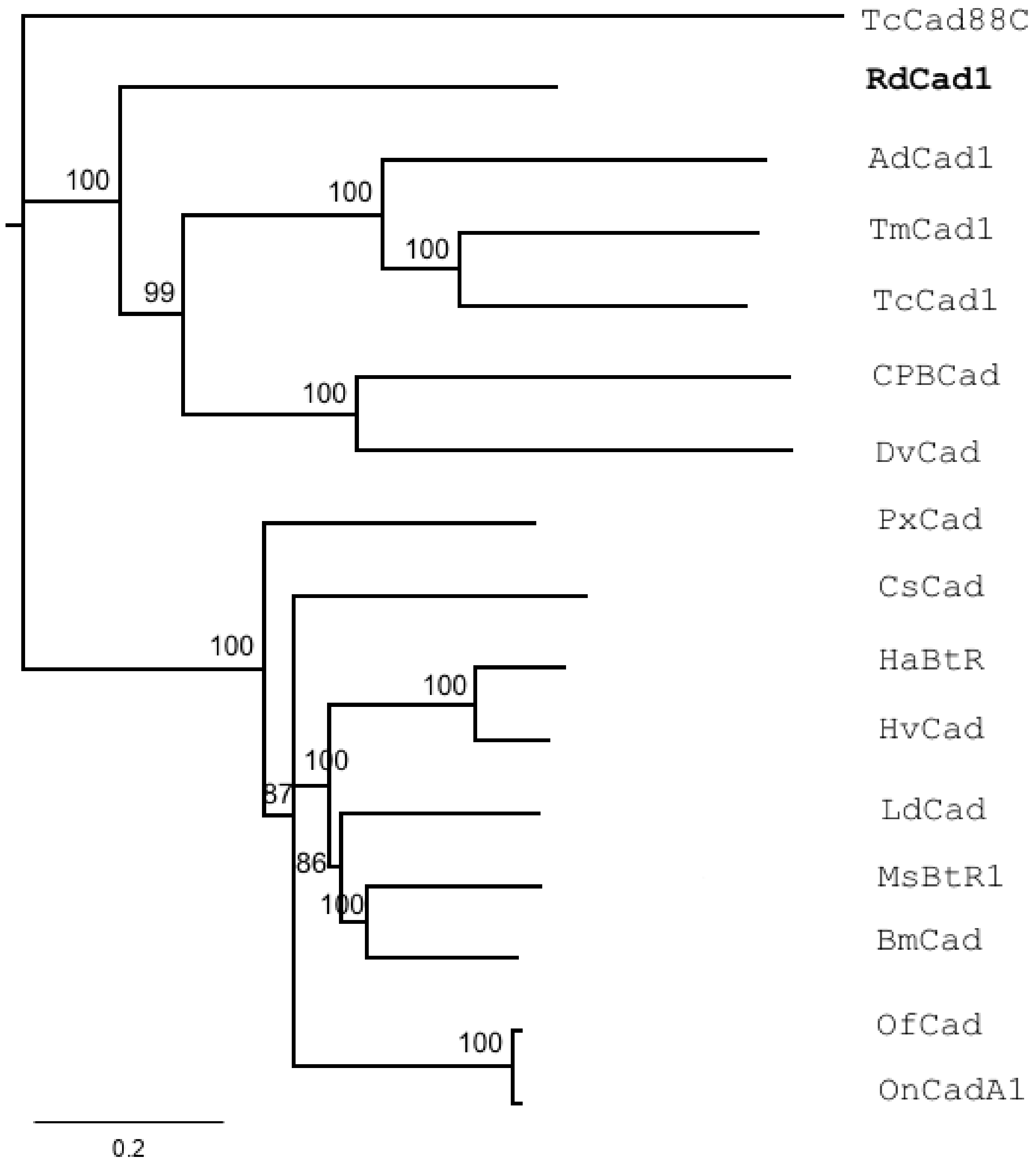

Supplementary Figure S7. Phylogenetic analysis of insect cadherin protein sequences. Phylogenetic data obtained from ClustalW-aligned sequences were processed using Maximum Likelihood tree builder algorithm (MEGA X) with 500 bootstrap iterations. Protein sequences used in the analysis were from *M. sexta* (MsBtR1), *Bombyx mori* (BmCad, BAA99404), *H. virescens* (HvCad), *H. armigera* (HaBtR), *L. dispar* (LdCad), *Ostrinia furnacalis* (OfCad), *O. nubilalis* (OnCadA1, AAT37678), *Plutella xylostella* (PxCad, ABU41413), *Chilo suppressalis* (CsCad, AAM78590), *R. dominica* (RdCad1), *A. diaperinus* (AdCad1); *T. molitor* (TmCad1), *T. castaneum* (TcCad1), *Leptinotarsa decemlineata* (CPBCad), *D. virgifera* (DvCad). The *T. castaneum* cadherin (TcCad88C, XP_971786), was used as an outgroup. The tree was drawn to scale, with branch lengths showing the number of substitutions per site.
