# Supplementary material for: The Genome of Rhyzopertha dominica (Fab.) (Coleoptera: Bostrichidae): Adaptation for Success
Source: Genes (Basel). 2022 Feb 28;13(3):446. doi: 10.3390/genes13030446 (PMC8956072; doi:10.3390/genes13030446)
Supplement: Supplementary file 1 [file genes-13-00446-s001.zip › genes-1558055-suppl-final-revised/Figure S8.pdf]

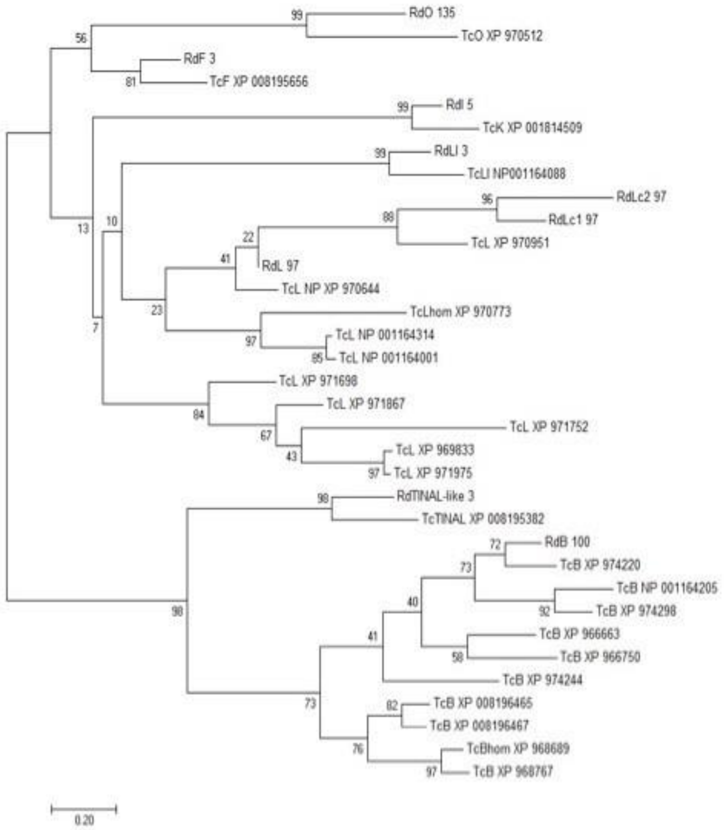

Supplementary Figure S8. Phylogenetic analysis of *R. dominica* and *T. castaneum* cathepsins. Maximum likelihood tree with 500 iterations of bootstrapping were used; bootstrapping values are represented as a percentage (0–100) by each branch. Evolutionary distances are represented by branch length.
