# Supplementary material for: The Genome of Rhyzopertha dominica (Fab.) (Coleoptera: Bostrichidae): Adaptation for Success
Source: Genes (Basel). 2022 Feb 28;13(3):446. doi: 10.3390/genes13030446 (PMC8956072; doi:10.3390/genes13030446)
Supplement: Supplementary file 1 [file genes-13-00446-s001.zip › genes-1558055-suppl-final-revised/Figure S9 Repeat Figures.pdf]

a.

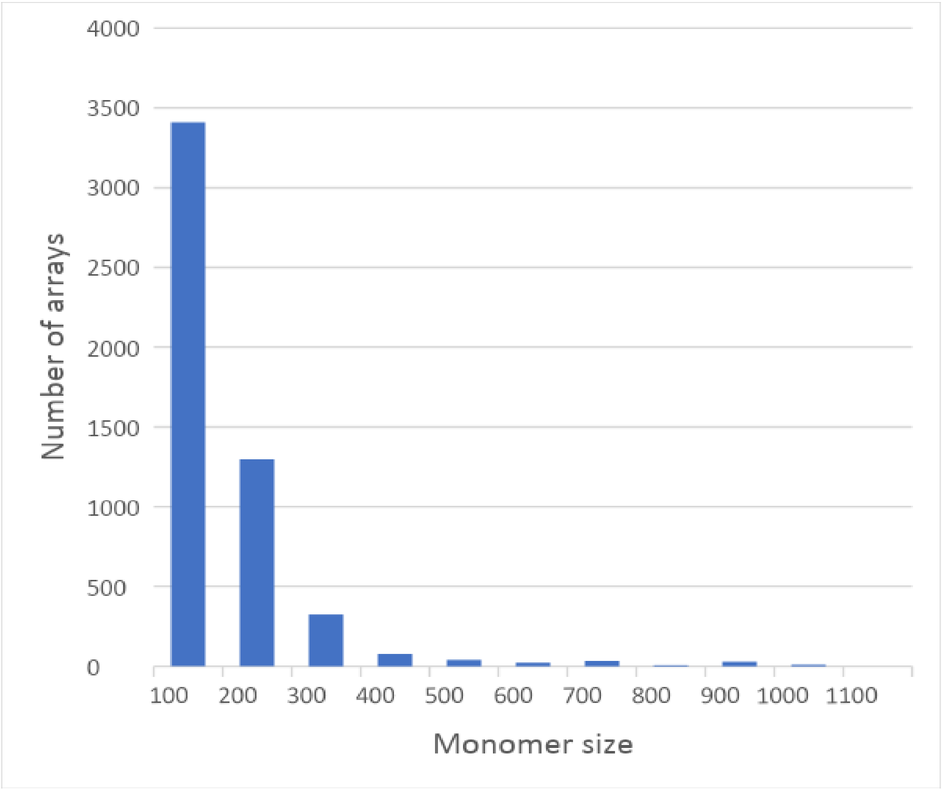

4000
3500
3000
2500
2000
1500
1000
500
0
Number of arrays
100
200
300
400
500
600
700
800
900
1000
1100
Monomer size

b.

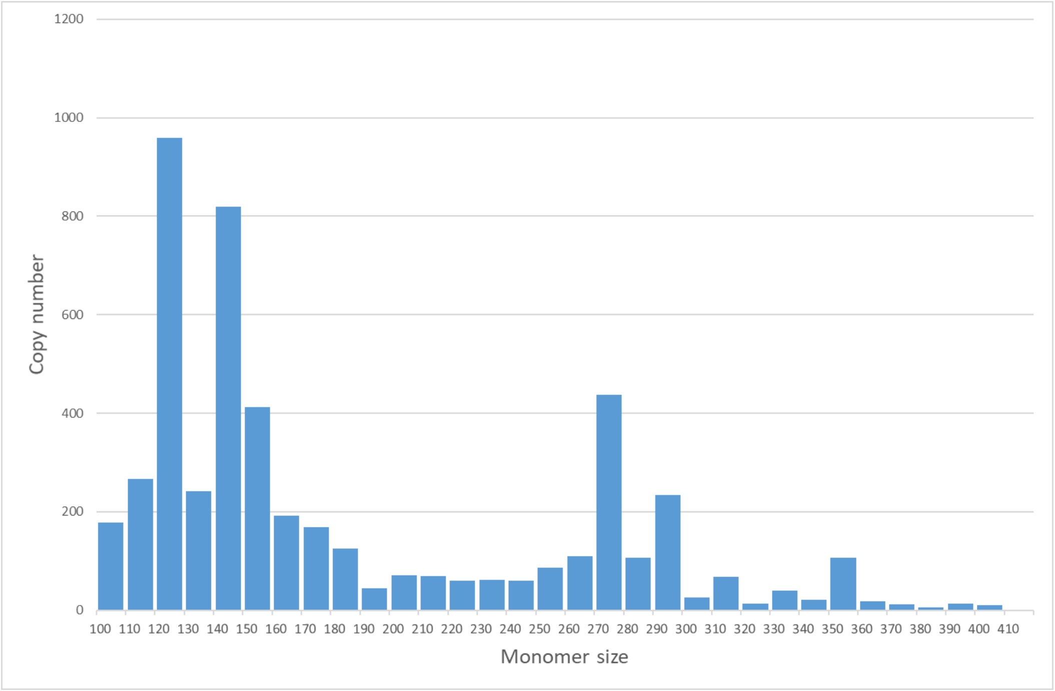

1200
1000
800
600
400
200
0
Copy number
100 110 120 130 140 150 160 170 180 190 200 210 220 230 240 250 260 270 280 290 300 310 320 330 340 350 360 370 380 390 400 410
Monomer size

c.

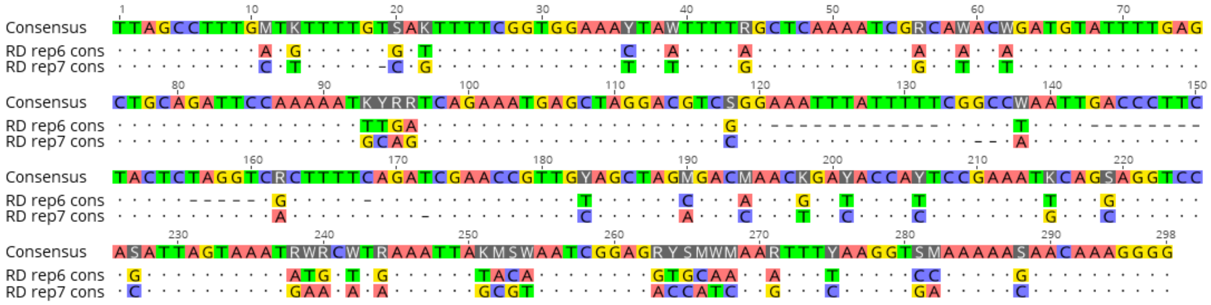

Supplementary Figure S9. Evaluation of monomers in the *R. dominica* genome. (a)**.** Distribution of number of arrays according to the monomer length. Monomer size of up to 2000 bp was examined (shown only up to 1100 bp due to the decreasing abundance). (b). Number of monomers in relation to the monomer size. (c). Alignment of consensus sequences of two related families, RD rep6 and rep7.
