# Supplementary material for: The Genome of Rhyzopertha dominica (Fab.) (Coleoptera: Bostrichidae): Adaptation for Success
Source: Genes (Basel). 2022 Feb 28;13(3):446. doi: 10.3390/genes13030446 (PMC8956072; doi:10.3390/genes13030446)
Supplement: Supplementary file 1 [file genes-13-00446-s001.zip › genes-1558055-suppl-final-revised/File S1 Dissection of LGB larvae.pptx]

## Slide 1
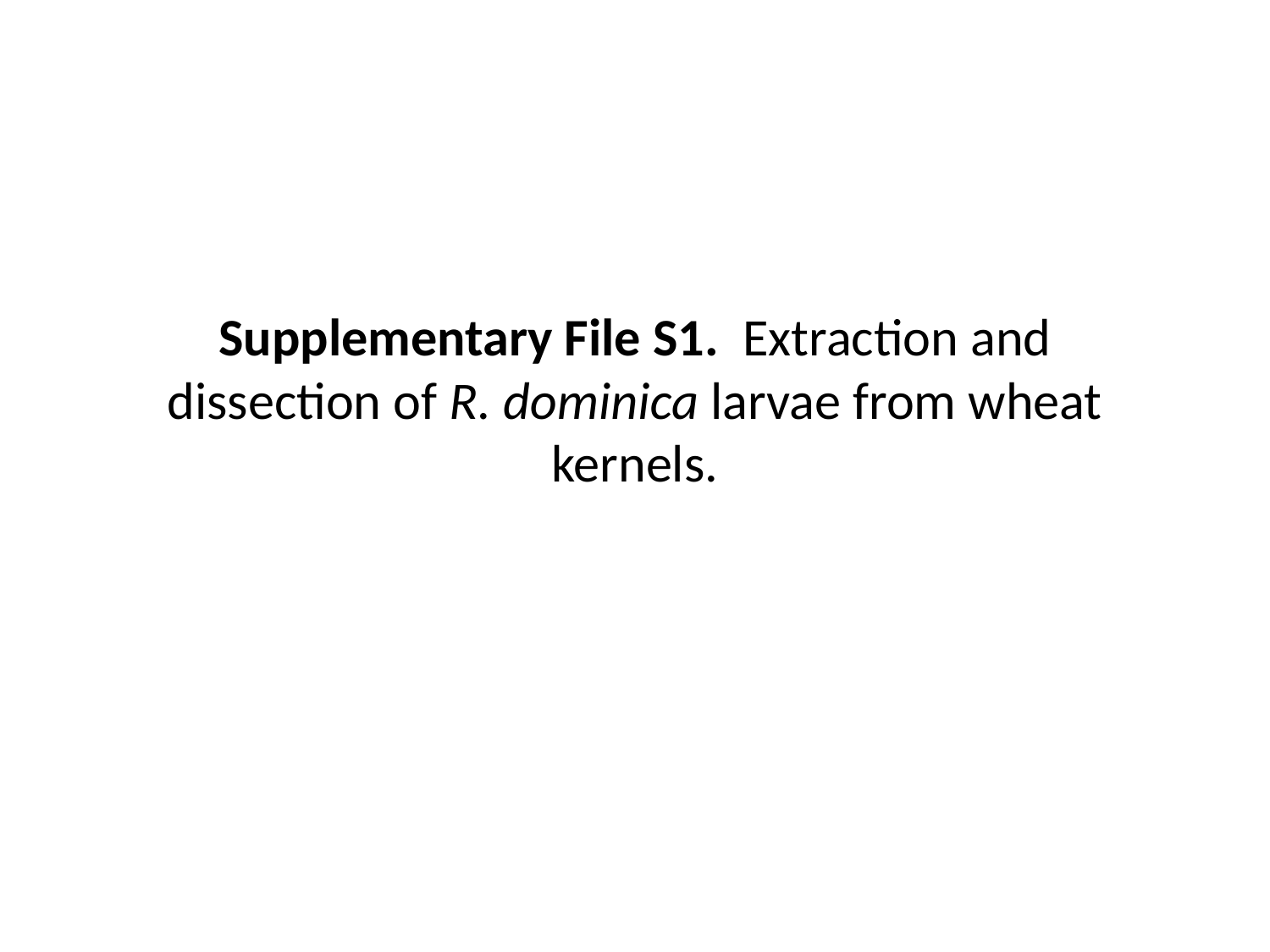

# Supplementary File S1. Extraction and dissection of R. dominica larvae from wheat kernels.

## Slide 2
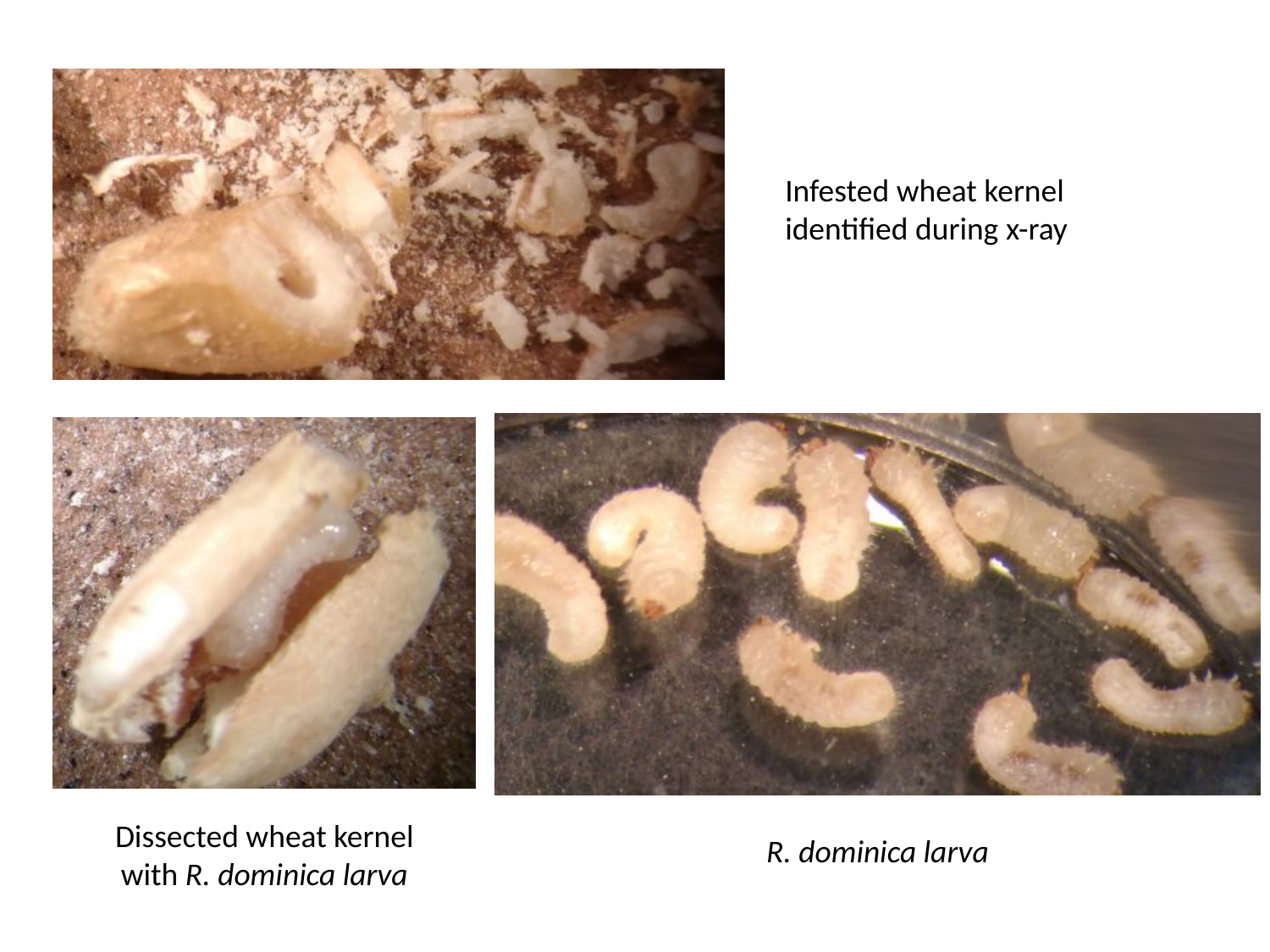

Infested wheat kernel identified during x-ray
Dissected wheat kernel with R. dominica larva
R. dominica larva

## Slide 3
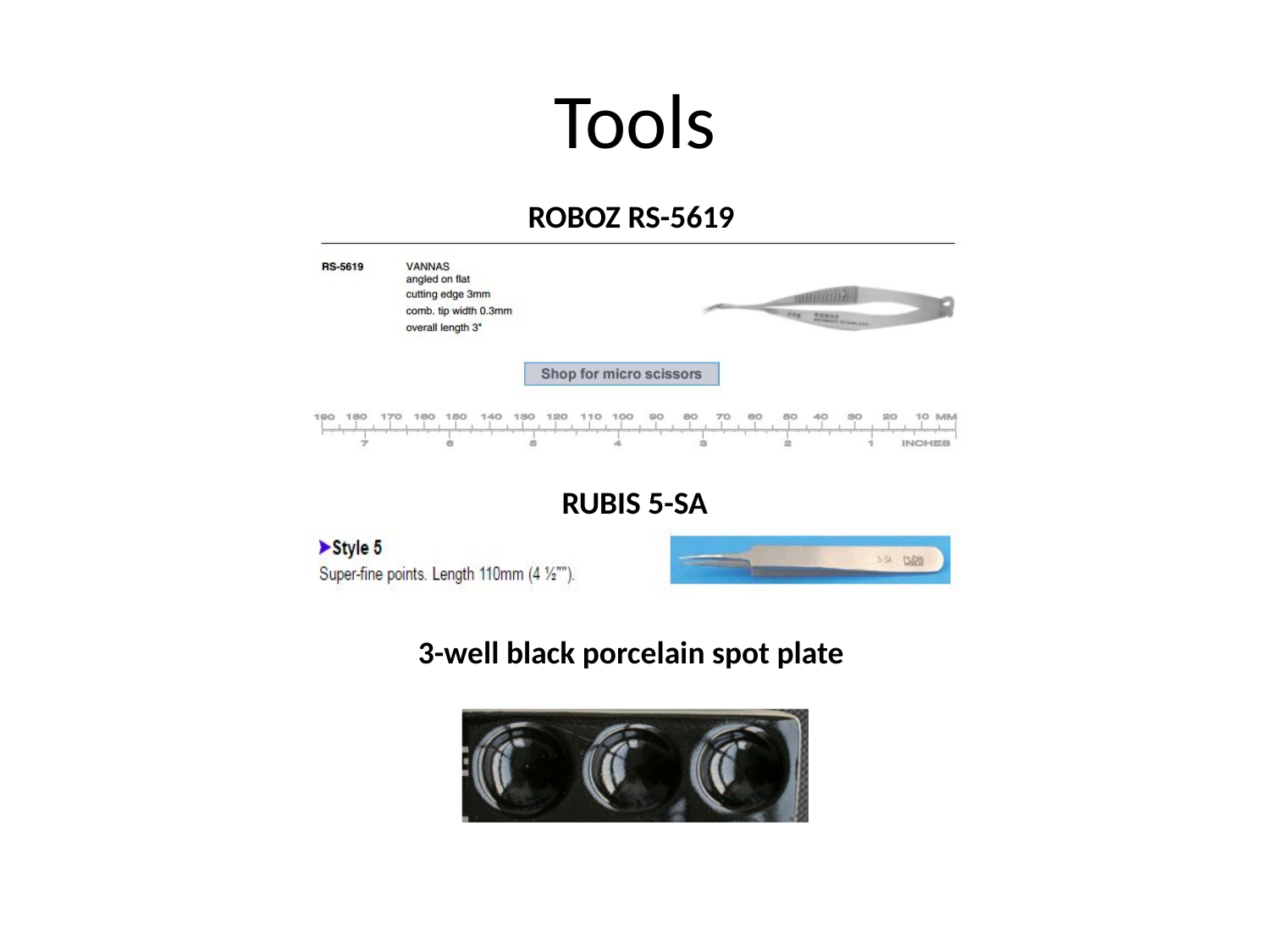

# Tools
ROBOZ RS-5619
RUBIS 5-SA
3-well black porcelain spot plate

## Slide 4
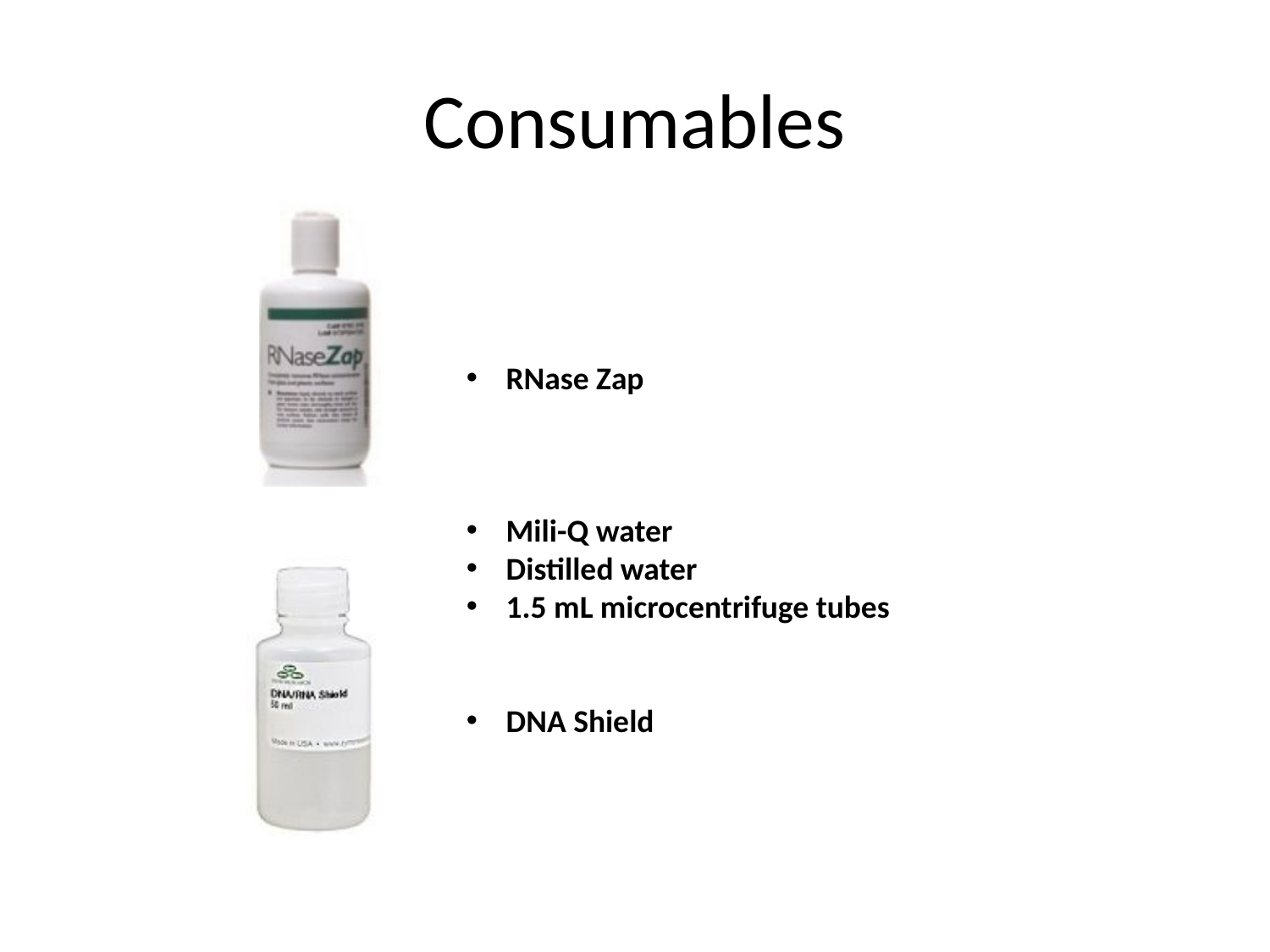

# Consumables
RNase Zap
Mili-Q water
Distilled water
1.5 mL microcentrifuge tubes
DNA Shield

## Slide 5
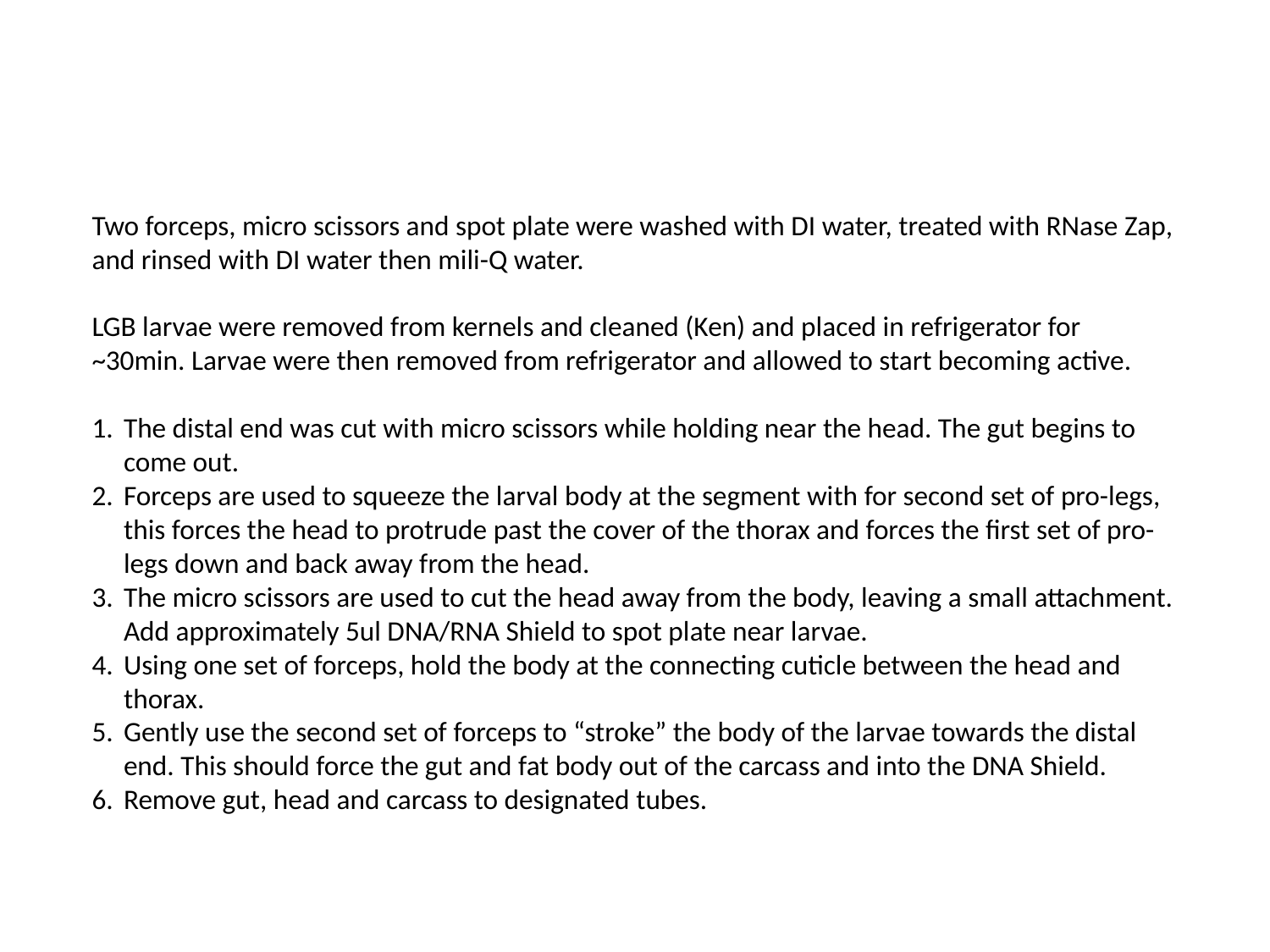

Two forceps, micro scissors and spot plate were washed with DI water, treated with RNase Zap, and rinsed with DI water then mili-Q water.
LGB larvae were removed from kernels and cleaned (Ken) and placed in refrigerator for ~30min. Larvae were then removed from refrigerator and allowed to start becoming active.
The distal end was cut with micro scissors while holding near the head. The gut begins to come out.
Forceps are used to squeeze the larval body at the segment with for second set of pro-legs, this forces the head to protrude past the cover of the thorax and forces the first set of pro-legs down and back away from the head.
The micro scissors are used to cut the head away from the body, leaving a small attachment. Add approximately 5ul DNA/RNA Shield to spot plate near larvae.
Using one set of forceps, hold the body at the connecting cuticle between the head and thorax.
Gently use the second set of forceps to “stroke” the body of the larvae towards the distal end. This should force the gut and fat body out of the carcass and into the DNA Shield.
Remove gut, head and carcass to designated tubes.

## Slide 6
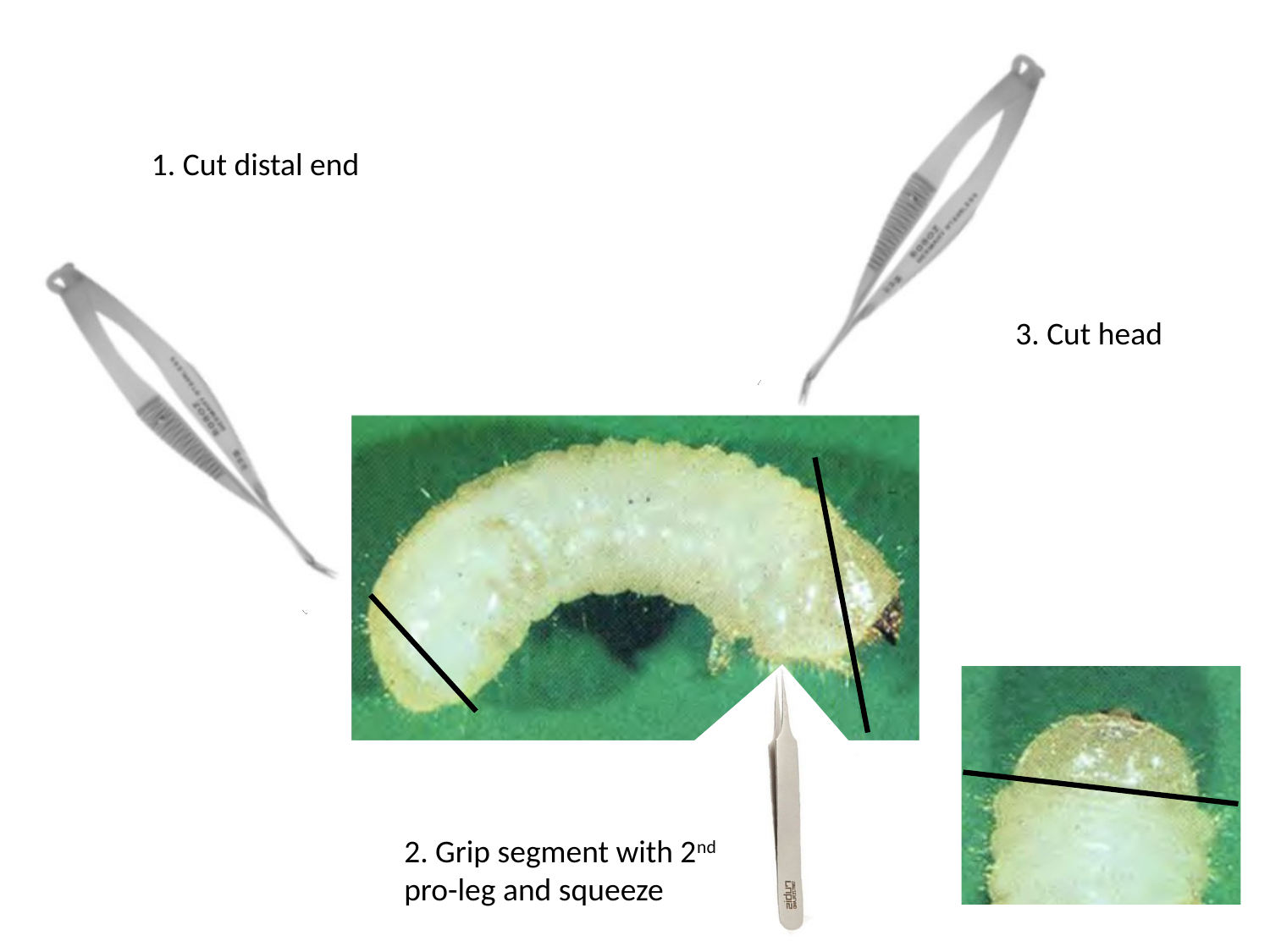

1. Cut distal end
3. Cut head
2. Grip segment with 2nd pro-leg and squeeze

## Slide 7
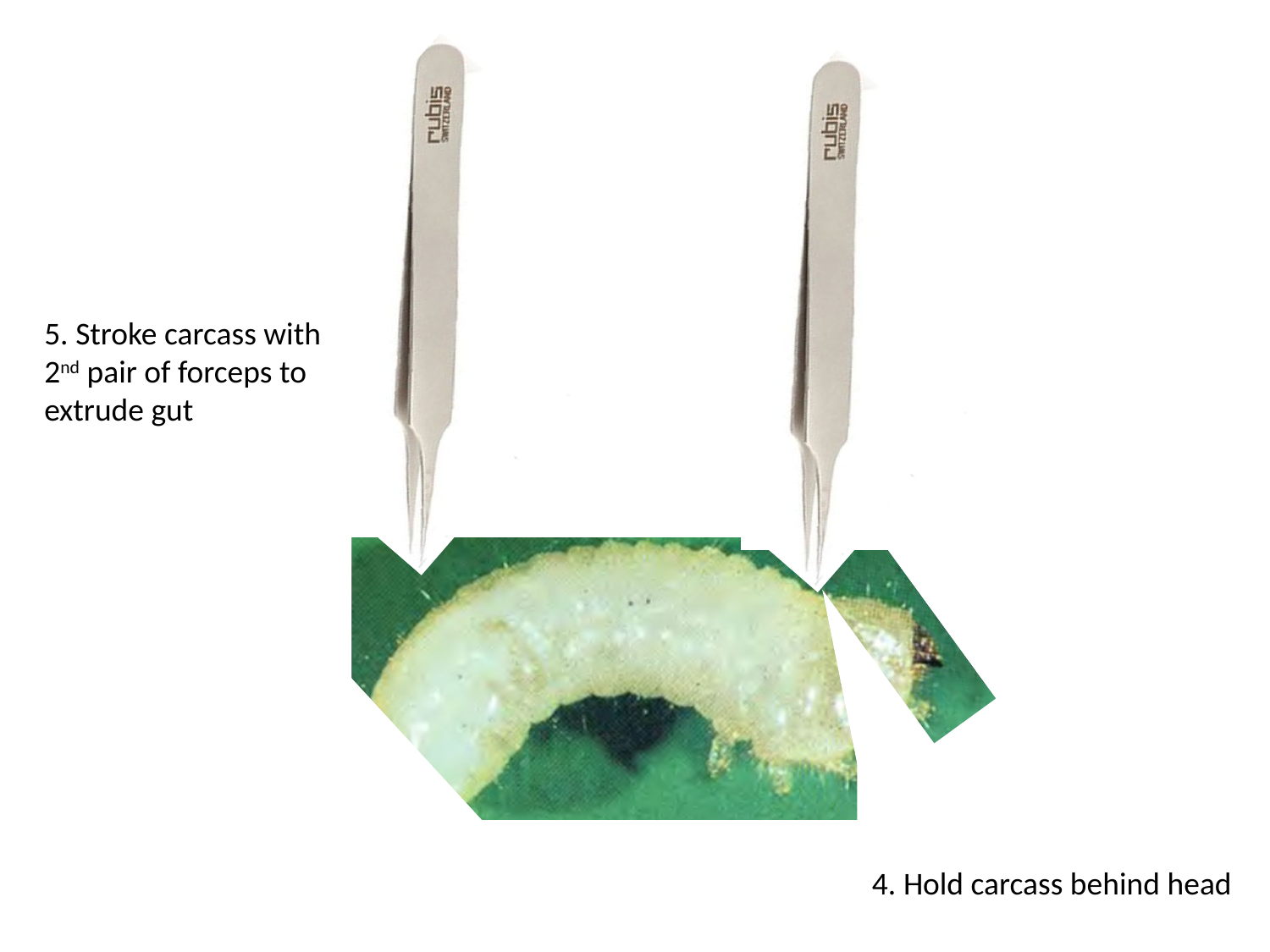

4. Hold carcass behind head
5. Stroke carcass with 2nd pair of forceps to extrude gut
4. Hold carcass behind head
