# Supplementary material for: The Genome of Rhyzopertha dominica (Fab.) (Coleoptera: Bostrichidae): Adaptation for Success
Source: Genes (Basel). 2022 Feb 28;13(3):446. doi: 10.3390/genes13030446 (PMC8956072; doi:10.3390/genes13030446)
Supplement: Supplementary file 1 [file genes-13-00446-s001.zip › genes-1558055-suppl-final-revised/Table S4_BtR1_cadh_genomic_org.pdf]

Supplementary Table S4. Genomic organization of *R. dominica* Bt-R1-like cadherin gene. The number and length of the deduced exons and their encoding cadherin domains are shown. All the intron-exon borders provided were followed the GT-AG rule for RNA 5' acceptor and 3' donor splice sites. The exon sequences are shown in upper case, coding regions for the first and last exons are shown in parentheses, asterisk indicate that the lengths providedfor 5'- and 3'-UTRs were only approximate. The start and stop codon (TGA) were underlined and shaded grey, respectively. The transposable elements(TE) predicted by Censor (www.girinst.org/censor/) inside the introns of cadherin gene are provided.

Exon		Protein domain	Intron/exon junction	Exon/Intron junction	Intron	
№	Size (bp)		5’-Acceptor	3’-Donor	Size (bp)	TE classes
-1 (5'-UTR)	134*			ATTTTTATAG**gt**atacaaat	803	nd
1	67 (64)	SP	tttttttc**ag**GATATGGACA	TTCAAGGGTG**gt**aagtatgt	52	nd
2	135	EC1	aacatgac**ag**TACATAGCTG	AATTATGATG**gt**aagtttat	15 217	LTR/Gypsy; DNA/Transib
3	266	EC1, EC2	aatttttc**ag**GGTCGTCAAC	CCAAATTGAG**gt**aagtaaac	17 337	LTR/Gypsy
4	90	EC2	tttcttgc**ag**GAAAACTACG	TTCTGGAACA**gt**acgtataa	623	nd
5	147	EC2	ttattttc**ag**AGAAACGAAA	GACGGTAACG**gt**aagttaat	23 227	DNA/Mariner; LTR/Gypsy
6	120	EC2, EC3	actttttt**ag**GACAGCGGCA	AACCGAACAG**gt**ttgtataa	51	nd
7	73	EC3	tgtgtttt**ag**CACTTTTCCG	GAGTCTGAAT**gt**aatataca	55	nd
8	281	EC3, EC4	gcgatttc**ag**ATGACGATCT	TAGAGACTCG**gt**aagtattt	2 115	nd
9	371	EC4, EC5	ttcttttc**ag**ATAGAAAACG	ACGAAATTAG**gt**atgtttag	3 821	nd
10	229	EC5	tatctttc**ag**GTACAGTCTA	GATCAGAGTT**gt**aagtaact	3 021	nd
11	173	EC6	atactttt**ag**GAATCACTAA	ACTATAGAGA**gt**aagtacat	15 511	LTR/Bel, LTR/Gypsy
12	179	EC6	tatattgt**ag**CTGCATACTG	ACTGCTGCAG**gt**aacacaat	143	nd
13	172	EC6, EC7	catcttct**ag**TTACTGTTAC	ATTCCATTGA**gt**aagtaact	8 981	LTR/Gypsy
14	160	EC7	taaatttc**ag**GCCAGCAGGC	CACTGCAGAA**gt**aagaagtt	2 402	Non-LTR/RTE
15	136	EC7, EC8	cgttatgc**ag**ATCCAGATTT	GACCGTGATA**gt**aagtatat	590	nd
16	192	EC8	cttttgtt**ag**GTCCTTACAA	TACTCAACAG**gt**aaaatagg	69	nd
17	113	EC8, EC9	tataccct**ag**GTGGCATACA	TACATCTAAT**gt**aagtaaat	357	nd
18	219	EC9	atgttcac**ag**AATACTCAAA	GATCATTCAG**gt**attttagt	87	nd
19	132	EC9, EC10	caattttt**ag**GTGAACGATT	CCTTAGAAAG**gt**aagaagaa	77	nd
20	112	EC10	gctttttt**ag**ACCCAAAACG	ATCGACTCTG**gt**tagtaata	4 647	LTR/Gypsy
21	110	EC10	atttttgt**ag**ATGATTCAGA	TCAATATACC**gt**aagttact	54	nd
22	399	EC10, EC11	atggttgc**ag**TTGAATATAA	TACTATTAAG**gt**aaaacatt	1 129	nd
23	102	EC11, EC12	tacatttt**ag**GTAATTGATG	TACAGTTTCA**gt**aagttttt	54	nd
24	196	EC12	ttgcttta**ag**GCGTACGATC	ACTGACCCAC**gt**aagtaacg	5 106	DNA/Mariner, LTR/Gypsy
25	113	EC12, MPED	tcccttac**ag**CTCCATATCA	TCAGTCCGAG**gt**tcactttt	53	nd
26	164	MPED	tttgtttt**ag**GTAAAAGATG	CAATACAGAG**gt**actaatgc	65	nd

27	201	MPED, TM	atcatttt**ag**CAAACTTAGT	AGCTACAAAC**gt**aagttaac	295	nd
28	165	CYT	aatgttac**ag**ATTGAAACGA	ATCGATCAAG**gt**acgttttt	61	nd
29	270*<br>(82)	CYT	gtgtatac**ag**TATTCATAGT	AAGATACTGACAAAGTGAAT	-	
