# Supplementary material for: The Genome of Rhyzopertha dominica (Fab.) (Coleoptera: Bostrichidae): Adaptation for Success
Source: Genes (Basel). 2022 Feb 28;13(3):446. doi: 10.3390/genes13030446 (PMC8956072; doi:10.3390/genes13030446)
Supplement: Supplementary file 1 [file genes-13-00446-s001.zip › genes-1558055-suppl-final-revised/Table S5 Repeats Table.pdf]

Supplementary Table S5. Composition of repetitive elements in the *R. dominica* genome obtained by RepeatModeler,

Classification	Total	With Domains	% With Domains
DNA	7521	134	1.78
DNA/Academ	163	1	0.61
DNA/CMC	5145	5	0.1
DNA/Crypton	706	50	7.08
DNA/Ginger	220	26	11.82
DNA/hAT	5167	330	6.39
DNA/IS3EU	10	0	0
DNA/Kolobok	39	26	66.67
DNA/Maverick	1458	145	9.95
DNA/Merlin	202	26	12.87
DNA/MULE	2686	245	9.12
DNA/P	1332	165	12.39
DNA/PIF	700	220	31.43
DNA/PiggyBac	441	88	19.95
DNA/Sola	6	0	0
DNA/Sola1	271	53	19.56
DNA/TcMar	117278	6719	5.73
LINE	9840	351	3.57
LINE/CR1	23833	1518	6.37
LINE/Dong	141	39	27.66
LINE/I	3455	206	5.96
LINE/Jockey	5011	484	9.66
LINE/L1	146	57	39.04
LINE/L2	33966	2609	7.68
LINE/LOA	876	109	12.44
LINE/Penelope	46770	1970	4.21
LINE/R1	789	122	15.46
LINE/R2	5	2	40
LINE/RTE	5210	439	8.43
LTR	400	288	72
LTR/Copia	3096	224	7.24
LTR/DIRS	713	87	12.2
LTR/Gypsy	17612	2200	12.49
LTR/Pao	4761	705	14.81
RC/Helitron	443	52	11.74
Retroelement	1772	1772	100
rRNA	29	0	0
Satellite	2	0	0
Simple_repeat	68	0	0
SINE/tRNA	30	0	0
TE	15834	132	0.83
tRNA	1	0	0
