# Supplementary material for: The Genome of Rhyzopertha dominica (Fab.) (Coleoptera: Bostrichidae): Adaptation for Success
Source: Genes (Basel). 2022 Feb 28;13(3):446. doi: 10.3390/genes13030446 (PMC8956072; doi:10.3390/genes13030446)
Supplement: Supplementary file 1 [file genes-13-00446-s001.zip › genes-1558055-suppl-final-revised/Table S6 Repeats.pdf]

**Supplementary Table S6.** Consensus monomer sequences of the 10 most prominent repeat families in the *R. dominica* genome.

| Repeat | Monomer Consensus Sequence |
|---|---|
| RD rep1 | GCTCCAGCTTGCCCAGAGCTAGCTCCAGCTAGCTCCACTAAAGAGAAAGCTAGCTCCACCTTACTTGAGTAGTTATAAGCTCATCATTTCGGGGGGTGCTAACTTAACGTTATCCTCGCCATGGCCTCAAATCTGTTTCAAAACCTA |
| RD rep2 | ATTAGAGACGATGCATTCACTTTAATACGGCATCCTCTTATGCATTCTGGAAACAGTTGCTTCATTATAGCAGATCGGAAGCGAAGACGACCATTAAAAGGCACAATTGGTCAAACTAACAAT |
| RD rep3 | TAATAACGTATTTGGTTAAACTAATACCATAAGAGTTTATTCTTTCCACTTAATACGGCATACTATATACGCTTGCTGGACCCAGCTTCGTCATTACAGCTTATCGTATATAAGGCGACCGTCTAA |
| RD rep4 | CAAGCATAACAGATTGTGTCGCTTTAAATCAAACAATATAGTCCCTTATACTATTATATTGAGCAAAAGCGTTATTAGAAGCTAGACGTATCTTCCTGACTCATATAATACCAGCATTGTACCCAG |
| RD rep5 | GACCGATTTGAATGAATTTTTTTGTATGCGTTTGGGTGGCGCCCTGGATGGTTTAGATTCACAAATCAGCCCGGCAGATGGCGCTGCAGTCGGTATCTAGGTTATTTATCTATACTGCAACTAAACG |
| RD rep6 | CATTCCGAAATTCAGGAGGTCCAGATTAGTAAATATGCTTGAAATTATACAAATCGGAGGTGCAAAAATTTTAAGGTCCAAAAAGAACAAAGGGGTTAGCCTTTGATGTTTTGTGATTTTTCGGTGGAAACTAATTTTAGCTCAAAATCGACAAACAGATGTATTTTGAGCTGCAGATTCCAAAAATTTGATCAGAAATGAGCTAGGACGTCGGGCGGCCTAATTTACTCCGCTTTTAGATCGAACCGTTGTAGCTAGCGACAAACGGATAC |
| RD rep7 | TTAGCCTTTGCTTTTTTGCAGTTTTCGGTGGAAATTATTTTTGGCTCAAAATCGGCATACTGATGTATTTTGAGCTGCAGATTCCAAAAATGCAGTCAGAAATGAGCTAGGACGTCCGGAAATTTATTTTTCGCAAATTGACCCTTCTACTCTAGGTCACTTTTCAGACGAACCGTTGCAGCTAGAGACCAACTGACACCACTCCGAAATGCAGCAGGTCCACATTAGTAAATGAACATAAAATTAGCGTAATCGGAGACCATCAAGTTTCAAGGTGAAAAAACAACAAAGGGG |
| RD rep8 | GGTCAAACAATCGGTTCACAGATTCGGTGAGATGAATTCAAAAAGTTTAAGCACGTTTTATCGCAGCGCAGTGGTTTGACAAATTATAGTTTGTCGTAATTATAATTGGT |
| RD rep9 | AAAAGATGGGCAATTGTGCTTCAATTTGGTGGCTAACTTCCAAATATAAAAATATTGATTTTACTAAAAAGGGGTCATGTGGCTAAGTTAACACATGAAGCACAATGAAGCACAATCGAAGCACTAAAGACTAGT |
| RD rep10 | TCTAATTACGGCAATGAATTGTGCTCGTTTTGTGCTGAAATGTGCCGGTGTCAGGGAGTTTTGTGCAGCAACCCTTATATTTAATTTTTTAAAAACACCGGCGGGCGGGGCCAGCTTGACGGTCAGCCCCGCTTTTTTTGGGAATATTCAAAA |
