# Supplementary figures and images for: The Genome of Rhyzopertha dominica (Fab.) (Coleoptera: Bostrichidae): Adaptation for Success
Source: Genes (Basel). 2022 Feb 28;13(3):446. doi: 10.3390/genes13030446 (PMC8956072; doi:10.3390/genes13030446)

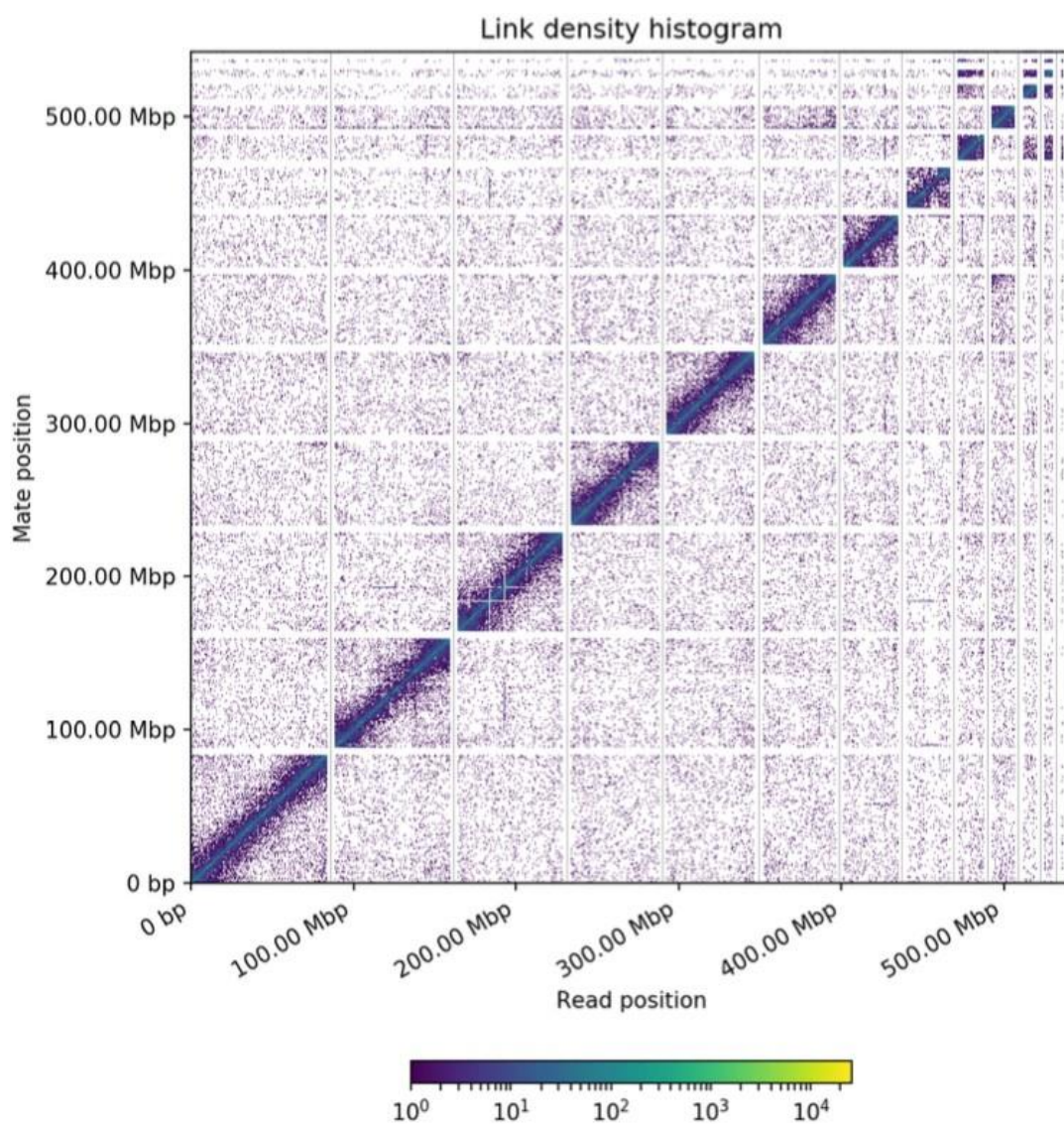

**Supplementary Figure S1.** Link density histogram of Hi-C scaffolded *R. dominica* genome assembly.

Supplement: Supplementary file 1 [file genes-13-00446-s001.zip › genes-1558055-suppl-final-revised/Figure S1 Link Density Histogram.pdf]
