# Supplementary material for: The Genome of Rhyzopertha dominica (Fab.) (Coleoptera: Bostrichidae): Adaptation for Success
Source: Genes (Basel). 2022 Feb 28;13(3):446. doi: 10.3390/genes13030446 (PMC8956072; doi:10.3390/genes13030446)
Supplement: Supplementary file 1 [file Figure S4 Aquaporin.pdf › genes-1558055-suppl-final-revised/Figure S4 Aquaporin.pdf]

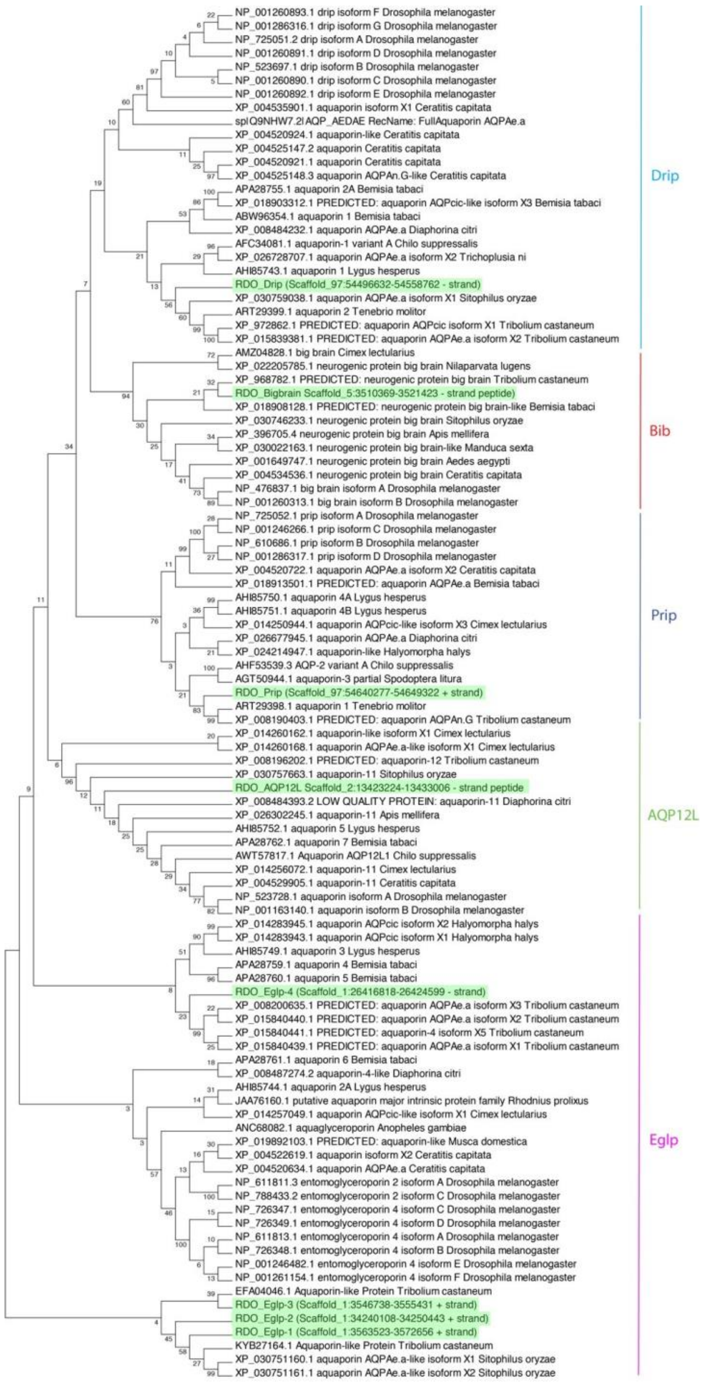

Supplementary Figure S4. Phylogenetic analysis of *R. dominica* aquaporins. The evolutionary history of representative insect aquaporins was inferred using the Neighbor-Joining method [195]. The bootstrap consensus tree inferred from 500 replicates is taken to represent the evolutionary history of the taxa analyzed [196]. Branches corresponding to partitions reproduced in less than 50% bootstrap replicates are collapsed. The percentage of replicate trees in which the associated taxa clustered together in the bootstrap test (500 replicates) are shown next to the branches. The evolutionary distances were computed using the Dayhoff matrix-based method [197] and are in the units of the number of amino acid substitutions per site. The analysis involved 101 amino acid sequences. All positions containing gaps and missing data were eliminated. There were a total of 88 positions in the final dataset. Evolutionary analyses were conducted in MEGA7 [67].
